# Karyopherin α2-dependent import of E2F1 and TFDP1 maintains protumorigenic stathmin expression in liver cancer

**DOI:** 10.1186/s12964-019-0456-x

**Published:** 2019-11-29

**Authors:** Elisabeth Drucker, Kerstin Holzer, Stefan Pusch, Juliane Winkler, Diego F. Calvisi, Eva Eiteneuer, Esther Herpel, Benjamin Goeppert, Stephanie Roessler, Alessandro Ori, Peter Schirmacher, Kai Breuhahn, Stephan Singer

**Affiliations:** 1 Institute of Pathology, University Hospital Heidelberg, Im Neuenheimer Feld 224, 69120 Heidelberg, Germany; 2 Institute of Pathology, University Medicine Greifswald, Friedrich-Loeffler-Straße 23e, 17475 Greifswald, Germany; 3 Department of Neuropathology, Institute of Pathology, University Hospital Heidelberg, Im Neuenheimer Feld 224, 69120 Heidelberg, Germany; 4 German Consortium of Translational Cancer Research (DKTK), Clinical Cooperation Unit Neuropathology, German Cancer Research Center (DKFZ), Im Neuenheimer Feld 280, 69120 Heidelberg, Germany; 5 Department of Anatomy, University of California, 513 Parnassus Avenue, San Francisco, CA 94143 USA; 6 Institute of Pathology, University Regensburg, Franz-Josef-Strauß-Allee 11, 93053 Regensburg, Germany; 7 European Molecular Biology Laboratory, Structural and Computational Biology Unit, Meyerhofstraße 1, 69117 Heidelberg, Germany; 8 Leibniz-Institute on Aging, Fritz-Lipmann-Institute (FLI), Beutenbergstraße 11, 07745 Jena, Germany

**Keywords:** Karyopherin, Stathmin, HCC, E2F1, TFDP1, Nuclear transport

## Abstract

**Background:**

Members of the karyopherin superfamily serve as nuclear transport receptors/adaptor proteins and provide exchange of macromolecules between the nucleo- and cytoplasm. Emerging evidence suggests a subset of karyopherins to be dysregulated in hepatocarcinogenesis including karyopherin-α2 (KPNA2). However, the functional and regulatory role of KPNA2 in liver cancer remains incompletely understood.

**Methods:**

Quantitative proteomics (LC-MS/MS, ~ 1750 proteins in total) was used to study changes in global protein abundance upon siRNA-mediated KPNA2 knockdown in HCC cells. Functional and mechanistic analyses included colony formation and 2D migration assays, co-immunoprecipitation (CoIP), chromatin immunoprecipitation (ChIP), qRT-PCR, immmunblotting, and subcellular fractionation. In vitro results were correlated with data derived from a murine HCC model and HCC patient samples (3 cohorts, *n* > 600 in total).

**Results:**

The proteomic approach revealed the pro-tumorigenic, microtubule (MT) interacting protein stathmin (*STMN1*) among the most downregulated proteins upon KPNA2 depletion in HCC cells. We further observed that KPNA2 knockdown leads to reduced tumor cell migration and colony formation of HCC cells, which could be phenocopied by direct knockdown of stathmin. As the underlying regulatory mechanism, we uncovered E2F1 and TFDP1 as transport substrates of KPNA2 being retained in the cytoplasm upon KPNA2 ablation, thereby resulting in reduced *STMN1* expression. Finally, murine and human HCC data indicate significant correlations of *STMN1* expression with E2F1/TFPD1 and with KPNA2 expression and their association with poor prognosis in HCC patients.

**Conclusion:**

Our data suggest that KPNA2 regulates *STMN1* by import of E2F1/TFDP1 and thereby provide a novel link between nuclear transport and MT-interacting proteins in HCC with functional and prognostic significance.

## Background

Hepatocellular carcinoma (HCC) ranks fifth among the most common malignancies worldwide and second among leading causes of cancer-related death [1]. The prognosis of HCC is poor and therapeutic options are limited including partial hepatectomy, liver transplantation, radio frequency ablation, transarterial chemoembolization (TACE) and Sorafenib for systemic treatment of advanced disease stage [2]. A more detailed understanding in particular of those molecular mechanisms that have not yet been in the primary focus of liver cancer related research such as alterations of the nuclear transport system (NTS), holds great potential for improved therapeutic approaches [3].

The NTS is essential for the exchange of macromolecules between the nucleus and the cytoplasm [3, 4]. The NTS includes importins and exportins mostly belonging to the karyopherin superfamily and components of the nuclear pore complex (NPC), termed Nucleoporins (Nups) [3]. The classical protein import pathway involves binding of cargo proteins containing a nuclear localization signal (NLS) to adaptor proteins of the karyopherin-α family which in turn interact with importin-β1 [4, 5]. This heterotrimeric complex enters the nucleus through the NPC and dissociates in a RanGTP-dependent fashion releasing its transport substrate. Karyopherin-α is then re-shuttled by exportin 2/Cellular apoptosis susceptibility (XPO2/CAS) to the cytoplasm [6] while importin-β1 is exported by binding to RanGDP [3, 4].

Altered nuclear transport factors in cancer have been primarily studied in the context of Nup-containing fusion proteins [7, 8], but are also observed in a variety of solid tumors including liver cancer [3, 9, 10]. Karyopherin-α2 (KPNA2; =importin alpha 1) is among the strongest overexpressed karyopherins in HCC, as previously described [9]. However, the functional aspects of KPNA2 in HCC and the underlying mechanisms by which KPNA2 supports tumorigenesis are poorly understood.

Highly dynamic turnover of the microtubule (MT) network is essential to tumor cell growth, migration, invasion and dissemination. MTs consist of α-tubulin and β-tubulin heterodimers and are characterized by a permanent transition (dynamic instability) between phases of depolymerization (catastrophe) and polymerization (rescue) [11]. MT-interacting proteins modulate the dynamic instability of MTs either by executing MT-stabilizing or -destabilizing functions. Stathmin (oncoprotein 18/OP18, *STMN1*) represents the prototype member of a MT-destabilizing phosphoprotein family that encompasses also stathmin-like 2 (superior cervical ganglion 10; SCG10, *STMN2*), stathmin-like 3 (SCG10-like protein; SCLIP, *STMN3*), and stathmin-like 4 (RB3, *STMN4*) [12]. Stathmin is the best characterized member of this protein family in the context of cancer biology [13] and has been described to facilitate tumor cell migration, invasion and colony formation in many cancer types [14–16] including HCC [17].

Here, we identified by proteome-wide analysis that KPNA2 is required for maintaining stathmin overexpression in liver cancer cells and dissected the underlying regulatory mechanism involving the nuclear import of the transcription factors E2F1 and TFDP1.

## Methods

### Cell culture

HLE and HLF cells (JCRB0404 and JCRB0405, both obtained from JCRB Cell Bank, Osaka, Japan) were cultured in Dulbecco’s Modified Eagle’s Medium (DMEM, obtained from Sigma-Aldrich, Taufkirchen, Germany) supplemented with 10% fetal calf serum and 1% penicillin/streptomycin (Life Technologies, Darmstadt, Germany) in an atmosphere containing 5% CO_2_.

### siRNA-transfections

Small interfering RNAs (siRNAs) KPNA2#1 (5′-AAUCUUACCUGGACACUUU-3′) and KPNA2#2 (5′-UUCGUUAAGCUUAAUUGAGAA-3′), STMN1#1 (5′-AGGCAAUAGAAGAGAACAA-3′) and STMN1#2 (5′-AAGAGAAACUGACCCACAA-3′), E2F1#1 (5′-AACUCCUCGCAGAUCGUCAUC-3′) and E2F1#2 (5′-CAGAUCUCCCUUAAGAGCAAA-3′), TFDP1#1 (5′-CAGAACCTTAGTCCCGGGAAA-3′) and TFDP1#2 (5′-CACATTTGAAATCCACGATGA-3′), c-JUN#1 (5′-AAGAACGTGACAGATGAGCAG-3′) and c-JUN#2 (5′-CCCGAGCTGGAGCGCCTGATA-3′) were purchased from Eurofins MWG Operon (Ebersberg, Germany). As negative control siRNA for all knockdown experiments the QIAGEN All-Stars duplex (Hilden, Germany) was used. The transfections were performed according to the manufacturer’s instructions using Oligofectamine (Invitrogen, Karlsruhe, Germany) with a final siRNA concentration of 50 nM. For siRNA pools, the two respective siRNAs were combined at a concentration of 25 nM each to reach a final concentration of 50 nM.

### Immunoblotting

Immunoblotting was performed as previously described [9]. In brief, whole protein lysates were separated by SDS/PAGE and transferred to nitrocellulose membranes (Whatman, Dassel, Germany). Membranes were incubated with the following primary antibodies diluted in 5% Milk/TBST-containing blocking solution overnight: anti-KPNA2 (rabbit polyclonal, 1:2000; abcam, Cambridge, UK), anti-stathmin (rabbit monoclonal, 1:1000; abcam), anti-E2F1 (rabbit polyclonal, 1:200; Santa Cruz, Heidelberg, Germany), anti-TFDP1 (rabbit polyclonal, 1:500; abcam), anti-ATF-2 (rabbit polyclonal, 1:200; Santa Cruz), anti-FBP-1/2 (goat polyclonal, 1:200; Santa Cruz), anti-c-JUN (rabbit monoclonal, 1:2000; Cell Signaling Technology, Frankfurt, Germany), anti-HMOX1 (rabbit monoclonal, 1:10,000; abcam), anti-GTSF1 (goat polyclonal, 1:200; Santa Cruz), anti-PARP (rabbit polyclonal, 1:500; Cell Signaling Technology), anti-β-tubulin (mouse monoclonal, 1:1000; Becton, Dickinson and Company, Franklin Lakes, USA) and anti-β-actin (mouse monoclonal, 1:10,000; MP Biomedicals, Illkirch, France). Blots were incubated with fluorescence-conjugated secondary antibodies (LI-COR Bioscience, Bad Homburg, Germany) for one hour and detection was performed using the Odysee Sa Infrared Imaging System (LI-COR Bioscience).

### Subcellular fractionation

NE-PER Nuclear and Cytoplasmic Extraction Reagents (Thermo Scientific, Offenbach, Germany) were used according to the manufacturer’s protocol with an additional washing step after isolation of the cytoplasmic fraction. Protein lysates were immunoblotted as described above.

### Molecular cloning

The Gateway Cloning System (Thermo Fisher Scientific, Waltham, USA) was used to clone vectors for KPNA2, E2F1 and TFDP1 overexpression. In a first step, the cDNAs obtained from whole cell lysates were PCR amplified using attB-flanked primers for the respective insert and Gateway recombination according to the manufacturer’s protocol. PCR products were separated using agarose gel electrophoresis and the relevant fragments were isolated and purified. In the following BP reaction, the PCR product was sub-cloned into pDONR201 which was used as entry clone. In a final step, the respective genes were recombined into the expression vectors pDEST26-N-HA or pDEST26-N-FLAG by LR reaction. Final plasmids were verified by sanger sequencing.

### Co-Immunoprecipitation

Co-Immunoprecipitation (CoIP) was performed as previously described [18]. In brief, HLE or HLF cells were transfected with a combination of N-terminally HA-tagged KPNA2 and Flag-tagged E2F1 or TFDP1 plasmids using the FuGENE HD Transfection Reagent (Promega, Mannheim, Germany) and harvested 24 h later in non-denaturing lysis buffer. Dynabeads Protein G (Invitrogen, Karlsruhe, Germany) were incubated with the respective antibody for four hours or IgG1 (Santa Cruz) as negative control and subsequently with whole protein lysates overnight at 4 °C on an overhead rotator. Immunoprecipitated proteins were eluted by shaking of Dynabeads in 1x Laemmli buffer for 20 min at ambient temperature. Eluted CoIP lysates were separated using SDS/PAGE and transferred to nitrocellulose membranes (Whatman) as described above.

### Total RNA isolation, cDNA synthesis and quantitative real-time polymerase chain reaction

The NucleoSpin RNA II kit (Macherey-Nagel, Dueren, Germany) was used for total RNA isolation. cDNA was synthesized by reverse transcription of 1 μg RNA using the Sigma-Aldrich RT-PCR kit according to the manufacturer’s protocol. Samples were analyzed in technical duplicates on a StepOnePlus real-time PCR device (Applied Biosystems, Darmstadt, Germany) using the PrimaQUANT qPCR CYBR-Green-MasterMix-high-ROX (Steinbrenner, Heidelberg, Germany). Expression levels were normalized to those of RPL32 using the ΔΔCt method. Primers were designed manually and obtained from ThermoFisher Scientific (Offenbach, Germany) with the following exon-exon-spanning sequences: RPL32-for 5′-TTCCGGTCCACAACGTCAAG-3‘; RPL32-rev 5’-TGTGAGCGATCTCGGCAC-3‘; KPNA2-for 5’-AGGAAAACCGCAACAACCAG-3′; KPNA2-rev 5′-ACCAGCCCGGATTATGTTGT-3′; STMN1-for 5′-TGCAGAATACACTGCCTGTC-3′; STMN1-rev 5′-AGGCACGCTTCTCCAGTTCT-3′; E2F1-for 5′-GCCAAGAAGTCCAAGAACCAC-3′; E2F1-rev 5′-CGCAGCTGCGTAGTACAGATATTC-3′; TFDP1-for 5′-GTAGGAAGCCCACACACCCCCA-3′; TFDP1-rev 5′-GAAATGCCGTAGGCCCTTGCCA-3′.

### Chromatin immunoprecipitation

Chromatin immunoprecipitation (ChIP) assay was performed as previously described [19] to study binding of E2F1 and TFDP1 to the *STMN1* promoter region. In brief, HLE cells were seeded onto 15 cm dishes, protein and DNA were crosslinked by incubation of cells with 1% formaldehyde/PBS and quenched with 125 mM glycine. Subsequently, cells were harvested in RIPA buffer and sonicated to fragment genomic DNA. After preclearing, samples were mixed with an E2F1 (mouse monoclonal, 3 μg; Millipore, Burlington, USA) or TFDP1 (mouse monoclonal, 3 μg; LifeSpan BioSciences, Seattle, USA) antibody or IgG as a control and blocked Dynabeads and incubated overnight at 4 °C. The next day, the protein-DNA complexes were eluted from the Dynabeads and the protein-DNA crosslinking was reversed by addition of 4 M NaCl. DNA was purified using the NucleoSpin® Gel and PCR Clean-up Kit (Macherey-Nagel) according to the manufacturer’s protocol. Precipitated DNA was quantified using qPCR based on a genomic DNA standard curve. ChIP primers were designed according to predicted binding site sequences obtained from publicly available ChIP-Seq datasets following E2F1 precipitation (accessible via https://www.encodeproject.org). As a negative control, a random sequence upstream of the predicted binding sequence was additionally quantified, a previously reported E2F1 binding site within the *CDC2* promoter served as positive control. Sequences of ChIP-Primers were as follows: E2F1-*STMN1* Promoter Binding Site 1: for 5′-ACCCACCTGCTCAGTCCG-3′, rev 5′-CGGGTCTGTTGGTGCTCAGAG-3′; E2F1-*STMN1* Promoter Binding Site 2: for 5′-CTCCCCGCGCCTTTTCGAATC-3′, rev 5′-GGCTCCGGGGTGTTGAGTTC-3′; negative control: for 5′-CACAACCCAGGAGGGAAACAG-3′, rev 5′-CACCCTGTTCTGACTTGGATGC-3′; E2F1-*CDC2* Promoter Binding Site: for 5′-CGCCCTTTCCTCTTTCTTTC-3′, rev 5′- ATCGGGTAGCCCGTAGACTT-3′.

### Migration assay

A two-dimensional “scratch” assay two days upon siRNA-mediated knockdown of either KPNA2 or stathmin was used to measure tumor cell migration as previously described [10]. In brief, HLE cells were treated with mitomycin C (5 μg/mL) for 3 h (repressing cell proliferation) before the cell monolayer was scratched using a pipette tip. Incubation of cells with hepatocyte growth factor (HGF, 10 ng/mL) for 18 h was used to induce migration. The relative migratory capacity was determined by calculating the percentage of the cell-free area.

### Colony formation assay

Clonogenic capacity upon siRNA-mediated depletion of KPNA2 or stathmin was analyzed using a colony formation assay. After knockdown, cells were re-seeded into a 6-well plate at a low density (HLE: 500 cells/well; HLF: 1000 cells/well) and colonies were stained using a 1% crystal violet solution 14 days after siRNA-treatment. Colonies were counted and the relative clonogenic capacity was evaluated compared to the All-Stars control.

### Proteomic analyses

Quantitaive mass spectrometry was conducted as recently described [20]. In brief, lysates isolated from HLE cells three days upon KPNA2 siRNA treatment were processed and analyzed in triplicates by liquid chromatography-tandem mass spectrometry (LC-MS/MS). Peptides were assessed using a nano-Acquity UPLC system (Waters, Eschborn, Germany) connected online to a LTQ-Orbitrap Velos Pro instrument (Thermo Fisher Scientific, Bremen, Germany).

### Tumor tissue samples, gene expression data, and immunohistochemically analysis

Our study used a published Affymetrix U133A2.0 gene expression data set derived from 256 HCC patients including 247 tumor and 239 adjacent non-neoplastic samples, as described by Roessler et al. (Gene Expression Omnibus accession number GSE14520) [21, 22]. Complementary, the publicly available gene expression data of the TCGA LIHC cohort (the cancer genome atlas, liver hepatocellular carcinoma, accessible via: http://cancergenome.nih.gov), which contains data of 371 HCC patients, was analyzed.

The HCC tissue microarray (containing 95 HCC FFPE samples with the following tumor grade: 14 x G1, 52 x G2, 27 x G3, 2 x G4) was provided by the Tissue Bank of the National Center for Tumor Diseases (NCT) Heidelberg. The sample use was approved by the local Ethics Committee. Immunohistochemical staining (IHC) was performed as described previously [10], using an anti-KPNA2 (rabbit polyclonal, 1:50; abcam) and anti-stathmin (rabbit monoclonal, 1:50; abcam) antibody. Scoring was conducted as described before [9].

FFPE tissue samples of the E2F1 driven murine HCCs (11 male mice, age 9–15 months), engineered and characterized by the Thorgeirsson Laboratory [23], were kindly provided by D. Calvisi. Full sections were immunohistochemically stained using the anti-stathmin antibody (1:50) based on the same staining protocol being conducted for the tissue microarray.

### Statistical analysis and software

Data are presented as the mean ± standard deviation (SD). Expression differences between HCC and non-tumorous liver samples and cell culture samples were assessed by nonparametric two-tailed Mann-Whitney U tests using SPSS Statistics24 (IBM, Ehningen, Germany) if not indicated otherwise. Spearman’s rank correlation coefficient of the respective gene expression in the tumor tissues was calculated with GraphPad Prism 6 (GraphPad Software, Inc., La Jolla, USA). Overall survival data was analyzed by Kaplan-Meier curves and log-rank *p*-values using GraphPad Prism 6. *P*-values < 0.05 were considered significant.

## Results

### Proteomic approach reveals stathmin to be downregulated upon KPNA2 depletion

**Fig. 1:**
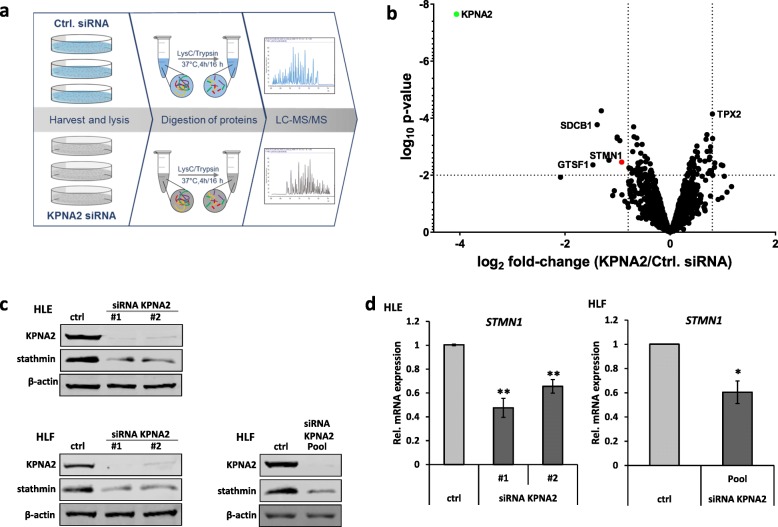
Stathmin is downregulated upon KPNA2 depletion. **a** Workflow of LC-MS/MS analysis. HLE cells were harvested 72 h after control (Ctrl.) or KPNA2 siRNA treatment (*n* = 3). **b** Volcano-Plot illustrates the resulting log_2_ fold-changes (KPNA2/Ctrl. siRNA) and corresponding log_10_ *p*-values of 1759 proteins being quantified by the LC-MS/MS analysis. Horizontal dotted line *p* = 0.01; vertical dotted lines log_2_ fold-change 0.8 or − 0.8; green dot: KPNA2; red dot: stathmin. **c** HLE and HLF cells were siRNA-treated and harvested as described in (**a**). Lysates were immunoblotted using the indicated antibodies. **d** HLE and HLF cells were treated as described in (**a**) and *STMN1* expression was analyzed by qRT-PCR. (HLE: *n* = 5, *p* < 0.01 (**); HLF: *n* = 4, *p* < 0.05(*))

To identify potential „downstream” targets of KPNA2 in HCC cells, we performed quantitative mass spectrometry (LC-MS/MS) measuring 1759 proteins in HLE cells upon KPNA2 siRNA treatment compared to control siRNA (Ctrl.) treated cells (Fig. 1a and b as well as Additional file 2: Table S1 and Additional file 3: Table S2). As illustrated by the volcano plot (Fig. 1b), besides KPNA2 as the primary knockdown target (green dot), the MT-interacting protein stathmin (STMN1, red dot) was among the most pronounced reduced proteins. Out of several interesting proteins dysregulated upon KPNA2 depletion, such as Gametocyte-specific factor 1 (GTSF1), Syntenin-1 (SDCB1, for validation see Additional file 1: Figure S1), and Targeting protein for *Xenopus* kinesin-like protein 2 (TPX2, see also Discussion and Additional file 2: Table S1), we chose stathmin for further validation because of its well documented protumorigenic role in (hepato-)carcinogenesis [14–17, 24]. Consistent with the proteomic data we could confirm decreased stathmin protein levels upon KPNA2 knockdown (including an additional KPNA2 siRNA) not only in HLE, but also in the HCC cell line HLF, by immunoblotting (Fig. 1c). Moreover, decreased stathmin protein was also paralleled by significantly reduced *STMN1* mRNA as quantified by qRT-PCR (Fig. 1d). Interestingly, other stathmin family members (STMN2–4) were below the level of detection as indicated by Western blot analysis (data not shown). Taken together, we could identify and validate stathmin as a „downstream” target of KPNA2 at the protein and transcript level in HCC.

### KPNA2 is required for colony formation and tumor cell migration in HCC cells

**Fig. 2:**
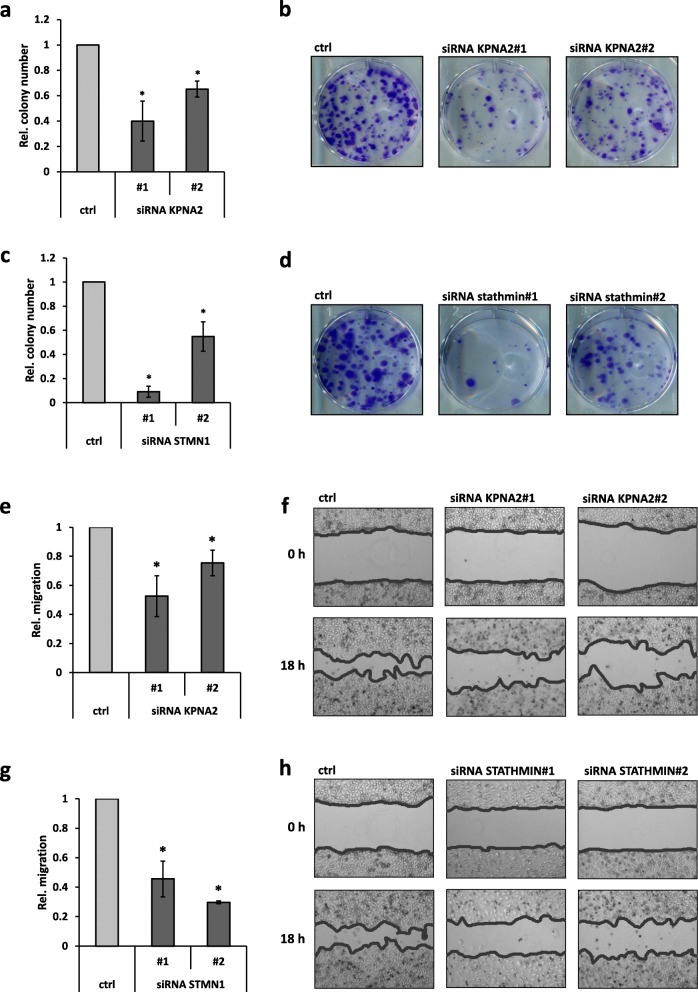
KPNA2 depletion reduces the clonogenic capacity and cell migration of HCC cells being phenocopied by stathmin knockdown. **a**, **b** HLE cells were treated either with ctrl. or KPNA2 siRNAs and colony formation was analyzed 14 days after treatment by crystal violet staining (n = 4; p < 0.05 (*)). **c, d** HLE cells were treated either with ctrl. or stathmin siRNAs and colony formation assays were performed as described in (**a**) (n = 4; p < 0.05 (*)). **e, f** HLE cells were treated as described in (**a**) and two-dimensional scratch assays were started 48 h later. Gap closure was analyzed 18 h after scratching (n = 4; p < 0.05 (*)). **g, h** HLE cells were treated as described in (**c**) and scratch assays were performed as described in (**e**) (n = 4; p < 0.05 (*))

A variety of protumorigenic functions of stathmin have been previously documented including tumor cell proliferation/colony formation and migration [14–17]. We therefore hypothesized that KPNA2 knockdown and the associated decrease of stathmin are followed by reduced clonogenic capacity and migration of HCC cells. To this end, HLE cells were plated at a very low density in the presence or absence of KPNA2 or stathmin and the ability to form colonies was evaluated after 14 days by crystal violet staining. As shown in Fig. 2a and b, knockdown of KPNA2, indeed, resulted in significantly less colonies being formed compared to the controls. An even more dramatic effect occurred upon direct stathmin depletion with further reduced numbers of colonies (Fig. 2c and d, for validation of stathmin siRNAs see Additional file 1: Figure S2a). Substantiating these findings, the effects of KPNA2 and stathmin depletion on clonogenicity could be recapitulated in HLF cells (Additional file 1: Figure S2a-e). Next, we performed two-dimensional scratch assays and monitored gap closure in control or KPNA2 siRNA treated cells. For these assays tumor cell proliferation was blocked by Mitomycin C treatment. In line with our hypothesis, tumor cell migration was significantly reduced in both KPNA2 siRNA conditions as indicated by an up to 50% lower gap closure (Fig. 2e and f). Knockdown of stathmin with two different siRNAs decreased the migratory capacity in HLE cells even more drastically by up to 70% (Fig. 2g and h) most likely due to a more pronounced reduction of stathmin protein in the direct knockdown condition compared to KPNA2 depleted condition. We conclude that KPNA2 is required for the full capacity of HCC cells to form colonies and to migrate by maintaining stathmin expression.

### KPNA2 regulates *STMN1* transcription by mediating the nuclear import of E2F1 and TFDP1

**Fig. 3:**
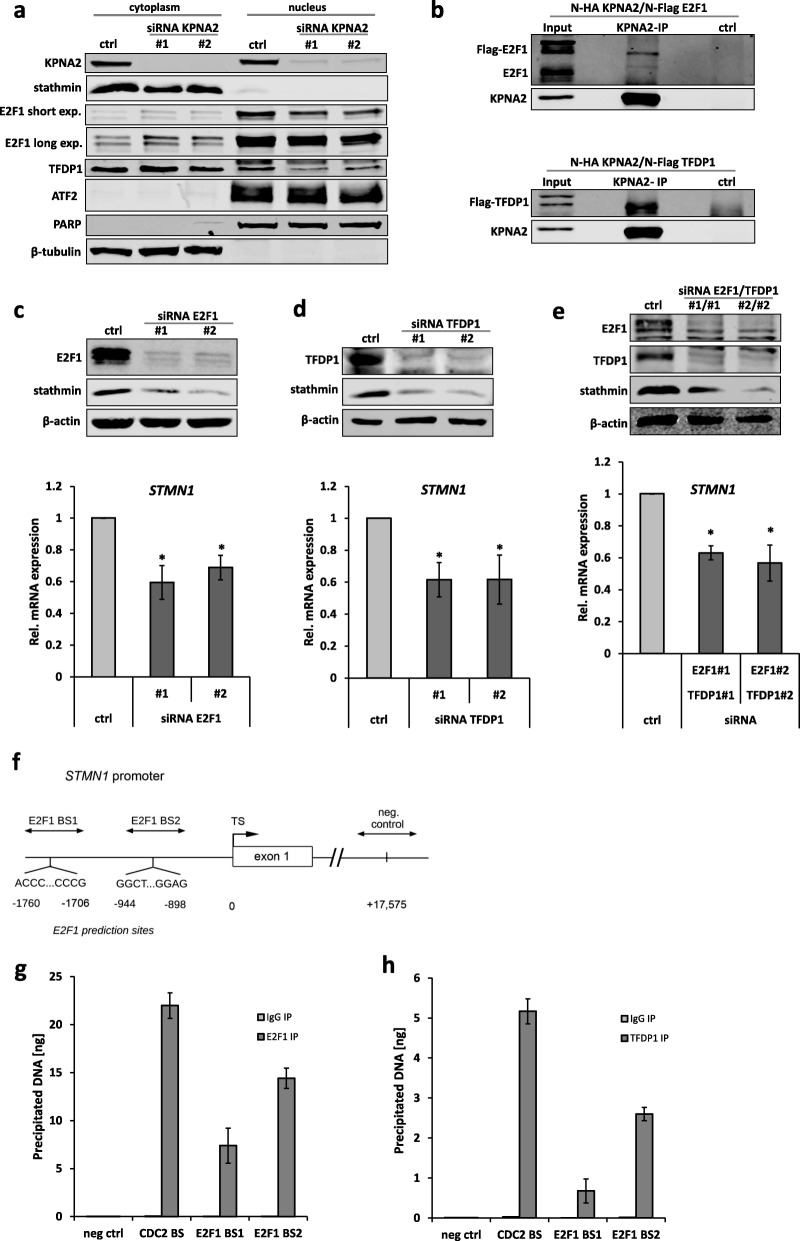
KPNA2 regulates *STMN1* by import of the transcription factors E2F1 and TFDP1. **a** HLE cells were treated with ctrl. or KPNA2 siRNAs and nuclear-cytoplasmic fractionation was performed after 72 h. Samples were immunoblotted using the indicated antibodies. **b** HLE cells were co-transfected with HA-tagged KPNA2 and Flag-tagged E2F1 or TFDP1. KPNA2 immunoprecipitation was performed and samples were immunoblotted using the indicated antibodies. **c, d** HLE cells were treated with ctrl. siRNA or siRNAs directed against E2F1 or TFDP1 and *STMN1* expression was analyzed by immunoblotting (upper panel) or qRT-PCR (lower panel, n = 4; p < 0.05 (*)). **e** HLE cells were treated with siRNAs directed against E2F1 and TFDP1 and *STMN1* expression was analyzed by immunoblotting (upper panel) or qRT-PCR (lower panel, n = 4; p < 0.05 (*)). **f** Illustration of the predicted E2F1 binding sites (BS) within the promoter region of *STMN1*. A non-coding region downstream of the promoter region served as negative control. **g** E2F1 was immunoprecipitated in HLE cells, ChIP assay was performed and precipitated DNA of the predicted *STMN1* bindings sites, the positive control binding site (*CDC2*) and a control region (neg ctrl) was quantified using qRT-PCR. The bar diagram depicts one representative experiment. **h** TFDP1 was immunoprecipitated in HLE cells and ChIP assay was performed as described in (**g**). The bar diagram depicts one representative experiment

Next, we set out to determine the molecular mechanism by which KPNA2 regulates stathmin. We hypothesized that the nuclear import of transcription factors (TFs) controlling *STMN1* mRNA expression could be dependent on KPNA2. Accordingly, KPNA2 depletion would result in an import defect of relevant TFs followed by reduced *STMN1* expression. Potentially relevant TFs were selected based on literature and database mining (e.g. Promo 3.0, accessible via http://alggen.lsi.upc.es/cgi-bin/promo_v3/promo/promoinit.cgi?dirDB=TF_8.3 and TFBIND, accessible via http://tfbind.hgc.jp/) and evaluated by fractionation regarding their subcellular distribution in KPNA2 or control siRNA treated HLE cells. Analysis of FBP-1 and -2 revealed no alteration in subcellular localization following KPNA2 depletion, however, for c-JUN an accumulation in the cytoplasmic fraction along with a decreased abundance in the nuclear fraction was observed (Additional file 1: Figure S3a). Subsequent Co-Immunoprecipitation (CoIP) experiments verified direct physical binding of KPNA2 and c-JUN (Additional file 1: Figure S3b), however, direct c-JUN knockdown did not lead to reduced *STMN1* expression as quantified by qRT-PCR (Additional file 1: Figure S3c). Therefore, E2F1 and TFDP1, which have been previously reported to form dimers and to be involved in stathmin regulation [25], were assayed. In line with our hypothesis, E2F1 and TFDP1 were both increased in the cytoplasmic and decreased in the nuclear fraction after KPNA2 silencing, respectively (Fig. 3a). Excluding a general import defect of TFs by KPNA2 knockdown ATF2 was unchanged in the respective fractions, serving as a negative control (Fig. 3a). Consistent with the aforementioned findings, we could detect a physical interaction of KPNA2 with E2F1 and TFDP1 by CoIP confirming both TFs as transport substrates of KPNA2 in HCC cells (Fig. 3b and Additional file 1: Figure S4a). In addition, we could demonstrate that direct knockdown of E2F1 and/or TFDP1, indeed, downregulates *STMN1*. Figure 3c and d show that siRNA mediated depletion of E2F1 or TFDP1 significantly reduced stathmin protein and transcript levels in HLE cells as quantified by immunoblotting and qRT-PCR. Reduced *STMN1* expression upon E2F1 and TFDP1 knockdown was also recapitulated in HLF cells (Additional file 1: Figure S4b and c). Interestingly, the effect size of a combined E2F1 and TFDP1 depletion was not different to the single knockdowns (Fig. 3e and Additional file 1: Figure S4d). To verify direct binding of E2F1 and TFDP1 to the *STMN1* promoter, chromatin immunoprecipitation (ChIP) assays were performed. Analysis of publicly available ChIP-Seq datasets (accessible via https://www.encodeproject.org) indicated two binding sites for E2F1 within the regulatory region of *STMN1* (Fig. 3f). A non-coding region downstream of the *STMN1* promoter served as negative control, binding to a previously reported region within the *CDC2* promoter [26] as positive control. Indeed, following immunoprecipitation of E2F1, up to 15 ng of DNA containing the predicted *STMN1* promoter binding site was precipitated as quantified by qRT-PCR (Fig. 3g and Additional file 1: Figure S4e). Moreover, also binding of TFDP1 to the predicted E2F1 binding sites within the *STMN1* promoter was observed (Fig. 3h and Additional file 1: Figure S4f). Together, these data indicate that KPNA2 controls *STMN1* expression in HCC cells via the nuclear import of E2F1 and TFDP1.

Finally, we evaluated if our in vitro findings can be transferred to the in vivo situation. We could substantiate E2F1 and TFDP1 as important regulators of *STMN1* in HCC by the following findings. In an E2F1-driven transgenic HCC mouse model engineered by Conner et al. in which the interaction with TFDP1 was validated [23], we identified strikingly higher stathmin immunoreactivity not only in the full-blown tumors (Additional file 1: Figure S5a, left column) but also in the precursor lesions (Additional file 1: Figure S5a, right column) compared to the adjacent liver tissue. With variable staining intensity all liver tumor nodules that developed in each of the overall 11 E2F1-transgenic mice were positive for stathmin (Additional file 1: Figure S5b).

**Fig. 4:**
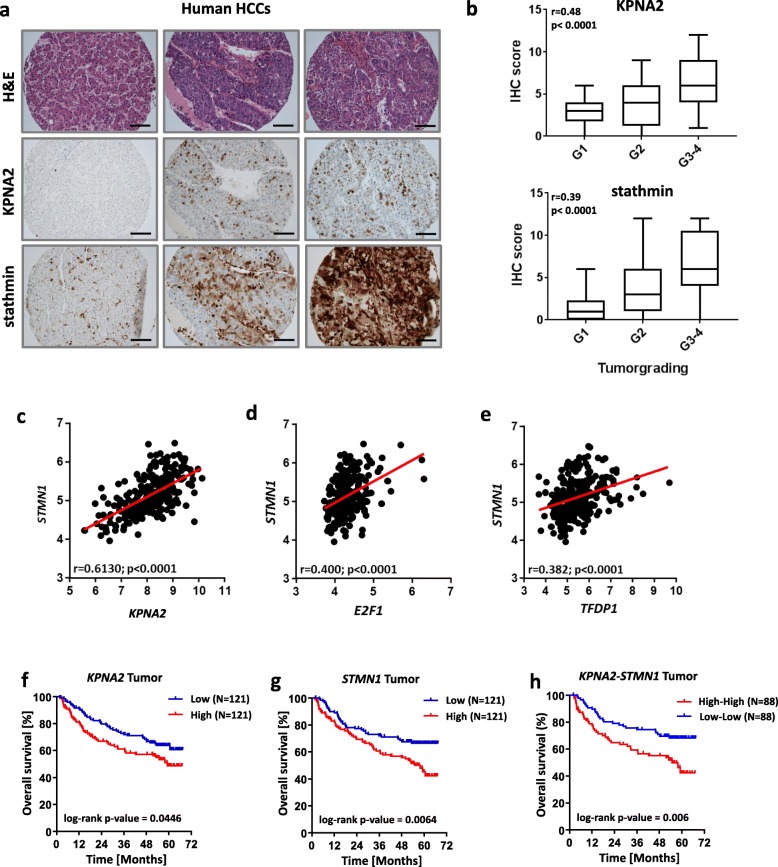
KPNA2 and stathmin/*STMN1* expression are correlated in human HCC. **a** Representative micrographs show human HCC samples either H&E stained (upper row) or immunostained with KPNA2 (middle row) or stathmin (lower row). Scale bar: 100 μm. **b** Boxplots illustrate increasing immunohistochemical (IHC) scores of KPNA2 (upper panel) or stathmin (lower panel) with tumor dedifferentiation (G1 = well differentiated, G2 = moderately differentiated, G3–4 = poorly differentiated). **c** Spearman correlation between *KPNA2* and *STMN1* mRNA expression in a large HCC cohort (Roessler cohort). ***STMN1*** **is correlated to** ***E2F1*** **and** ***TFDP1*** **in human HCC. d, e** Spearman correlation between *STMN1* and *E2F1* **(d)** *or TFDP1* **(e)** expression in human HCC samples (Roessler cohort). **High expression of** ***KPNA2*** **and** ***STMN1*** **correlates with poor prognosis in HCC patients.** Overall survival of HCC patients showing low and high mRNA expression of *KPNA2* **(f)** and *STMN1* **(g)** or both **(h)** (cut-off: median, Roessler cohort)

Furthermore, supporting the relevance of KPNA2-dependent stathmin regulation in human HCC samples, we found a strong and highly significant spearman correlation (r = 0.73; *p* < 0.0001) between the immunoreactivity (IHC scores) of both factors using a tissue microarray (TMA) containing 95 human HCCs (Fig. 4a). Moreover, KPNA2 and stathmin were positively correlated with tumor grading (r = 0.48; p < 0.0001 and r = 0.39; p < 0.0001, respectively) as shown in Fig. 4b. The correlation between *KPNA2* and *STMN1* could also be confirmed in HCC tissues of two larger patient cohorts (Roessler cohort, *n* = 247; TCGA LIHC cohort, *n* = 371) at the mRNA level reflected by Spearman correlation coefficients of r = 0.61 (p < 0.0001; Fig. 4c) and r = 0.63 (p < 0.0001; Additional file 1: Figure S6a), with both factors being overexpressed in HCC (Additional file 1: Figure S6b). Moreover, even in human HCC samples of the Roessler cohort with more complex and diverse genetic alterations, we could detect a Spearman correlation of *STMN1* with *E2F1* and *TFDP1* (r = 0.40, p < 0.0001, Fig. 4d and r = 0.38, p < 0.0001, Fig. 4e). Importantly, no relevant correlations (thresholds: r < − 0.3 or r > 0.3 and *p* < 0.05) were found between *STMN1* and other *KPNA*s (*KPNA1, KPNA3, KPNA4, KPNA5* and *KPNA6,* Additional file 1: Figure S6c) or between *KPNA2* and other *STMN*s (*STMN2, STMN3,* and *STMN4,* Additional file 1: Figure S6d*)*. These data underscore the specific correlation between *KPNA2* and *STMN1* in this context*.*

Interestingly, *KPNA2* was variably correlated with other MT-associated factors (*TPX2* r = 0.77, p < 0.0001; *KIF2A* r = 0.51, p < 0.0001; *CLIP1* r = 0.13, *p* < 0.034; MAP 4 r = 0.25, p < 0.0001, Additional file 1: Figure S6e) indicating that beyond the stathmin family a more complex interplay between KPNA2 and MT-interactors can be assumed (see also Discussion).

Lastly, based on the reduced migratory capacity and clonogenic potential of HCC cells upon KPNA2 and stathmin knockdown we assumed that high expression of both factors correlates with more aggressive tumor behavior. To support this assumption, we performed Kaplan-Meier analyses using survival data derived from the two large HCC cohorts. Indeed, a higher than median expression of *KPNA2* (Fig. 4f and Additional file 1: Figure S7a) or *STMN1* (Fig. 4g and Additional file 1: Figure S7b) and particularly of both factors (Fig. 4h and Additional file 1: Figure S7c) was associated with significantly poorer patient outcome.

**Fig. 5:**
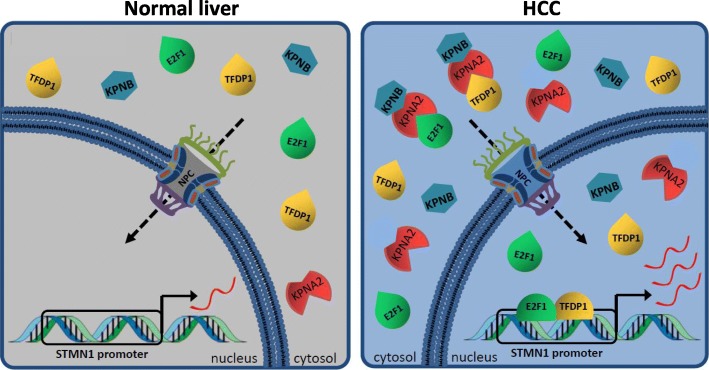
KPNA2 drives protumorigenic *STMN1* expression by nuclear import of the transcription factors E2F1 and TFDP1. E2F1 and TFDP1 form a heterotrimeric complex with KPNA2 and importin β1 (KPNB) which translocates into the nucleus through the nuclear pore complex (NPC). Upon dissociation of the complex E2F1 and TFDP1 bind to the *STMN1* promoter and drive *STMN1* expression (red wavy lines = *STMN1* mRNA). Compared to a normal, healthy liver KPNA2 is overexpressed in HCC, resulting in accelerated E2F1/TFDP1-mediated *STMN1* transcription

In summary, our data indicate a functionally relevant mechanism by which KPNA2 drives protumorigenic *STMN1* expression via the import of E2F1 and TFDP1 in HCC (Fig. 5).

## Discussion

Several mechanisms have been proposed and/or demonstrated by which NTS members can affect cancer-relevant genes and pathways [3, 25]. Among these the nuclear import of activated TFs appears of utmost importance as a key event in many if not all cancer signaling cascades [3]. Depending on the size/molecular weight of the respective TFs and other determinants the translocation from the cytoplasm to the nucleus through the NPC occurs in a nuclear transport receptor (NTR)-dependent and -independent fashion [3]. For instance, while the nuclear import of β-catenin (WNT signaling pathway) and SMAD2–4 (TGF β pathway) is NTR-independent, the nuclear import of STAT 1–3 (JAK/STAT pathway), and TFs of the NFkB pathway (p52, p65, c-Rel and RelB) is NTR-dependent [3]. A more comprehensive insight in NTR/cargo specificities and redundancies have been recently achieved by large scale interactome studies. Mackmull et al. have described the global interactome of many relevant nuclear transport receptors including KPNA2 using a proximity ligation (BioID) approach [26]. For KPNA2 the TFs TFDP1 and two E2F family members, i.e. E2F3 and E2F6, could be identified as interaction partners, however, E2F1 did not emerge in the KPNA2 interactome using the BioID technique. The proximity ligation study was performed in HEK293 cells (human embryonic kidney cells), while cell fractionation and CoIP experiments of our study were performed in HCC cell lines, which could explain the differences. Consistent with our results, Wang et al. [27] demonstrated E2F1 as a transport cargo of KPNA2 in non-small cell lung cancer (NSCLC). Thus, cell type-specific variations are the most likely explanation for these disparate findings and indicate the necessity of liver cancer-specific NTR-interactome studies.

The proteomic approach performed in this study suggested several additional proteins to be deregulated upon KPNA2 depletion, besides stathmin. Thus, it is intriguing to speculate about their role in the given context, even though for these proteins (similar to stathmin with an adjusted *p*-value > 0.05) validation experiments are required, before firm conclusion about their dysregulation can be drawn. Among these with a log_2_ fold change of − 1.47 was gametocyte-specific factor 1 (GTSF1), which is a factor involved in spermatogenesis and retrotransposon transcription in male germ cells [28]. In addition, GTSF1 was demonstrated to be overexpressed at the transcript level in HCC [29]. The same study also found that siRNA-mediated GTSF1 knockdown reduced tumor cell growth in a xenograft mouse model. To some extent similar findings were reported for Syntenin-1, which showed a log_2_ fold change of − 1.39 in our proteomic data set. Syntenin-1 is a multifunctional adaptor protein with various functions including cell adhesion and signal transduction [30]. In a cancer context, Syntenin-1 was demonstrated to positively regulate TGF β1 mediated SMAD2/3 activation and EMT transition [30] and to enhance cell surface expression of TGFR1 [31]. Liu et al. found overexpression of Syntenin-1 in HCC cell lines compared to non-tumorous liver cells (THLE3) and its overexpression was associated with increased proliferation and colony formation [32]. Among upregulated proteins was TPX2, another MT-associated factor, which is a spindle assembly factor and inactivated by binding to KPNA2 [33]. Upon its release from KPNA2, TPX2 activates AURKA kinase and mediates AURKA localization to the spindle microtubules and promotes microtubule nucleation [34, 35]. Rather counterintuitive based on our proteomic approach, but consistent with the correlation analyses in HCC patients (Additional file 1: Figure S6e), is the fact that TPX2 knockdown reduced cell migration and that TPX2 overexpression correlates with poor outcome as reported by Liu et al. [36]. Although hypothetical at this point, upregulation of TPX2 observed in our study upon KPNA2 knockdown could represent a short-term counter-regulatory response of HCC cells to compensate the functional defects resulting from the decrease of stathmin and other factors, which might in the long run be followed by a decrease of TPX2. A difference between short- and long-term responses upon KPNA2 knockdown may also apply to Kinesin Family Member 2A (*KIF2A,* a MT-dependent motor), which was considerably correlated with *KPNA2* expression in the HCC patient cohort (r = 0.51, *p* < 0.0001), but unchanged in the KPNA2 siRNA condition in the proteomic data set. In addition, cell line specific effects may also be taken into account. For the microtubules associated protein 4 (MAP 4) and CAP-Gly domain-containing linker protein 1 (*CLIP1*) consistent data were obtained with both factors being unaffected by KPNA2 knockdown and not being correlated to *KPNA2* in the HCC patient cohort. Taken together, these findings suggest that the functional and regulatory role of KPNA2 in HCC is multilayered and not limited to stathmin and therefore requires further investigations.

Besides E2F1 and TFDP1, other TFs involved in stathmin regulation need to be considered, as transcription of *STMN1* is not completely abolished upon depletion of either E2F1 or TFDP1. Other KPNA2 interacting TFs may play a minor role in this context since the effects of KPNA2 knockdown on *STMN1* mRNA reduction is largely similar to the knockdown of E2F1 and/or TFDP1. In contrast, TFs imported in an KPNA2-independent manner are most likely to drive the residual level of *STMN1* expression. For instance, the nuclear import of FUSE binding protein 1 (FBP1), previously demonstrated to be critically involved in transcriptional regulation of *STMN1* [16, 17], is dependent on KPNA1 [37]. Since KPNA1 is also overexpressed in HCC (similar to KPNA2) it will most likely also contribute to *STMN1* expression. Nevertheless, the strong correlation of KPNA2 and stathmin in human HCC underscores that the KPNA2-E2F1/TFDP1-stathmin axis is relevant in a significant fraction of HCCs.

The clinical significance of KPNA2 and stathmin could be recapitulated in two independent HCC patient cohorts consisting together of more than 600 HCC samples of different etiological backgrounds. Data together with previous findings [9, 38, 39] indicate that blocking KPNA-dependent protein import could represent a promising therapeutic approach. All KPNA family members are exported from the nucleus by exportin-2, which is also highly expressed and functionally relevant in HCC. Therefore, disrupting the interaction of KPNAs with exportin-2 (XPO2) could be a straight forward therapeutic strategy. While selective inhibitors of nuclear export (SINE) compounds targeting exportin-1 such as Selinexor are already in clinical trials, compounds directed against XPO2 are just about to emerge [38]. Recently, Tian et al. have identified gambogic acid as a covalent inhibitor of XPO2-mediated transport by a proteomic approach [38]. Accordingly, in validation experiments they could demonstrate by immunofluorescence that KPNA2 was accumulating in the nucleus upon treatment with gambogic acid. Thus, gambogic acid successfully disrupts the XPO2/KPNA transport cycle. In addition, gambogic acid has recently been shown to kill stem-like colorectal cancer cells [40].

## Conclusion

Based on our data we conclude that KPNA2 is required for full stathmin expression in HCC by mediating the nuclear import of E2F1 and TFDP1. By this mechanism an important member of the nuclear transport machinery could be linked to a pivotal MT-interacting protein. Therefore, compounds interfering with the nuclear transport system may be promising candidates for future therapeutic approaches in liver cancer.

## Supplementary information

**Additional file 1: Figure S1.** HMOX1 and GTSF1 are differentially expressed upon KPNA2 depletion. HLE cells were siRNA-treated and harvested 72 h later. Lysates were immunoblotted using the indicated antibodies. **Figure S2.** KPNA2 depletion reduces the clonogenic capacity of HCC cells being phenocopied by stathmin knockdown. **a** HLE and HLF cells were treated with ctrl. or stathmin siRNAs and harvested 72 h later. Lysates were immunoblotted using the indicated antibodies. (b,c) HLF cells were treated either with ctrl. or pooled KPNA2 siRNAs (siRNAs KPNA2#1 and #2) and colony formation was analyzed 14 days after treatment by crystal violet staining (*n* = 4; *p* < 0.05 (*)). **d**,**e** HLF cells were treated with ctrl. or pooled stathmin siRNAs (siRNAs stathmin#1 and #2) and colony formation was analyzed as described in **(b)** (n = 4; p < 0.05 (*)). **Figure S3.** KPNA2 mediates nuclear import of the transcription factor c-JUN. (a) HLE cells were treated with ctrl. or KPNA2 siRNAs and nuclear-cytoplasmic fractionation was performed after 72 h. Samples were immunoblotted using the indicated antibodies. **b** KPNA2 immunoprecipitation was performed in HLE and HLF cells and samples were immunoblotted using the indicated antibodies. **c** HLE cells were treated with ctrl. siRNA or siRNAs directed against c-JUN and *STMN1* expression was analyzed by qRT-PCR (*n* = 2). **Figure S4.** KPNA2 regulates *STMN1* by import of the transcription factors E2F1 and TFDP1. **a** HLF cells were co-transfected with HA-tagged KPNA2 and Flag-tagged E2F1 or TFDP1. KPNA2 immunoprecipitation was performed and samples were immunoblotted using the indicated antibodies. **b**,**c** HLF cells were treated with ctrl. siRNA or pooled siRNAs directed against E2F1 (siRNAs E2F1#1 and #2) or TFDP1 (siRNAs TFDP1#1 and #2) and *STMN1* expression was analyzed by qRT-PCR (n = 4; p < 0.05 (*)). **d** HLF cells were treated with siRNAs directed against E2F1 and TFDP1 and *STMN1* expression was analyzed by qRT-PCR (n = 4; p < 0.05 (*)). **e** E2F1 was immunoprecipitated in HLE cells, ChIP assay was performed and precipitated DNA of the predicted *STMN1* bindings sites, the positive control binding site (*CDC2*) and a control region (neg ctrl) was quantified using qRT-PCR. The bar diagram depicts one representative experiment. **f** TFDP1 was immunoprecipitated in HLE cells and ChIP assay was performed as described in **(e)**. The bar diagram depicts one representative experiment. **Figure S5.** Stathmin is overexpressed in a murine E2F1-driven liver tumor model. **a** Micrographs show H&E staining (upper row) or Stathmin staining (lower row) of murine liver tumors that developed in an E2F1-transgenic mouse model. Scale bar: 100 μm (left panel) or 1 mm (right panel). Dashed line: tumor margins. **b** Bar diagram shows the percentage of liver tumors per mouse with mild, moderate, or strong positivity for Stathmin in E2F1-transgenic mice (*n* = 11). **Figure S6.** *KPNA2* and *STMN1* are overexpressed and correlated in human HCC. **a** Spearman correlation between *KPNA2* and *STMN1* mRNA expression in a large HCC cohort (TCGA LIHC cohort). **b** *KPNA2* and *STMN1* are strongly expressed in human HCC samples compared to adjacent non-tumorous tissues of the Roessler cohort. **c** Spearman correlation between different *KPNA*s and *STMN1* mRNA expression in the Roessler cohort. **d** Spearman correlation between *KPNA*2 and *STMN2–4* mRNA expression in the Roessler cohort. **e** Spearman correlation between the mRNA expression of *KPNA*2 and MT-associated factors in the Roessler cohort. **Figure S7.** High expression of *KPNA2* and *STMN1* correlates with poor prognosis in HCC patients. Overall survival of HCC patients showing low and high mRNA expression of *KPNA2* (a) and *STMN1* (b) or both (c) (cut-off: median, TCGA LIHC cohort). (PPTX 2860 kb)

**Additional file 2: Table S1.** Significantly differentially expressed proteins upon KPNA2-depletion. List of proteins with a log_2_ fold change of ≥ 0.8 or ≤ − 0.8 and an individual *p*-value of ≤ 0.01 that were differentially expressed following KPNA2 depletion as indicated by LC-MS/MS analysis. (DOCX 14 kb)

**Additional file 3: Table S2.** Differentially expressed proteins upon KPNA2-depletion. List of all proteins that were quantified in the LC-MS/MS approach following KPNA2 depletion. (DOCX 122 kb)

## Data Availability

A detailed list of LC-MS/MS data is provided in Additional file 2: Table S1 and Additional file 3: Table S2. E2F1 binding sites at the *STMN1* Promoter were identified using three publicly available ChIP-Seq datasets with the following Gene Expression Omnibus (GEO) numbers: GEO:GSM935484 (HeLa-S3 cells); GEO:GSM935477 (MCF-7cells) and GEO:GSM2827552 (K562 cells); accessible via Encodeproject: https://www.encodeproject.org/search/?searchTerm=e2f1 Transcriptomic data of the HCC patients cohorts are accessible via Oncomine: www.oncomine.org (Roessler cohort; GEO:GSE14520) and the Cancer Genome Atlas Program: http://cancergenome.nih.gov (TCGA LIHC cohort).

## Abbreviations

ChIP: Chromatin immunoprecipitation
CLIP1: CAP-Gly domain-containing linker protein 1
CoIP: Co-immunoprecipitation
FBP: FUSE binding protein
GTSF1: Gametocyte-specific factor 1
HBV: Hepatitis B virus
HCC: Hepatocellular carcinoma
HGF: Hepatocyte growth factor
KIF2A: Kinesin Family Member 2A
KPNA: Karyopherin-α
KPNB: Karyopherin-β
LC-MS/MS: Liquid chromatography tandem mass spectrometry
MAP 4: Microtubules associated protein 4
MT: Microtubule
NPC: Nuclear pore complex
NSCLC: Non-small cell lung carcinoma
NTR: Nuclear transport receptor
NTS: Nuclear transport system
Nup: Nucleoporin
SDCB: Syndecan binding protein (Syntenin-1)
SINE: Selective inhibitor of nuclear export
TACE: Transarterial chemoembolization
TF: Transcription factor
TPX2: Targeting protein for *Xenopus* kinesin-like protein 2
XPO: Exportin
