# Supplementary material for: Karyopherin α2-dependent import of E2F1 and TFDP1 maintains protumorigenic stathmin expression in liver cancer
Source: Cell Commun Signal. 2019 Nov 29;17:159. doi: 10.1186/s12964-019-0456-x (PMC6883611; doi:10.1186/s12964-019-0456-x)
Supplement: Supplementary file 1 — Additional file 1: Figure S1. HMOX1 and GTSF1 are differentially expressed upon KPNA2 depletion. HLE cells were siRNA-treated and harvested 72 h later. Lysates were immunoblotted using the indicated antibodies. Figure S2. KPNA2 depletion reduces the clonogenic capacity of HCC cells being phenocopied by stathmin knockdown. a HLE and HLF cells were treated with ctrl. or stathmin siRNAs and harvested 72 h later. Lysates were immunoblotted using the indicated antibodies. (b,c) HLF cells were treated either with ctrl. or pooled KPNA2 siRNAs (siRNAs KPNA2#1 and #2) and colony formation was analyzed 14 days after treatment by crystal violet staining (n = 4; p < 0.05 (*)). d,e HLF cells were treated with ctrl. or pooled stathmin siRNAs (siRNAs stathmin#1 and #2) and colony formation was analyzed as described in (b) (n = 4; p < 0.05 (*)). Figure S3. KPNA2 mediates nuclear import of the transcription factor c-JUN. (a) HLE cells were treated with ctrl. or KPNA2 siRNAs and nuclear-cytoplasmic fractionation was performed after 72 h. Samples were immunoblotted using the indicated antibodies. b KPNA2 immunoprecipitation was performed in HLE and HLF cells and samples were immunoblotted using the indicated antibodies. c HLE cells were treated with ctrl. siRNA or siRNAs directed against c-JUN and STMN1 expression was analyzed by qRT-PCR (n = 2). Figure S4. KPNA2 regulates STMN1 by import of the transcription factors E2F1 and TFDP1. a HLF cells were co-transfected with HA-tagged KPNA2 and Flag-tagged E2F1 or TFDP1. KPNA2 immunoprecipitation was performed and samples were immunoblotted using the indicated antibodies. b,c HLF cells were treated with ctrl. siRNA or pooled siRNAs directed against E2F1 (siRNAs E2F1#1 and #2) or TFDP1 (siRNAs TFDP1#1 and #2) and STMN1 expression was analyzed by qRT-PCR (n = 4; p < 0.05 (*)). d HLF cells were treated with siRNAs directed against E2F1 and TFDP1 and STMN1 expression was analyzed by qRT-PCR (n = 4; p < 0.05 (*)). e E2F1 was immunoprecipitated i [file 12964_2019_456_MOESM1_ESM.pptx]

## Slide 1
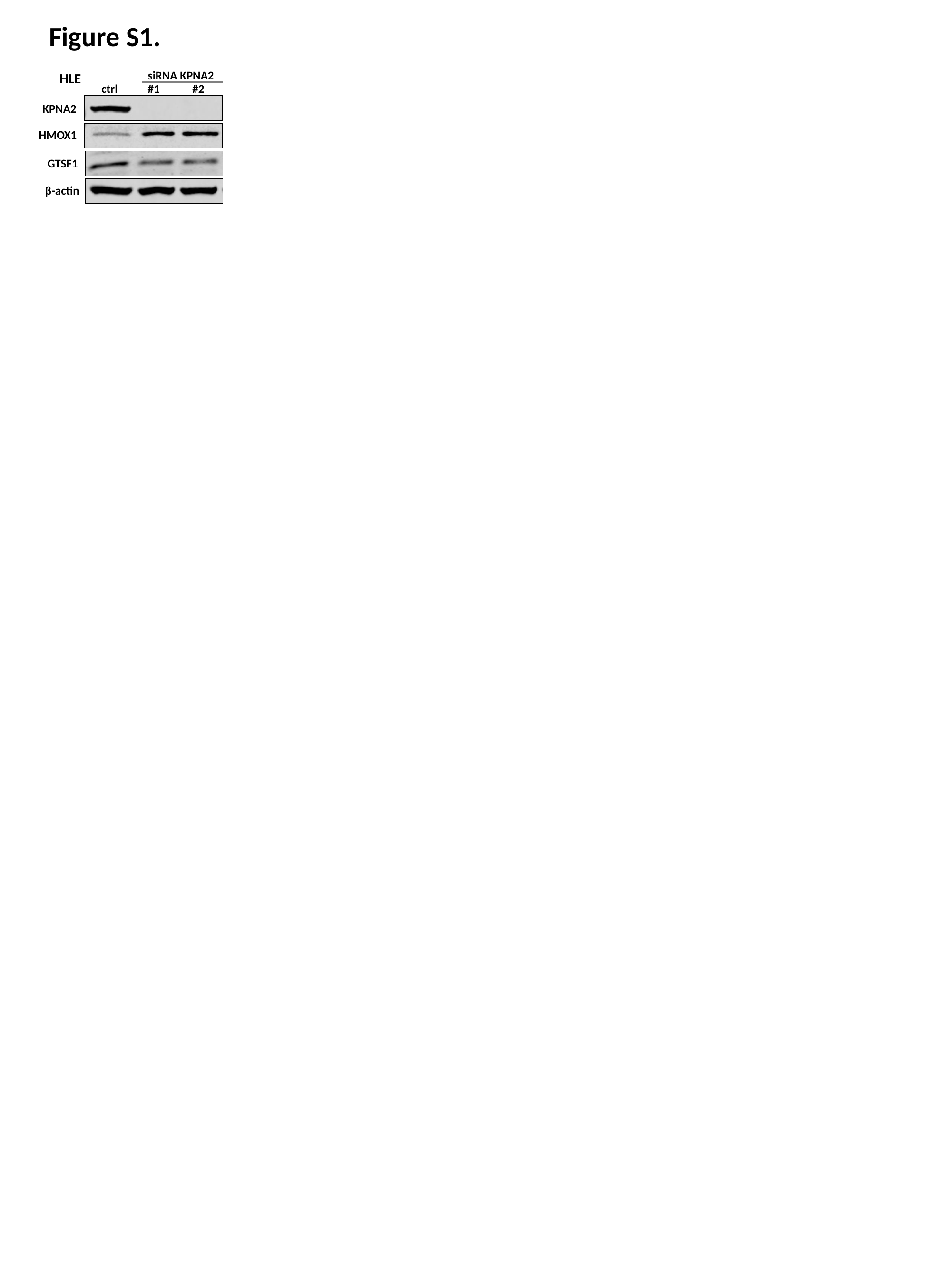

Figure S1.
siRNA KPNA2
HLE
 ctrl #1 #2
KPNA2
HMOX1
GTSF1
β-actin

## Slide 2
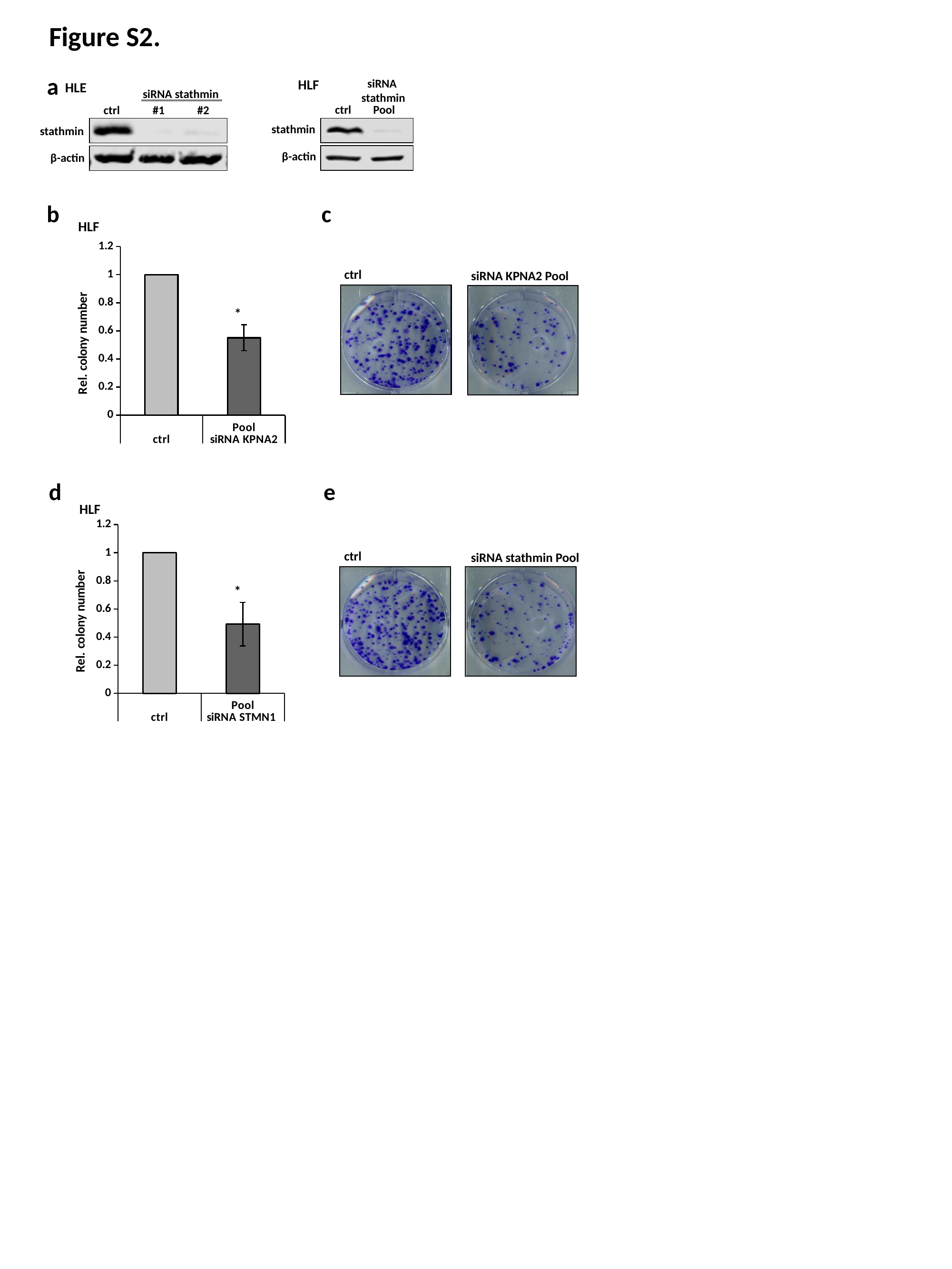

Figure S2.
a
siRNA
stathmin
HLF
HLE
siRNA stathmin
 ctrl Pool
 ctrl #1 #2
stathmin
stathmin
β-actin
β-actin
c
b
HLF
### Chart
| Category | rel. Colonies |
|---|---|
| | 1.0 |
| Pool | 0.5503964513035637 |ctrl
siRNA KPNA2 Pool
*
e
d
HLF
### Chart
| Category | rel. Colonies |
|---|---|
| | 1.0 |
| Pool | 0.49188804930966357 |ctrl
siRNA stathmin Pool
*

## Slide 3
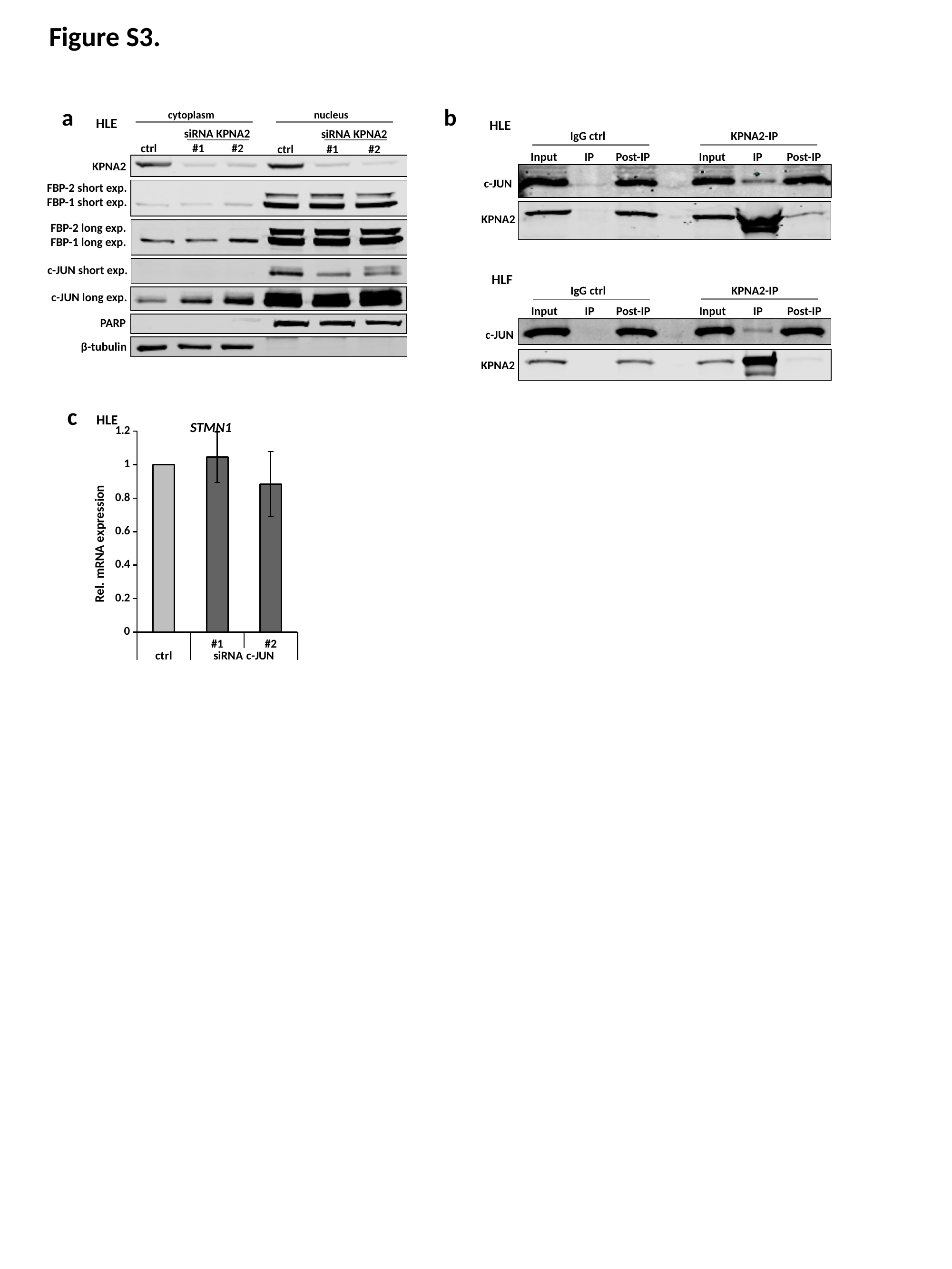

Figure S3.
a
b
 cytoplasm
 nucleus
HLE
HLE
siRNA KPNA2
siRNA KPNA2
IgG ctrl KPNA2-IP
 ctrl #1 #2
 ctrl #1 #2
 Input IP Post-IP Input IP Post-IP
KPNA2
c-JUN
FBP-2 short exp.
FBP-1 short exp.
KPNA2
FBP-2 long exp.
FBP-1 long exp.
c-JUN short exp.
HLF
IgG ctrl KPNA2-IP
c-JUN long exp.
 Input IP Post-IP Input IP Post-IP
PARP
c-JUN
β-tubulin
KPNA2
c
HLE
STMN1
### Chart
| Category | STMN1 |
|---|---|
| | 1.0000030260728323 |
| #1 | 1.0447299235478202 |
| #2 | 0.8832162428145948 |

## Slide 4
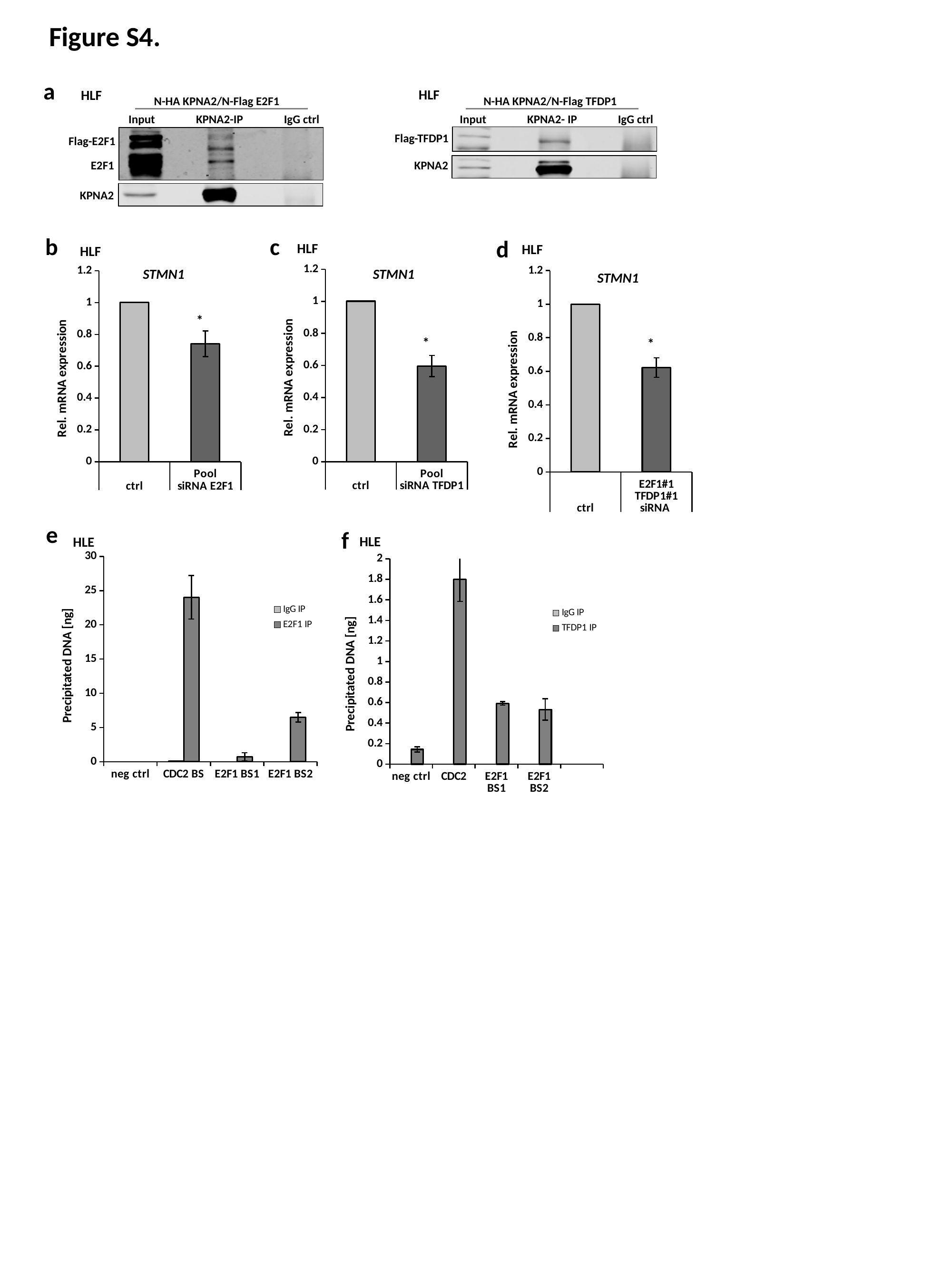

Figure S4.
a
HLF
HLF
 N-HA KPNA2/N-Flag E2F1
 N-HA KPNA2/N-Flag TFDP1
 Input KPNA2-IP IgG ctrl
 Input KPNA2- IP IgG ctrl
Flag-TFDP1
Flag-E2F1
E2F1
KPNA2
KPNA2
b
c
d
HLF
HLF
HLF
### Chart
| Category | STMN1 |
|---|---|
| | 1.0010093398073803 |
| Pool | 0.5970795180999796 |
### Chart
| Category | STMN1 |
|---|---|
| | 1.000096881520915 |
| E2F1#1 TFDP1#1 | 0.6219453214615227 |
### Chart
| Category | STMN1 |
|---|---|
| | 1.0009707467346236 |
| Pool | 0.7416981292265014 |STMN1
STMN1
STMN1
*
*
*
e
f
HLE
HLE
### Chart
| Category | IgG IP | E2F1 IP |
|---|---|---|
| neg ctrl | None | None |
| CDC2 BS | 0.09378673265018624 | 24.021109005088153 |
| E2F1 BS1 | None | 0.7303843871392923 |
| E2F1 BS2 | None | 6.497155709456396 |
### Chart
| Category | IgG IP | TFDP1 IP |
|---|---|---|
| neg ctrl | 0.03718163573904629 | 0.1440957757269491 |
| CDC2 | 0.016564590467678993 | 1.8006860237564466 |
| E2F1 BS1 | 0.03311877211096313 | 0.5909974368530047 |
| E2F1 BS2 | 0.01690141592549226 | 0.5325892646114718 |

## Slide 5
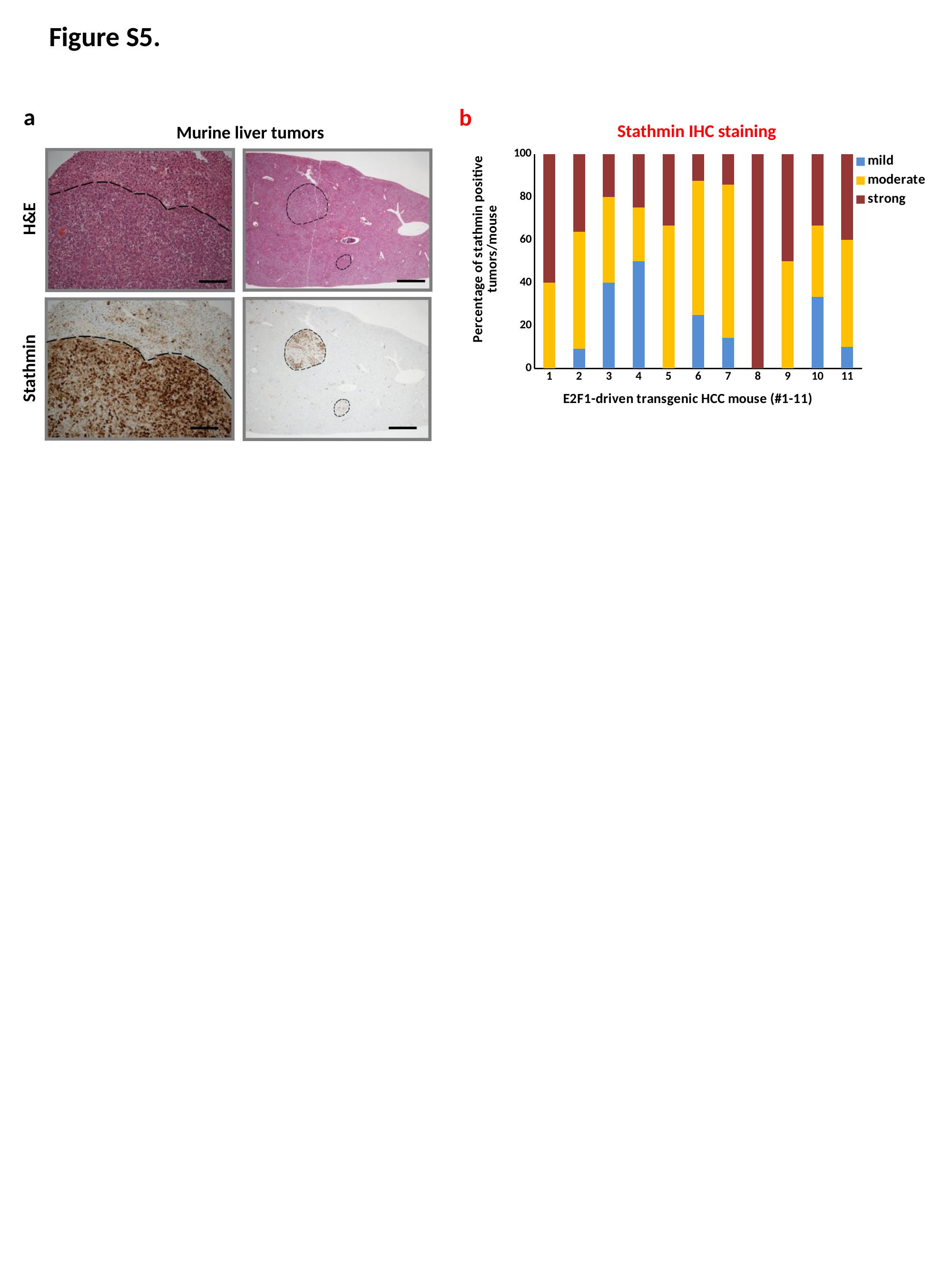

Figure S5.
a
b
Stathmin IHC staining
Murine liver tumors
### Chart
| Category | mild | moderate | strong |
|---|---|---|---|
| 1 | 0.0 | 40.0 | 60.0 |
| 2 | 9.090909090909092 | 54.54545454545454 | 36.36363636363637 |
| 3 | 40.0 | 40.0 | 20.0 |
| 4 | 50.0 | 25.0 | 25.0 |
| 5 | 0.0 | 66.66666666666666 | 33.33333333333333 |
| 6 | 25.0 | 62.5 | 12.5 |
| 7 | 14.285714285714285 | 71.42857142857143 | 14.285714285714285 |
| 8 | 0.0 | 0.0 | 100.0 |
| 9 | 0.0 | 50.0 | 50.0 |
| 10 | 33.33333333333333 | 33.33333333333333 | 33.33333333333333 |
| 11 | 10.0 | 50.0 | 40.0 |
H&E
Stathmin

## Slide 6
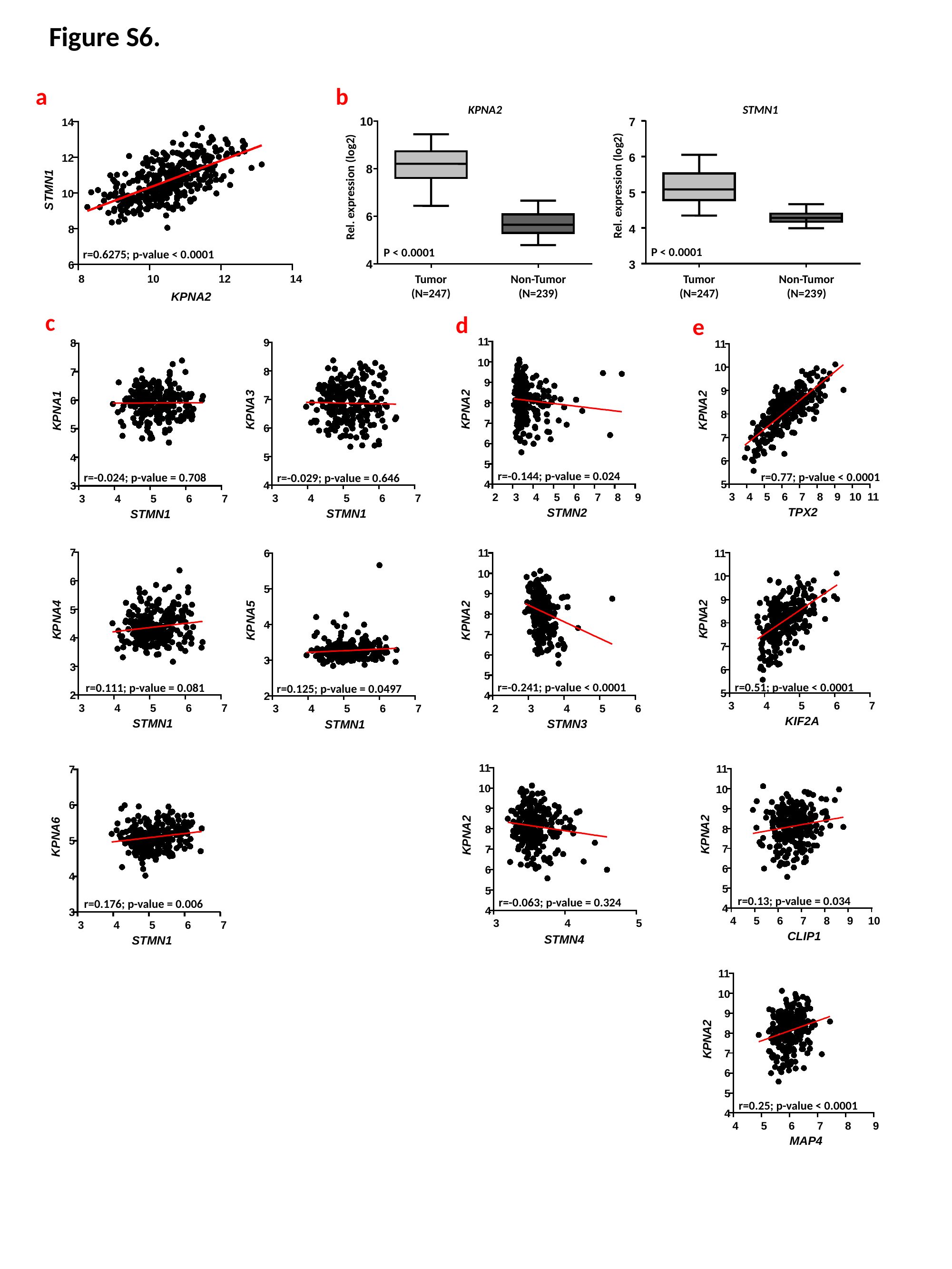

Figure S6.
a
b
KPNA2
STMN1
10
7
14
12
10
8
6
8
10
12
14
1
N
M
T
S
 KPNA2
6
8
 Rel. expression (log2)
 Rel. expression (log2)
5
6
4
P < 0.0001
P < 0.0001
r=0.6275; p-value < 0.0001
4
3
Tumor
(N=247)
Non-Tumor
(N=239)
Tumor
(N=247)
Non-Tumor
(N=239)
c
d
e
11
10
9
8
7
6
5
4
2
3
4
5
6
7
8
9
9
8
7
6
5
4
3
4
5
6
7
8
7
6
5
4
3
3
4
5
6
7
11
10
9
8
7
6
5
3
4
5
6
7
8
9
10
11
2
3
2
1
A
A
A
A
N
N
N
N
P
P
P
P
K
K
K
K
r=-0.144; p-value = 0.024
r=0.77; p-value < 0.0001
r=-0.024; p-value = 0.708
r=-0.029; p-value = 0.646
TPX2
STMN2
STMN1
STMN1
7
6
5
4
3
2
3
4
5
6
7
11
10
9
8
7
6
5
4
2
3
4
5
6
6
11
10
9
8
7
6
5
3
4
5
6
7
5
2
4
2
5
A
A
A
A
N
N
N
N
4
P
P
P
P
K
K
K
K
3
r=0.51; p-value < 0.0001
 r=-0.241; p-value < 0.0001
r=0.111; p-value = 0.081
r=0.125; p-value = 0.0497
2
3
4
5
6
7
KIF2A
STMN1
STMN3
STMN1
11
10
9
8
7
6
5
4
3
4
5
11
10
9
8
7
6
5
4
4
5
6
7
8
9
10
7
6
5
4
3
3
4
5
6
7
2
2
6
A
A
A
N
N
N
P
P
P
K
K
K
r=0.13; p-value = 0.034
r=-0.063; p-value = 0.324
r=0.176; p-value = 0.006
CLIP1
STMN4
STMN1
11
10
9
8
7
6
5
4
4
5
6
7
8
9
2
A
N
P
K
r=0.25; p-value < 0.0001
MAP4

## Slide 7
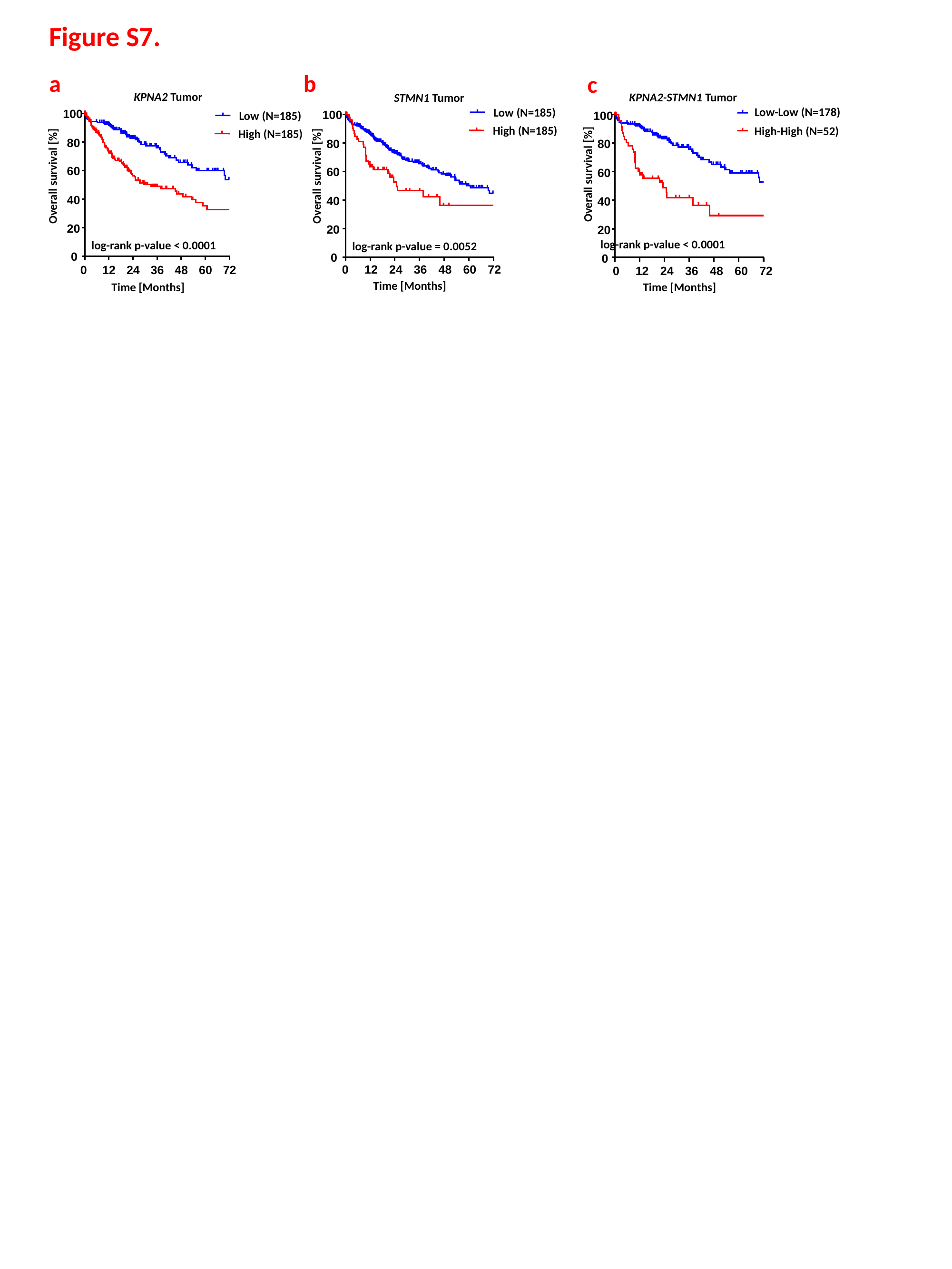

Figure S7.
a
b
c
KPNA2 Tumor
KPNA2-STMN1 Tumor
STMN1 Tumor
Low-Low (N=178)
Low (N=185)
100
80
60
40
20
0
0
12
24
36
48
60
72
100
80
60
40
20
0
0
12
24
36
48
60
72
100
Low (N=185)
High (N=185)
High-High (N=52)
High (N=185)
80
60
Overall survival [%]
Overall survival [%]
Overall survival [%]
40
20
log-rank p-value < 0.0001
log-rank p-value < 0.0001
log-rank p-value = 0.0052
0
0
12
24
36
48
60
72
Time [Months]
Time [Months]
Time [Months]
