# Supplementary material for: Karyopherin α2-dependent import of E2F1 and TFDP1 maintains protumorigenic stathmin expression in liver cancer
Source: Cell Commun Signal. 2019 Nov 29;17:159. doi: 10.1186/s12964-019-0456-x (PMC6883611; doi:10.1186/s12964-019-0456-x)
Supplement: Supplementary file 2 — Additional file 2: Table S1. Significantly differentially expressed proteins upon KPNA2-depletion. List of proteins with a log2 fold change of ≥ 0.8 or ≤ − 0.8 and an individual p-value of ≤ 0.01 that were differentially expressed following KPNA2 depletion as indicated by LC-MS/MS analysis. (DOCX 14 kb) [file 12964_2019_456_MOESM2_ESM.docx]

**Table S1.** **Significantly differentially expressed proteins upon KPNA2-depletion.**

HLE cells were harvested 72 h after control or KPNA2 siRNA treatment (n=3) and analyzed using LC-MS/MS. Proteins with a log_2_ fold change of ≥0.8 or ≤ -0.8 and an individual p-value of ≤0.01 are listed. KPNA2 and stathmin (STMN1) are highlighted. Per protein the Uniprot ID (first column), the log_2_ fold change (second column), the individual p-value (third column), the adjusted p-value (adj. p-value, fourth column), and the short name (last column) is indicated.

| **ID** | **fold change [log_2_]** | **p-value** | **adj. p-value** | **short name** |
| --- | --- | --- | --- | --- |
| **P52292** | **-4,06652799** | **2,22E-08** | **3,91E-05** | **KPNA2_HUMAN** |
| Q8WW33 | -1,469968789 | 0,00435593 | 0,16302233 | GTSF1_HUMAN |
| O00560 | -1,390320354 | 0,00016865 | 0,0707303 | SDCB1_HUMAN |
| P20700 | -1,31021712 | 5,52E-05 | 0,04182953 | LMNB1_HUMAN |
| P28074 | -1,166164952 | 0,00298521 | 0,15727965 | PSB5_HUMAN |
| Q9Y4Z0 | -1,008477528 | 0,00051366 | 0,08032323 | LSM4_HUMAN |
| Q10471 | -1,007840338 | 0,00046061 | 0,08032323 | GALT2_HUMAN |
| P30154 | -0,957951503 | 0,00061188 | 0,08274524 | 2AAB_HUMAN |
| **P16949** | **-0,924246545** | **0,00347536** | **0,15727965** | **STMN1_HUMAN** |
| Q9ULW0 | 0,803453446 | 7,14E-05 | 0,04182953 | TPX2_HUMAN |
| P21980 | 0,806458348 | 0,00051554 | 0,08032323 | TGM2_HUMAN |
| P53814 | 0,840950498 | 0,00527634 | 0,18036487 | SMTN_HUMAN |
| O14907 | 0,84958316 | 0,00370485 | 0,16085146 | TX1B3_HUMAN |
| Q9BS26 | 0,971714852 | 0,00435839 | 0,16302233 | ERP44_HUMAN |
| Q9UKA9 | 0,999421233 | 0,0045918 | 0,16817467 | PTBP2_HUMAN |
