# Supplementary material for: Karyopherin α2-dependent import of E2F1 and TFDP1 maintains protumorigenic stathmin expression in liver cancer
Source: Cell Commun Signal. 2019 Nov 29;17:159. doi: 10.1186/s12964-019-0456-x (PMC6883611; doi:10.1186/s12964-019-0456-x)
Supplement: Supplementary file 3 — Additional file 3: Table S2. Differentially expressed proteins upon KPNA2-depletion. List of all proteins that were quantified in the LC-MS/MS approach following KPNA2 depletion. (DOCX 122 kb) [file 12964_2019_456_MOESM3_ESM.docx]

**Table S2.** Title: Differentially expressed proteins upon KPNA2-depletion.

HLE cells were harvested 72 h after control or KPNA2 siRNA treatment (n=3) and analyzed using LC-MS/MS. Per protein the Uniprot ID (first column), the log_2_ fold change (second column), the individual p-value (third column), the adjusted p-value (adj. p-value, fourth column), and the short name (last column) is indicated. KPNA2 and stathmin (STMN1) are highlighted.

| **ID** | **fold change [log_2_]** | **p-value** | **adj. p-value** | **short name** |
| --- | --- | --- | --- | --- |
| **P52292** | **-4,06652799** | **2,22E-08** | **3,91E-05** | **KPNA2_HUMAN** |
| Q13247 | -2,086579987 | 0,01177021 | 0,21331985 | SRSF6_HUMAN |
| Q8WW33 | -1,469968789 | 0,00435593 | 0,16302233 | GTSF1_HUMAN |
| O00560 | -1,390320354 | 0,00016865 | 0,0707303 | SDCB1_HUMAN |
| P20700 | -1,31021712 | 5,52E-05 | 0,04182953 | LMNB1_HUMAN |
| P28074 | -1,166164952 | 0,00298521 | 0,15727965 | PSB5_HUMAN |
| P62314 | -1,094443804 | 0,05202138 | 0,36435692 | SMD1_HUMAN |
| Q9Y4E8 | -1,062159153 | 0,03505649 | 0,33313144 | UBP15_HUMAN |
| Q9Y4Z0 | -1,008477528 | 0,00051366 | 0,08032323 | LSM4_HUMAN |
| Q10471 | -1,007840338 | 0,00046061 | 0,08032323 | GALT2_HUMAN |
| P30154 | -0,957951503 | 0,00061188 | 0,08274524 | 2AAB_HUMAN |
| **P16949** | **-0,924246545** | **0,00347536** | **0,15727965** | **STMN1_HUMAN** |
| P29590 | -0,918142433 | 0,04884305 | 0,36117836 | PML_HUMAN |
| P20618 | -0,863252271 | 0,08238103 | 0,42518835 | PSB1_HUMAN |
| Q99436 | -0,809284713 | 0,09842096 | 0,44565474 | PSB7_HUMAN |
| P62847 | -0,795179066 | 0,13272856 | 0,50303967 | RS24_HUMAN |
| Q9UMY4 | -0,79109985 | 0,01681844 | 0,26165324 | SNX12_HUMAN |
| Q9Y508 | -0,785957085 | 0,0054811 | 0,18051136 | RN114_HUMAN |
| Q15269 | -0,783813019 | 0,0207403 | 0,28120308 | PWP2_HUMAN |
| Q13509 | -0,767898445 | 0,03317723 | 0,32693324 | TBB3_HUMAN |
| Q53GQ0 | -0,761867846 | 0,04233141 | 0,34514615 | DHB12_HUMAN |
| P49589 | -0,757038963 | 0,04747328 | 0,35973287 | SYCC_HUMAN |
| O94905 | -0,751309351 | 0,00585276 | 0,18051136 | ERLN2_HUMAN |
| P46783 | -0,745375832 | 0,02355126 | 0,29019833 | RS10_HUMAN |
| Q13620 | -0,741820141 | 0,01586513 | 0,25355364 | CUL4B_HUMAN |
| Q96PD2 | -0,738805068 | 0,05990561 | 0,38735155 | DCBD2_HUMAN |
| P17858 | -0,726751287 | 0,0085131 | 0,19754389 | PFKAL_HUMAN |
| P14174 | -0,721575039 | 0,08319954 | 0,42518835 | MIF_HUMAN |
| Q9UHD1 | -0,712569683 | 0,00149226 | 0,12492383 | CHRD1_HUMAN |
| Q9Y617 | -0,695984681 | 0,00020117 | 0,0707303 | SERC_HUMAN |
| P48449 | -0,692810446 | 0,02387647 | 0,29149185 | ERG7_HUMAN |
| Q13404 | -0,688605589 | 0,00045027 | 0,08032323 | UB2V1_HUMAN |
| P16401 | -0,687965271 | 0,05514733 | 0,37531168 | H15_HUMAN |
| Q9BVP2 | -0,681372826 | 0,03195762 | 0,32121512 | GNL3_HUMAN |
| P22392 | -0,67443197 | 0,00707494 | 0,1863338 | NDKB_HUMAN |
| Q99747 | -0,669091897 | 0,00674814 | 0,1863338 | SNAG_HUMAN |
| Q8NBX0 | -0,665542801 | 0,03852509 | 0,34224384 | SCPDL_HUMAN |
| P61353 | -0,662851537 | 0,08741872 | 0,43536006 | RL27_HUMAN |
| A0AVT1 | -0,643673102 | 0,00807766 | 0,19722964 | UBA6_HUMAN |
| P18206 | -0,635823717 | 0,00049619 | 0,08032323 | VINC_HUMAN |
| P04040 | -0,631849755 | 0,00084978 | 0,09959379 | CATA_HUMAN |
| Q16270 | -0,624843012 | 0,00973483 | 0,20305363 | IBP7_HUMAN |
| Q9HC35 | -0,615854415 | 0,00533502 | 0,18036487 | EMAL4_HUMAN |
| O14818 | -0,614063498 | 0,00375137 | 0,16085146 | PSA7_HUMAN |
| Q9BTW9 | -0,601594013 | 0,30824131 | 0,67635742 | TBCD_HUMAN |
| Q99439 | -0,601000292 | 0,04389248 | 0,34758102 | CNN2_HUMAN |
| P78346 | -0,598234103 | 0,11099351 | 0,46569592 | RPP30_HUMAN |
| P25786 | -0,593980928 | 0,01671678 | 0,26165324 | PSA1_HUMAN |
| P67936 | -0,593209076 | 0,00385954 | 0,16154938 | TPM4_HUMAN |
| Q9Y2P8 | -0,584285234 | 0,01400565 | 0,24139154 | RCL1_HUMAN |
| Q9Y5K6 | -0,583403064 | 0,01572939 | 0,25355364 | CD2AP_HUMAN |
| P60900 | -0,583205635 | 0,01097911 | 0,20890123 | PSA6_HUMAN |
| Q9NX46 | -0,581520475 | 0,02060122 | 0,28120308 | ARHL2_HUMAN |
| O43681 | -0,57889687 | 0,01968248 | 0,2790468 | ASNA_HUMAN |
| Q9BX68 | -0,570402703 | 0,02253281 | 0,28731937 | HINT2_HUMAN |
| Q6DD88 | -0,567257141 | 0,00627014 | 0,18371518 | ATLA3_HUMAN |
| Q03001 | -0,565920841 | 0,00694986 | 0,1863338 | DYST_HUMAN |
| Q9BW60 | -0,564580264 | 0,02333416 | 0,29019833 | ELOV1_HUMAN |
| P52926 | -0,563736583 | 0,15385297 | 0,5201414 | HMGA2_HUMAN |
| Q8IVL5 | -0,562895305 | 0,04142686 | 0,34514615 | P3H2_HUMAN |
| Q9UIQ6 | -0,560990886 | 0,00169005 | 0,12917864 | LCAP_HUMAN |
| Q9UL46 | -0,560859116 | 0,00341717 | 0,15727965 | PSME2_HUMAN |
| Q8TC12 | -0,56060372 | 0,08135519 | 0,42518835 | RDH11_HUMAN |
| P06703 | -0,558025942 | 0,00917415 | 0,20006487 | S10A6_HUMAN |
| P52943 | -0,553805813 | 0,02196804 | 0,28544594 | CRIP2_HUMAN |
| Q16625 | -0,552054489 | 0,32276355 | 0,69366542 | OCLN_HUMAN |
| P40261 | -0,544592047 | 0,04593027 | 0,35511786 | NNMT_HUMAN |
| Q9H3H3 | -0,544265811 | 0,01331184 | 0,23170507 | CK068_HUMAN |
| Q96BP3 | -0,53502041 | 0,03139173 | 0,32121512 | PPWD1_HUMAN |
| Q8TF42 | -0,533983123 | 0,00082777 | 0,09959379 | UBS3B_HUMAN |
| Q92575 | -0,533497581 | 0,14570827 | 0,51236505 | UBXN4_HUMAN |
| P19367 | -0,530052008 | 0,0061221 | 0,18349405 | HXK1_HUMAN |
| P49207 | -0,526379928 | 0,01113571 | 0,20890123 | RL34_HUMAN |
| P24941 | -0,526035479 | 0,14045814 | 0,50830271 | CDK2_HUMAN |
| P20674 | -0,524836912 | 0,22526505 | 0,60775412 | COX5A_HUMAN |
| P62330 | -0,506005889 | 0,21944612 | 0,60302172 | ARF6_HUMAN |
| Q9Y547 | -0,49345092 | 0,06681609 | 0,40114205 | IFT25_HUMAN |
| O60678 | -0,492879968 | 0,02493679 | 0,29518733 | ANM3_HUMAN |
| Q07812 | -0,491512479 | 0,26188232 | 0,6367761 | BAX_HUMAN |
| O43865 | -0,483971254 | 0,00584066 | 0,18051136 | SAHH2_HUMAN |
| Q9NZ32 | -0,483345362 | 0,03723521 | 0,33999874 | ARP10_HUMAN |
| P35221 | -0,480656299 | 0,00218211 | 0,15531532 | CTNA1_HUMAN |
| P53582 | -0,478288809 | 0,00921801 | 0,20006487 | MAP11_HUMAN |
| Q99848 | -0,477692068 | 0,20879082 | 0,59202301 | EBP2_HUMAN |
| O00244 | -0,477446474 | 0,07074286 | 0,40869439 | ATOX1_HUMAN |
| P49770 | -0,468223383 | 0,02063098 | 0,28120308 | EI2BB_HUMAN |
| Q96IJ6 | -0,465449314 | 0,12354515 | 0,48697843 | GMPPA_HUMAN |
| P55795 | -0,464878444 | 0,15052057 | 0,51715988 | HNRH2_HUMAN |
| Q9HCU5 | -0,464370527 | 0,01899611 | 0,27373079 | PREB_HUMAN |
| Q9NZL9 | -0,464062515 | 0,00337277 | 0,15727965 | MAT2B_HUMAN |
| P62253 | -0,46251605 | 0,02461054 | 0,29518733 | UB2G1_HUMAN |
| P35637 | -0,461347633 | 0,28806501 | 0,66033859 | FUS_HUMAN |
| Q12929 | -0,460466852 | 0,04081756 | 0,34498692 | EPS8_HUMAN |
| P23919 | -0,460369043 | 0,03392084 | 0,32946319 | KTHY_HUMAN |
| P34896 | -0,457332601 | 0,1440654 | 0,51231408 | GLYC_HUMAN |
| P56545 | -0,455568955 | 0,34636686 | 0,70756912 | CTBP2_HUMAN |
| Q9NZ08 | -0,453219401 | 0,04285174 | 0,34514615 | ERAP1_HUMAN |
| P26583 | -0,452393505 | 0,06946104 | 0,40869439 | HMGB2_HUMAN |
| P07355 | -0,451681437 | 0,29073836 | 0,66255294 | ANXA2_HUMAN |
| Q8N6T3 | -0,451283279 | 0,04265226 | 0,34514615 | ARFG1_HUMAN |
| Q4VC31 | -0,44595863 | 0,22253002 | 0,60546408 | CCD58_HUMAN |
| P48739 | -0,443590899 | 0,21582068 | 0,59897319 | PIPNB_HUMAN |
| P08397 | -0,442878952 | 0,01294495 | 0,22757224 | HEM3_HUMAN |
| O15294 | -0,439891922 | 0,05320129 | 0,36821993 | OGT1_HUMAN |
| Q8IWS0 | -0,439671462 | 0,07853018 | 0,42518835 | PHF6_HUMAN |
| P30530 | -0,439354303 | 0,01616367 | 0,2559976 | UFO_HUMAN |
| P51570 | -0,435641753 | 0,00992982 | 0,20305363 | GALK1_HUMAN |
| Q9Y2Z0 | -0,434860436 | 0,04995819 | 0,36167674 | SUGT1_HUMAN |
| P08574 | -0,43420386 | 0,06685701 | 0,40114205 | CY1_HUMAN |
| Q96C01 | -0,43347208 | 0,21635266 | 0,59897319 | F136A_HUMAN |
| O95155 | -0,433080915 | 0,00265285 | 0,15727965 | UBE4B_HUMAN |
| Q8TDB4 | -0,432719072 | 0,01164507 | 0,2132503 | HUMMR_HUMAN |
| Q53H82 | -0,430127719 | 0,07762071 | 0,42518835 | LACB2_HUMAN |
| P51571 | -0,425704504 | 0,08661185 | 0,43536006 | SSRD_HUMAN |
| O00743 | -0,422809932 | 0,09284498 | 0,44565474 | PPP6_HUMAN |
| P15559 | -0,420014373 | 0,09633086 | 0,44565474 | NQO1_HUMAN |
| Q00688 | -0,419191785 | 0,09506452 | 0,44565474 | FKBP3_HUMAN |
| P51114 | -0,414165952 | 0,09886539 | 0,44565474 | FXR1_HUMAN |
| P80217 | -0,413906348 | 0,05105237 | 0,36336059 | IN35_HUMAN |
| Q15907 | -0,413809483 | 0,03088485 | 0,32121512 | RB11B_HUMAN |
| Q96H79 | -0,412574647 | 0,02904908 | 0,31330234 | ZCCHL_HUMAN |
| P36405 | -0,40945482 | 0,22611013 | 0,60775412 | ARL3_HUMAN |
| Q8N8S7 | -0,408112661 | 0,09164413 | 0,44383025 | ENAH_HUMAN |
| P40937 | -0,407850169 | 0,01487287 | 0,25000899 | RFC5_HUMAN |
| Q13043 | -0,397026604 | 0,18727056 | 0,57348817 | STK4_HUMAN |
| P25789 | -0,394484492 | 0,02208228 | 0,28544594 | PSA4_HUMAN |
| Q3KQU3 | -0,390741934 | 0,13554123 | 0,50376636 | MA7D1_HUMAN |
| Q04206 | -0,390384682 | 0,03872182 | 0,34224384 | TF65_HUMAN |
| Q8N1G4 | -0,384342043 | 0,01127648 | 0,20890123 | LRC47_HUMAN |
| P46781 | -0,384257633 | 0,11783845 | 0,47622986 | RS9_HUMAN |
| P50914 | -0,380402679 | 0,00399078 | 0,16290903 | RL14_HUMAN |
| Q8NI36 | -0,377492635 | 0,00729264 | 0,1885363 | WDR36_HUMAN |
| P13489 | -0,375096472 | 0,0096247 | 0,20305363 | RINI_HUMAN |
| Q02809 | -0,372892783 | 0,09641072 | 0,44565474 | PLOD1_HUMAN |
| P59998 | -0,372531951 | 0,06208313 | 0,39544254 | ARPC4_HUMAN |
| P61619 | -0,368931614 | 0,18960694 | 0,57594082 | S61A1_HUMAN |
| O43447 | -0,368708765 | 0,09147612 | 0,44383025 | PPIH_HUMAN |
| P13693 | -0,368018946 | 0,10257672 | 0,45002029 | TCTP_HUMAN |
| P50454 | -0,363609341 | 0,01128875 | 0,20890123 | SERPH_HUMAN |
| P43034 | -0,363224446 | 0,00665418 | 0,1863338 | LIS1_HUMAN |
| P35613 | -0,362229051 | 0,13529466 | 0,50376636 | BASI_HUMAN |
| Q9UNS2 | -0,361024621 | 0,00850975 | 0,19754389 | CSN3_HUMAN |
| P06396 | -0,359045465 | 0,02320473 | 0,29019833 | GELS_HUMAN |
| P15170 | -0,359002281 | 0,33379578 | 0,69858689 | ERF3A_HUMAN |
| Q16401 | -0,356733082 | 0,14419158 | 0,51231408 | PSMD5_HUMAN |
| P35249 | -0,356087367 | 0,14797342 | 0,51293778 | RFC4_HUMAN |
| Q13907 | -0,354599202 | 0,04990234 | 0,36167674 | IDI1_HUMAN |
| P52306 | -0,351737609 | 0,29395501 | 0,66337984 | GDS1_HUMAN |
| P08708 | -0,35054338 | 0,00792504 | 0,19622837 | RS17_HUMAN |
| Q03426 | -0,349739538 | 0,07989414 | 0,42518835 | KIME_HUMAN |
| Q8WU90 | -0,348595007 | 0,27456738 | 0,65033117 | ZC3HF_HUMAN |
| P68400 | -0,345679475 | 0,07275616 | 0,41163083 | CSK21_HUMAN |
| Q00765 | -0,344911127 | 0,25754144 | 0,63432137 | REEP5_HUMAN |
| Q9NR12 | -0,342361027 | 0,02204567 | 0,28544594 | PDLI7_HUMAN |
| Q01469 | -0,334714076 | 0,49863705 | 0,79836423 | FABP5_HUMAN |
| O94925 | -0,333738862 | 0,01532857 | 0,25000899 | GLSK_HUMAN |
| Q8IVF2 | -0,333109027 | 0,01079695 | 0,20890123 | AHNK2_HUMAN |
| Q9Y281 | -0,328975651 | 0,22297665 | 0,60546408 | COF2_HUMAN |
| P04792 | -0,327199608 | 0,04081342 | 0,34498692 | HSPB1_HUMAN |
| Q8NBJ5 | -0,324167242 | 0,15705421 | 0,52149753 | GT251_HUMAN |
| P18077 | -0,324134296 | 0,10902168 | 0,46569592 | RL35A_HUMAN |
| P40222 | -0,323825233 | 0,0271643 | 0,30809576 | TXLNA_HUMAN |
| Q96QK1 | -0,314062363 | 0,01752895 | 0,2659057 | VPS35_HUMAN |
| Q14738 | -0,314024506 | 0,22642029 | 0,60775412 | 2A5D_HUMAN |
| Q9H936 | -0,311999935 | 0,03663116 | 0,33893459 | GHC1_HUMAN |
| P51398 | -0,309529081 | 0,46338394 | 0,77583711 | RT29_HUMAN |
| Q9UKD2 | -0,307952326 | 0,42773065 | 0,76056043 | MRT4_HUMAN |
| Q15404 | -0,307929309 | 0,05225864 | 0,3645662 | RSU1_HUMAN |
| Q8TCJ2 | -0,306539983 | 0,06635549 | 0,40114205 | STT3B_HUMAN |
| P08133 | -0,30451433 | 0,42014708 | 0,75742583 | ANXA6_HUMAN |
| P25788 | -0,304017273 | 0,26929573 | 0,64270609 | PSA3_HUMAN |
| O43852 | -0,297561408 | 0,02783707 | 0,30973139 | CALU_HUMAN |
| Q14444 | -0,297313082 | 0,02058215 | 0,28120308 | CAPR1_HUMAN |
| P38606 | -0,29535766 | 0,07069007 | 0,40869439 | VATA_HUMAN |
| Q14141 | -0,291983398 | 0,07281979 | 0,41163083 | SEPT6_HUMAN |
| P53985 | -0,291811459 | 0,20771994 | 0,59089263 | MOT1_HUMAN |
| P54619 | -0,2903703 | 0,04649956 | 0,35697045 | AAKG1_HUMAN |
| P53701 | -0,289953673 | 0,13226246 | 0,50303967 | CCHL_HUMAN |
| Q14558 | -0,288753882 | 0,11928525 | 0,47991871 | KPRA_HUMAN |
| P08758 | -0,285813112 | 0,02621333 | 0,29924046 | ANXA5_HUMAN |
| P23284 | -0,28564963 | 0,04282424 | 0,34514615 | PPIB_HUMAN |
| O95865 | -0,284918267 | 0,09748907 | 0,44565474 | DDAH2_HUMAN |
| P14678 | -0,284505599 | 0,14739385 | 0,51293778 | RSMB_HUMAN |
| Q9Y6G9 | -0,282185812 | 0,03356853 | 0,32785268 | DC1L1_HUMAN |
| Q92538 | -0,282114632 | 0,01529278 | 0,25000899 | GBF1_HUMAN |
| P04899 | -0,28060497 | 0,01780653 | 0,2659057 | GNAI2_HUMAN |
| Q99598 | -0,277714363 | 0,15193004 | 0,51862722 | TSNAX_HUMAN |
| O95373 | -0,276350452 | 0,20123258 | 0,5839878 | IPO7_HUMAN |
| Q9Y230 | -0,27627847 | 0,13420114 | 0,50303967 | RUVB2_HUMAN |
| O95163 | -0,275300264 | 0,1431604 | 0,51231408 | ELP1_HUMAN |
| P21291 | -0,274800138 | 0,29171811 | 0,66255294 | CSRP1_HUMAN |
| P43490 | -0,273574139 | 0,22816708 | 0,60775412 | NAMPT_HUMAN |
| P17174 | -0,272830543 | 0,23603891 | 0,61841491 | AATC_HUMAN |
| Q5JTH9 | -0,272740535 | 0,03066559 | 0,32121512 | RRP12_HUMAN |
| Q8WVM8 | -0,271959688 | 0,08244442 | 0,42518835 | SCFD1_HUMAN |
| P09110 | -0,271610189 | 0,15722053 | 0,52149753 | THIK_HUMAN |
| O43617 | -0,270801655 | 0,16175708 | 0,52667503 | TPPC3_HUMAN |
| P14550 | -0,270479739 | 0,13352448 | 0,50303967 | AK1A1_HUMAN |
| P39023 | -0,270433576 | 0,1601063 | 0,52317263 | RL3_HUMAN |
| Q969G5 | -0,266299673 | 0,04299602 | 0,34514615 | PRDBP_HUMAN |
| P13674 | -0,265107111 | 0,08010816 | 0,42518835 | P4HA1_HUMAN |
| Q6NZI2 | -0,265022285 | 0,07968979 | 0,42518835 | PTRF_HUMAN |
| Q6PGP7 | -0,263920294 | 0,11294009 | 0,46827519 | TTC37_HUMAN |
| P63208 | -0,263713263 | 0,25102545 | 0,62782391 | SKP1_HUMAN |
| Q13838 | -0,26334475 | 0,02995088 | 0,31911301 | DX39B_HUMAN |
| Q9Y679 | -0,261714822 | 0,4488586 | 0,76624848 | AUP1_HUMAN |
| P18621 | -0,261274302 | 0,03138228 | 0,32121512 | RL17_HUMAN |
| P04632 | -0,259466178 | 0,19796891 | 0,58298692 | CPNS1_HUMAN |
| P62266 | -0,258769037 | 0,17901563 | 0,56097948 | RS23_HUMAN |
| Q96GC9 | -0,258161925 | 0,26329301 | 0,63824731 | VMP1_HUMAN |
| Q15056 | -0,257864268 | 0,17508422 | 0,55359362 | IF4H_HUMAN |
| Q8TD16 | -0,257669008 | 0,37553869 | 0,72949947 | BICD2_HUMAN |
| P26885 | -0,257465793 | 0,57024037 | 0,83904912 | FKBP2_HUMAN |
| Q8WUH6 | -0,257367625 | 0,56781998 | 0,83860775 | TM263_HUMAN |
| O15173 | -0,257176982 | 0,22264556 | 0,60546408 | PGRC2_HUMAN |
| Q53FA7 | -0,257014403 | 0,08166838 | 0,42518835 | QORX_HUMAN |
| O95292 | -0,256603736 | 0,32467369 | 0,69522089 | VAPB_HUMAN |
| O43290 | -0,256313405 | 0,09673612 | 0,44565474 | SNUT1_HUMAN |
| P51153 | -0,256290648 | 0,25097941 | 0,62782391 | RAB13_HUMAN |
| P36578 | -0,256121314 | 0,10985103 | 0,46569592 | RL4_HUMAN |
| P11216 | -0,255098584 | 0,04239914 | 0,34514615 | PYGB_HUMAN |
| Q9Y5P4 | -0,254681942 | 0,1677226 | 0,53904265 | C43BP_HUMAN |
| Q29RF7 | -0,253150591 | 0,06000644 | 0,38735155 | PDS5A_HUMAN |
| O00303 | -0,252683955 | 0,13828355 | 0,50533731 | EIF3F_HUMAN |
| Q9NZI8 | -0,251263714 | 0,04896322 | 0,36117836 | IF2B1_HUMAN |
| P06753 | -0,250061586 | 0,31300704 | 0,68018093 | TPM3_HUMAN |
| O00299 | -0,244506465 | 0,04001902 | 0,34486976 | CLIC1_HUMAN |
| P09496 | -0,244236223 | 0,20689574 | 0,59045894 | CLCA_HUMAN |
| Q5H9R7 | -0,243296996 | 0,25230117 | 0,6291425 | PP6R3_HUMAN |
| Q8NBF2 | -0,242925416 | 0,18449567 | 0,56902349 | NHLC2_HUMAN |
| P21266 | -0,241980117 | 0,13614934 | 0,5047871 | GSTM3_HUMAN |
| P55263 | -0,241527256 | 0,07844034 | 0,42518835 | ADK_HUMAN |
| Q99615 | -0,241453977 | 0,1457891 | 0,51236505 | DNJC7_HUMAN |
| O00159 | -0,241181409 | 0,04079139 | 0,34498692 | MYO1C_HUMAN |
| P61201 | -0,23828986 | 0,07628845 | 0,42042351 | CSN2_HUMAN |
| Q99986 | -0,237722195 | 0,33258266 | 0,6974601 | VRK1_HUMAN |
| Q9HCE1 | -0,237589008 | 0,16647922 | 0,53701003 | MOV10_HUMAN |
| Q6NUQ4 | -0,236822839 | 0,27153785 | 0,64508587 | TM214_HUMAN |
| P49773 | -0,235210761 | 0,30030139 | 0,66787317 | HINT1_HUMAN |
| O60504 | -0,235087711 | 0,34409486 | 0,70652307 | VINEX_HUMAN |
| Q9P000 | -0,235036036 | 0,52158452 | 0,81506274 | COMD9_HUMAN |
| P62913 | -0,233587962 | 0,07579936 | 0,4203846 | RL11_HUMAN |
| P30519 | -0,233110839 | 0,35307323 | 0,71280778 | HMOX2_HUMAN |
| P48637 | -0,231001273 | 0,14433697 | 0,51231408 | GSHB_HUMAN |
| Q9Y6A4 | -0,229102667 | 0,12995877 | 0,50212641 | CFA20_HUMAN |
| Q15758 | -0,228260201 | 0,17146027 | 0,54606368 | AAAT_HUMAN |
| Q6PKG0 | -0,228113889 | 0,17976886 | 0,56133865 | LARP1_HUMAN |
| P13473 | -0,227390062 | 0,57669302 | 0,83942825 | LAMP2_HUMAN |
| Q9Y4K1 | -0,22711344 | 0,0575513 | 0,37924458 | AIM1_HUMAN |
| Q8TDD1 | -0,226099995 | 0,44696258 | 0,76624848 | DDX54_HUMAN |
| Q5DJT8 | -0,226094733 | 0,24222309 | 0,62544131 | CT452_HUMAN |
| Q8WXX5 | -0,225872181 | 0,05697618 | 0,37924458 | DNJC9_HUMAN |
| P46821 | -0,225584411 | 0,0820119 | 0,42518835 | MAP1B_HUMAN |
| Q9Y5P6 | -0,224795519 | 0,04910218 | 0,36117836 | GMPPB_HUMAN |
| P57764 | -0,222622306 | 0,19310134 | 0,57952098 | GSDMD_HUMAN |
| Q96EK5 | -0,222189212 | 0,20103801 | 0,5839878 | KBP_HUMAN |
| O43776 | -0,221301055 | 0,11044378 | 0,46569592 | SYNC_HUMAN |
| Q8TDN6 | -0,220011621 | 0,61447672 | 0,85938749 | BRX1_HUMAN |
| Q9UHB6 | -0,219866105 | 0,0953231 | 0,44565474 | LIMA1_HUMAN |
| Q12788 | -0,219795093 | 0,06503793 | 0,40114205 | TBL3_HUMAN |
| Q96EY4 | -0,219338421 | 0,47137651 | 0,78275407 | TMA16_HUMAN |
| P61011 | -0,218659669 | 0,21802639 | 0,60171177 | SRP54_HUMAN |
| Q92905 | -0,218368737 | 0,08220437 | 0,42518835 | CSN5_HUMAN |
| P30622 | -0,21718976 | 0,07989245 | 0,42518835 | CLIP1_HUMAN |
| Q16563 | -0,216728693 | 0,24283277 | 0,62544131 | SYPL1_HUMAN |
| Q7RTV0 | -0,216022828 | 0,24153861 | 0,62544131 | PHF5A_HUMAN |
| P07900 | -0,215322428 | 0,05759858 | 0,37924458 | HS90A_HUMAN |
| O43837 | -0,215299219 | 0,67424632 | 0,88671542 | IDH3B_HUMAN |
| Q02543 | -0,214235322 | 0,22240221 | 0,60546408 | RL18A_HUMAN |
| Q96EL3 | -0,212927059 | 0,35489317 | 0,71280778 | RM53_HUMAN |
| Q9NY33 | -0,212392771 | 0,31037243 | 0,67644218 | DPP3_HUMAN |
| P49903 | -0,212331479 | 0,55969067 | 0,83455149 | SPS1_HUMAN |
| P11413 | -0,210629466 | 0,32597198 | 0,69560058 | G6PD_HUMAN |
| Q9NSE4 | -0,210506346 | 0,34734247 | 0,70838523 | SYIM_HUMAN |
| P42167 | -0,210224592 | 0,08668143 | 0,43536006 | LAP2B_HUMAN |
| P46734 | -0,209643312 | 0,40283995 | 0,74722994 | MP2K3_HUMAN |
| Q7Z6Z7 | -0,209399124 | 0,11628227 | 0,47430215 | HUWE1_HUMAN |
| Q16851 | -0,20888047 | 0,28693558 | 0,65938922 | UGPA_HUMAN |
| Q0JRZ9 | -0,207731112 | 0,25393318 | 0,63052901 | FCHO2_HUMAN |
| P63241 | -0,207201521 | 0,40239374 | 0,74722994 | IF5A1_HUMAN |
| Q96BY6 | -0,206109281 | 0,3604497 | 0,71439749 | DOC10_HUMAN |
| O75477 | -0,206085158 | 0,44370204 | 0,76624848 | ERLN1_HUMAN |
| Q5VZK9 | -0,204979782 | 0,12942956 | 0,50198119 | LR16A_HUMAN |
| Q9BXR0 | -0,204475229 | 0,44675426 | 0,76624848 | TGT_HUMAN |
| Q92615 | -0,204408068 | 0,37337751 | 0,72842986 | LAR4B_HUMAN |
| Q8NCW5 | -0,204091256 | 0,39038673 | 0,73612226 | NNRE_HUMAN |
| O00429 | -0,203768461 | 0,11553547 | 0,47430215 | DNM1L_HUMAN |
| Q86UE4 | -0,202873809 | 0,32180822 | 0,69245882 | LYRIC_HUMAN |
| P27824 | -0,202214034 | 0,11361983 | 0,4699851 | CALX_HUMAN |
| Q9BYD6 | -0,200727592 | 0,33278745 | 0,6974601 | RM01_HUMAN |
| P78417 | -0,200131648 | 0,18732331 | 0,57348817 | GSTO1_HUMAN |
| P07384 | -0,199378854 | 0,27783075 | 0,65036811 | CAN1_HUMAN |
| Q8N684 | -0,199332026 | 0,21956726 | 0,60302172 | CPSF7_HUMAN |
| P50416 | -0,198432188 | 0,07236788 | 0,41163083 | CPT1A_HUMAN |
| Q9HDC9 | -0,198092831 | 0,09041743 | 0,44314581 | APMAP_HUMAN |
| P62244 | -0,197410009 | 0,09265786 | 0,44565474 | RS15A_HUMAN |
| O00425 | -0,196959961 | 0,13722068 | 0,50533731 | IF2B3_HUMAN |
| O14639 | -0,195663955 | 0,34137159 | 0,70520711 | ABLM1_HUMAN |
| P62424 | -0,194558376 | 0,13639014 | 0,5047871 | RL7A_HUMAN |
| Q9NVA2 | -0,194557118 | 0,54494866 | 0,82030199 | SEP11_HUMAN |
| O43175 | -0,194532929 | 0,06713297 | 0,40142777 | SERA_HUMAN |
| Q15393 | -0,192877089 | 0,14198103 | 0,51043486 | SF3B3_HUMAN |
| Q9NW64 | -0,192544002 | 0,22967859 | 0,609012 | RBM22_HUMAN |
| Q9UJW0 | -0,192147283 | 0,28809994 | 0,66033859 | DCTN4_HUMAN |
| Q7Z739 | -0,191736391 | 0,17292983 | 0,54887616 | YTHD3_HUMAN |
| P47985 | -0,191719329 | 0,37457372 | 0,72842986 | UCRI_HUMAN |
| P31153 | -0,190561452 | 0,11185689 | 0,46625672 | METK2_HUMAN |
| Q16831 | -0,190238793 | 0,15642057 | 0,52149753 | UPP1_HUMAN |
| Q9UJU6 | -0,19006433 | 0,13515859 | 0,50376636 | DBNL_HUMAN |
| P04080 | -0,190055732 | 0,11192283 | 0,46625672 | CYTB_HUMAN |
| Q9Y3U8 | -0,189230532 | 0,29710734 | 0,66452252 | RL36_HUMAN |
| P62318 | -0,188074653 | 0,21634402 | 0,59897319 | SMD3_HUMAN |
| O00273 | -0,187515939 | 0,40281956 | 0,74722994 | DFFA_HUMAN |
| P31350 | -0,186324722 | 0,15465824 | 0,52086051 | RIR2_HUMAN |
| Q9UIG0 | -0,185415782 | 0,27201182 | 0,64533979 | BAZ1B_HUMAN |
| Q9UHD2 | -0,185256077 | 0,60711057 | 0,85905375 | TBK1_HUMAN |
| Q01105 | -0,184976876 | 0,34370727 | 0,70652307 | SET_HUMAN |
| Q16594 | -0,184576655 | 0,37993861 | 0,7339913 | TAF9_HUMAN |
| Q8TBC4 | -0,184569425 | 0,45724991 | 0,76974061 | UBA3_HUMAN |
| O00764 | -0,183182218 | 0,36860066 | 0,72397536 | PDXK_HUMAN |
| Q14847 | -0,18310814 | 0,1495859 | 0,51563141 | LASP1_HUMAN |
| P07858 | -0,18294092 | 0,27522548 | 0,65033117 | CATB_HUMAN |
| Q15067 | -0,181658746 | 0,49155988 | 0,79326333 | ACOX1_HUMAN |
| P62851 | -0,180995999 | 0,09243793 | 0,44565474 | RS25_HUMAN |
| Q12981 | -0,180144886 | 0,25099521 | 0,62782391 | SEC20_HUMAN |
| Q9Y3C6 | -0,179886058 | 0,50141431 | 0,79989688 | PPIL1_HUMAN |
| Q8N4X5 | -0,179600047 | 0,20940767 | 0,59281592 | AF1L2_HUMAN |
| P61088 | -0,179530547 | 0,15969486 | 0,52279992 | UBE2N_HUMAN |
| Q16513 | -0,179225746 | 0,25834704 | 0,63432137 | PKN2_HUMAN |
| P49588 | -0,177347311 | 0,12178397 | 0,48365997 | SYAC_HUMAN |
| Q6IBS0 | -0,177212491 | 0,52717384 | 0,81780144 | TWF2_HUMAN |
| P62487 | -0,177080261 | 0,23971019 | 0,62431188 | RPB7_HUMAN |
| P19525 | -0,176866835 | 0,29083433 | 0,66255294 | E2AK2_HUMAN |
| O00203 | -0,17632152 | 0,10604841 | 0,45694389 | AP3B1_HUMAN |
| Q92785 | -0,176176032 | 0,179685 | 0,56133865 | REQU_HUMAN |
| P02545 | -0,175899221 | 0,18686417 | 0,57348817 | LMNA_HUMAN |
| P60033 | -0,175737758 | 0,64045682 | 0,87364784 | CD81_HUMAN |
| P41227 | -0,175420442 | 0,33677242 | 0,70033965 | NAA10_HUMAN |
| P22570 | -0,174787561 | 0,47560175 | 0,7853205 | ADRO_HUMAN |
| Q15370 | -0,174397881 | 0,28309222 | 0,65569977 | ELOB_HUMAN |
| Q04323 | -0,173900234 | 0,69312478 | 0,89337104 | UBXN1_HUMAN |
| Q9UKM9 | -0,173617125 | 0,35640389 | 0,71280778 | RALY_HUMAN |
| P17480 | -0,173592288 | 0,24893361 | 0,62782391 | UBF1_HUMAN |
| P60174 | -0,173449625 | 0,43631928 | 0,76356053 | TPIS_HUMAN |
| Q9UN86 | -0,173439429 | 0,61679753 | 0,86022297 | G3BP2_HUMAN |
| Q13823 | -0,173366364 | 0,4336164 | 0,76229764 | NOG2_HUMAN |
| P60228 | -0,172539346 | 0,34653982 | 0,70756912 | EIF3E_HUMAN |
| Q7KZI7 | -0,172336009 | 0,25947355 | 0,63442907 | MARK2_HUMAN |
| Q00796 | -0,171660612 | 0,15892689 | 0,52279992 | DHSO_HUMAN |
| O15305 | -0,171047419 | 0,15707962 | 0,52149753 | PMM2_HUMAN |
| P62701 | -0,17104031 | 0,35076371 | 0,71123713 | RS4X_HUMAN |
| P49458 | -0,170790053 | 0,29687132 | 0,66452252 | SRP09_HUMAN |
| Q15029 | -0,170301329 | 0,16452275 | 0,53363652 | U5S1_HUMAN |
| O43264 | -0,169967105 | 0,15061767 | 0,51715988 | ZW10_HUMAN |
| P60981 | -0,169390214 | 0,12088997 | 0,48191514 | DEST_HUMAN |
| P30086 | -0,169278645 | 0,21012245 | 0,59314142 | PEBP1_HUMAN |
| P53999 | -0,168482992 | 0,29367002 | 0,66337984 | TCP4_HUMAN |
| Q07960 | -0,167547748 | 0,41021882 | 0,75403888 | RHG01_HUMAN |
| Q8TEX9 | -0,167380591 | 0,27124082 | 0,64508587 | IPO4_HUMAN |
| Q9BUQ8 | -0,167037027 | 0,16968277 | 0,54236783 | DDX23_HUMAN |
| Q9NZN4 | -0,166822115 | 0,30954936 | 0,67635742 | EHD2_HUMAN |
| P48047 | -0,165885843 | 0,30502377 | 0,67196965 | ATPO_HUMAN |
| Q13596 | -0,165763146 | 0,13143107 | 0,50303967 | SNX1_HUMAN |
| Q9NTX5 | -0,165177976 | 0,36634341 | 0,72120013 | ECHD1_HUMAN |
| Q13564 | -0,165040756 | 0,37915846 | 0,73328997 | ULA1_HUMAN |
| Q16531 | -0,164273222 | 0,1334914 | 0,50303967 | DDB1_HUMAN |
| Q9P2K5 | -0,16286495 | 0,20078504 | 0,5839878 | MYEF2_HUMAN |
| Q7L1Q6 | -0,1613151 | 0,65521472 | 0,8803959 | BZW1_HUMAN |
| O00116 | -0,161107418 | 0,42136659 | 0,75742583 | ADAS_HUMAN |
| P24666 | -0,159887636 | 0,3914925 | 0,73612226 | PPAC_HUMAN |
| P61758 | -0,158775367 | 0,24750766 | 0,62782391 | PFD3_HUMAN |
| P30046 | -0,158362749 | 0,34347457 | 0,70652307 | DOPD_HUMAN |
| Q92688 | -0,157771798 | 0,37714316 | 0,73138726 | AN32B_HUMAN |
| O14964 | -0,157645656 | 0,38541116 | 0,73612226 | HGS_HUMAN |
| P13639 | -0,157456659 | 0,14024271 | 0,50830271 | EF2_HUMAN |
| Q9H0B6 | -0,157266037 | 0,57580336 | 0,83942825 | KLC2_HUMAN |
| Q8WX93 | -0,157148637 | 0,3909743 | 0,73612226 | PALLD_HUMAN |
| Q15005 | -0,157123305 | 0,47225669 | 0,78275407 | SPCS2_HUMAN |
| Q93008 | -0,157001323 | 0,24349363 | 0,62544131 | USP9X_HUMAN |
| P53992 | -0,156234039 | 0,15363202 | 0,5201414 | SC24C_HUMAN |
| Q02978 | -0,156090209 | 0,38678376 | 0,73612226 | M2OM_HUMAN |
| P55327 | -0,155479969 | 0,28539124 | 0,65842232 | TPD52_HUMAN |
| P0C0S5 | -0,155450003 | 0,62468984 | 0,86070535 | H2AZ_HUMAN |
| P33316 | -0,154719175 | 0,21056911 | 0,59323798 | DUT_HUMAN |
| P62277 | -0,154517717 | 0,50071491 | 0,79989688 | RS13_HUMAN |
| P06730 | -0,154427962 | 0,16572009 | 0,5357054 | IF4E_HUMAN |
| P62249 | -0,154191014 | 0,23077871 | 0,60917264 | RS16_HUMAN |
| P16070 | -0,154099677 | 0,1743289 | 0,55219855 | CD44_HUMAN |
| Q16626 | -0,152937008 | 0,3745537 | 0,72842986 | MEA1_HUMAN |
| P18085 | -0,152346914 | 0,55794386 | 0,8333605 | ARF4_HUMAN |
| Q86X55 | -0,152190347 | 0,38317193 | 0,73612226 | CARM1_HUMAN |
| P62906 | -0,151614678 | 0,18106554 | 0,56438515 | RL10A_HUMAN |
| Q9BY77 | -0,151571891 | 0,33696358 | 0,70033965 | PDIP3_HUMAN |
| Q6P1N9 | -0,150219991 | 0,1441309 | 0,51231408 | TATD1_HUMAN |
| P05388 | -0,150158148 | 0,2355022 | 0,61792964 | RLA0_HUMAN |
| P00492 | -0,148874378 | 0,42103238 | 0,75742583 | HPRT_HUMAN |
| Q9NT62 | -0,148668628 | 0,36898858 | 0,72397536 | ATG3_HUMAN |
| Q96AE4 | -0,147360137 | 0,17808333 | 0,55905444 | FUBP1_HUMAN |
| Q86SF2 | -0,146770678 | 0,2695848 | 0,64270609 | GALT7_HUMAN |
| Q9Y6N5 | -0,146456687 | 0,20020466 | 0,5839878 | SQRD_HUMAN |
| P35250 | -0,146348127 | 0,19459435 | 0,5798252 | RFC2_HUMAN |
| P55786 | -0,146320142 | 0,24149116 | 0,62544131 | PSA_HUMAN |
| P60866 | -0,146137736 | 0,63387732 | 0,86936025 | RS20_HUMAN |
| P21281 | -0,145489542 | 0,47731822 | 0,78584605 | VATB2_HUMAN |
| O14561 | -0,145384046 | 0,58624213 | 0,8447653 | ACPM_HUMAN |
| Q16739 | -0,145023276 | 0,57672755 | 0,83942825 | CEGT_HUMAN |
| Q8WWY3 | -0,144783356 | 0,29451478 | 0,66379101 | PRP31_HUMAN |
| Q9UBT2 | -0,144779363 | 0,38789665 | 0,73612226 | SAE2_HUMAN |
| P22061 | -0,144332689 | 0,41720291 | 0,75690682 | PIMT_HUMAN |
| P30043 | -0,144124153 | 0,33286065 | 0,6974601 | BLVRB_HUMAN |
| P28838 | -0,143989979 | 0,2847625 | 0,65783506 | AMPL_HUMAN |
| P20290 | -0,143538566 | 0,21472845 | 0,59878389 | BTF3_HUMAN |
| Q9GZZ1 | -0,143092879 | 0,74068996 | 0,91126805 | NAA50_HUMAN |
| Q8WXI9 | -0,142445066 | 0,37003115 | 0,72464979 | P66B_HUMAN |
| O75832 | -0,142101288 | 0,66130596 | 0,8803959 | PSD10_HUMAN |
| P23396 | -0,141825454 | 0,18666702 | 0,57348817 | RS3_HUMAN |
| Q15459 | -0,141762768 | 0,23749282 | 0,62129818 | SF3A1_HUMAN |
| Q08380 | -0,141185172 | 0,43025304 | 0,76056043 | LG3BP_HUMAN |
| P26006 | -0,140107778 | 0,42582414 | 0,76056043 | ITA3_HUMAN |
| Q6P587 | -0,139991725 | 0,72665191 | 0,90535369 | FAHD1_HUMAN |
| Q96RS6 | -0,139671442 | 0,42767327 | 0,76056043 | NUDC1_HUMAN |
| P30153 | -0,139586533 | 0,25905537 | 0,63442907 | 2AAA_HUMAN |
| P51572 | -0,139178398 | 0,38829436 | 0,73612226 | BAP31_HUMAN |
| P09429 | -0,138965349 | 0,49693561 | 0,79781992 | HMGB1_HUMAN |
| Q15293 | -0,138791305 | 0,70851583 | 0,89868026 | RCN1_HUMAN |
| P62820 | -0,138501868 | 0,33194545 | 0,6974601 | RAB1A_HUMAN |
| Q8WW12 | -0,138375685 | 0,21093386 | 0,59329651 | PCNP_HUMAN |
| O43324 | -0,138114066 | 0,46730997 | 0,78006157 | MCA3_HUMAN |
| P42785 | -0,137203271 | 0,52938455 | 0,81780144 | PCP_HUMAN |
| Q13098 | -0,136907191 | 0,49590469 | 0,79762164 | CSN1_HUMAN |
| Q9UBQ0 | -0,135764865 | 0,35985975 | 0,71403323 | VPS29_HUMAN |
| Q9UNF1 | -0,135412445 | 0,38234821 | 0,73612226 | MAGD2_HUMAN |
| Q9NYF8 | -0,134059655 | 0,22324693 | 0,60546408 | BCLF1_HUMAN |
| Q92797 | -0,133014861 | 0,51118418 | 0,8088765 | SYMPK_HUMAN |
| Q8IWE2 | -0,13262863 | 0,4481484 | 0,76624848 | NXP20_HUMAN |
| Q14566 | -0,132519905 | 0,25772476 | 0,63432137 | MCM6_HUMAN |
| P12004 | -0,132339519 | 0,30410284 | 0,67162411 | PCNA_HUMAN |
| Q15020 | -0,13231107 | 0,44328717 | 0,76624848 | SART3_HUMAN |
| Q96KB5 | -0,132064557 | 0,2289329 | 0,60845586 | TOPK_HUMAN |
| P46108 | -0,130866425 | 0,33050143 | 0,6974601 | CRK_HUMAN |
| P61803 | -0,130731288 | 0,26605177 | 0,64158986 | DAD1_HUMAN |
| P00374 | -0,130589579 | 0,35909209 | 0,71403323 | DYR_HUMAN |
| Q7L2H7 | -0,129656694 | 0,76970474 | 0,92038128 | EIF3M_HUMAN |
| P46977 | -0,128169418 | 0,29361417 | 0,66337984 | STT3A_HUMAN |
| P29353 | -0,127879775 | 0,48297055 | 0,78982533 | SHC1_HUMAN |
| Q96FW1 | -0,126896304 | 0,36299981 | 0,71783314 | OTUB1_HUMAN |
| P54577 | -0,126394742 | 0,3239052 | 0,69442115 | SYYC_HUMAN |
| P62750 | -0,12632665 | 0,2532068 | 0,63049856 | RL23A_HUMAN |
| P04150 | -0,125735009 | 0,3314782 | 0,6974601 | GCR_HUMAN |
| Q14697 | -0,125388274 | 0,58459598 | 0,84446979 | GANAB_HUMAN |
| Q9NPQ8 | -0,125311879 | 0,56893354 | 0,83860775 | RIC8A_HUMAN |
| Q13464 | -0,123091179 | 0,4065171 | 0,74990249 | ROCK1_HUMAN |
| O75436 | -0,122525624 | 0,51326569 | 0,80901647 | VP26A_HUMAN |
| P09038 | -0,120099117 | 0,45493962 | 0,76969834 | FGF2_HUMAN |
| Q00325 | -0,120062663 | 0,28648618 | 0,65938922 | MPCP_HUMAN |
| P84098 | -0,119832849 | 0,5646195 | 0,83860775 | RL19_HUMAN |
| P10768 | -0,11955095 | 0,29149318 | 0,66255294 | ESTD_HUMAN |
| Q9H3S7 | -0,118334379 | 0,51267732 | 0,80901647 | PTN23_HUMAN |
| Q04760 | -0,118331434 | 0,58570402 | 0,84468225 | LGUL_HUMAN |
| P07910 | -0,117031265 | 0,35417898 | 0,71280778 | HNRPC_HUMAN |
| A5YKK6 | -0,116764131 | 0,30482272 | 0,67196965 | CNOT1_HUMAN |
| O75083 | -0,116672387 | 0,30398982 | 0,67162411 | WDR1_HUMAN |
| O75691 | -0,11638141 | 0,48774499 | 0,79007031 | UTP20_HUMAN |
| P08754 | -0,116224082 | 0,54453261 | 0,82030199 | GNAI3_HUMAN |
| P55084 | -0,116173762 | 0,30050493 | 0,66787317 | ECHB_HUMAN |
| Q13185 | -0,115405884 | 0,48615321 | 0,79007031 | CBX3_HUMAN |
| P52788 | -0,115096839 | 0,51116323 | 0,8088765 | SPSY_HUMAN |
| P00338 | -0,114915409 | 0,41342573 | 0,75543026 | LDHA_HUMAN |
| P35611 | -0,114388163 | 0,60506792 | 0,85852252 | ADDA_HUMAN |
| Q9Y6M1 | -0,11393827 | 0,40244487 | 0,74722994 | IF2B2_HUMAN |
| P11940 | -0,113266311 | 0,46300581 | 0,77583711 | PABP1_HUMAN |
| P39687 | -0,112717694 | 0,49184131 | 0,79326333 | AN32A_HUMAN |
| P53621 | -0,112465503 | 0,39054489 | 0,73612226 | COPA_HUMAN |
| Q5JSH3 | -0,112009399 | 0,41451194 | 0,75543026 | WDR44_HUMAN |
| P50570 | -0,111966519 | 0,37905393 | 0,73328997 | DYN2_HUMAN |
| Q16643 | -0,11111857 | 0,32977902 | 0,69717091 | DREB_HUMAN |
| P56182 | -0,110721243 | 0,56638609 | 0,83860775 | RRP1_HUMAN |
| P23921 | -0,110619087 | 0,27718038 | 0,65036811 | RIR1_HUMAN |
| Q9NZU5 | -0,110114679 | 0,41159505 | 0,75530699 | LMCD1_HUMAN |
| Q52LJ0 | -0,109621909 | 0,24336752 | 0,62544131 | FA98B_HUMAN |
| P05121 | -0,108678348 | 0,38079433 | 0,73483693 | PAI1_HUMAN |
| Q9UJS0 | -0,108346344 | 0,65812092 | 0,8803959 | CMC2_HUMAN |
| Q9Y3A5 | -0,108159846 | 0,38781098 | 0,73612226 | SBDS_HUMAN |
| P53602 | -0,10815167 | 0,69244444 | 0,89337104 | MVD1_HUMAN |
| Q04917 | -0,106776226 | 0,27503235 | 0,65033117 | 1433F_HUMAN |
| P04083 | -0,105913151 | 0,39267834 | 0,73674335 | ANXA1_HUMAN |
| P08473 | -0,104865229 | 0,44493245 | 0,76624848 | NEP_HUMAN |
| Q15365 | -0,104814669 | 0,39173869 | 0,73612226 | PCBP1_HUMAN |
| P18124 | -0,10479307 | 0,45050649 | 0,76712487 | RL7_HUMAN |
| P14866 | -0,103770672 | 0,31051697 | 0,67644218 | HNRPL_HUMAN |
| Q9UK76 | -0,103768794 | 0,5508878 | 0,82422192 | HN1_HUMAN |
| P62854 | -0,103238558 | 0,31382481 | 0,68111607 | RS26_HUMAN |
| Q9Y450 | -0,102460838 | 0,57485821 | 0,83942825 | HBS1L_HUMAN |
| P51452 | -0,101994327 | 0,35955558 | 0,71403323 | DUS3_HUMAN |
| O60493 | -0,101992676 | 0,68768426 | 0,89272795 | SNX3_HUMAN |
| P62910 | -0,100047717 | 0,40243043 | 0,74722994 | RL32_HUMAN |
| Q9BSC4 | -0,0999548 | 0,66306285 | 0,8803959 | NOL10_HUMAN |
| Q01813 | -0,099462468 | 0,47969963 | 0,78887928 | PFKAP_HUMAN |
| O43592 | -0,098992638 | 0,36145994 | 0,71559299 | XPOT_HUMAN |
| Q14166 | -0,098896254 | 0,59739016 | 0,85465044 | TTL12_HUMAN |
| P33121 | -0,098597067 | 0,64364846 | 0,87579618 | ACSL1_HUMAN |
| Q00341 | -0,098452924 | 0,3649874 | 0,71933616 | VIGLN_HUMAN |
| Q9HB07 | -0,096786571 | 0,44893967 | 0,76624848 | MYG1_HUMAN |
| Q3ZCQ8 | -0,096589586 | 0,71266507 | 0,90238683 | TIM50_HUMAN |
| O43847 | -0,096512057 | 0,34330985 | 0,70652307 | NRDC_HUMAN |
| Q9NSD9 | -0,096161403 | 0,32681479 | 0,69560058 | SYFB_HUMAN |
| P08581 | -0,096116483 | 0,57871845 | 0,83942825 | MET_HUMAN |
| P30405 | -0,095600224 | 0,53594684 | 0,81846545 | PPIF_HUMAN |
| P18859 | -0,095584662 | 0,45153587 | 0,76712487 | ATP5J_HUMAN |
| Q9UNH7 | -0,095283579 | 0,4599319 | 0,77226387 | SNX6_HUMAN |
| Q9NQC3 | -0,093350811 | 0,6620037 | 0,8803959 | RTN4_HUMAN |
| P62269 | -0,093316134 | 0,3584053 | 0,71403323 | RS18_HUMAN |
| O95347 | -0,093123697 | 0,64980131 | 0,87873132 | SMC2_HUMAN |
| Q9H6F5 | -0,09306485 | 0,610005 | 0,85905375 | CCD86_HUMAN |
| P07602 | -0,092697058 | 0,44808514 | 0,76624848 | SAP_HUMAN |
| Q9Y4P3 | -0,092603757 | 0,68890907 | 0,89272795 | TBL2_HUMAN |
| Q99536 | -0,092379806 | 0,37412035 | 0,72842986 | VAT1_HUMAN |
| Q9NTK5 | -0,092366701 | 0,57191973 | 0,83926118 | OLA1_HUMAN |
| Q15019 | -0,091950818 | 0,39192858 | 0,73612226 | SEPT2_HUMAN |
| E9PAV3 | -0,091636562 | 0,61643314 | 0,86022297 | NACAM_HUMAN |
| P09622 | -0,090942501 | 0,43671616 | 0,76356053 | DLDH_HUMAN |
| O95235 | -0,090559619 | 0,43046374 | 0,76056043 | KI20A_HUMAN |
| Q96K76 | -0,089910181 | 0,59844977 | 0,85465044 | UBP47_HUMAN |
| P17980 | -0,089808483 | 0,46768197 | 0,78006157 | PRS6A_HUMAN |
| O95400 | -0,089261154 | 0,42979258 | 0,76056043 | CD2B2_HUMAN |
| P43246 | -0,088806093 | 0,52042937 | 0,81492478 | MSH2_HUMAN |
| P27816 | -0,088207607 | 0,43089772 | 0,76056043 | MAP4_HUMAN |
| P38159 | -0,087582094 | 0,64959648 | 0,87873132 | RBMX_HUMAN |
| O43242 | -0,087268852 | 0,49661523 | 0,79781992 | PSMD3_HUMAN |
| Q9Y6C9 | -0,087119706 | 0,53138077 | 0,81846545 | MTCH2_HUMAN |
| P12236 | -0,086430914 | 0,72077374 | 0,90383607 | ADT3_HUMAN |
| P35658 | -0,086420687 | 0,66086456 | 0,8803959 | NU214_HUMAN |
| Q15942 | -0,086367695 | 0,57406249 | 0,83942825 | ZYX_HUMAN |
| P05198 | -0,085777073 | 0,43480836 | 0,76356053 | IF2A_HUMAN |
| P62888 | -0,084953177 | 0,77492164 | 0,92235087 | RL30_HUMAN |
| O00148 | -0,083233841 | 0,50508073 | 0,80210652 | DX39A_HUMAN |
| P12268 | -0,082872677 | 0,41595932 | 0,75543026 | IMDH2_HUMAN |
| Q9Y5Y2 | -0,082818923 | 0,54059305 | 0,81949948 | NUBP2_HUMAN |
| Q8N163 | -0,082110453 | 0,48060195 | 0,78888724 | CCAR2_HUMAN |
| Q96B49 | -0,081562994 | 0,68260234 | 0,89272795 | TOM6_HUMAN |
| Q9Y3B8 | -0,081333951 | 0,48832268 | 0,79007031 | ORN_HUMAN |
| Q9NR28 | -0,081013546 | 0,86170878 | 0,95324529 | DBLOH_HUMAN |
| Q9Y6B6 | -0,080646115 | 0,63397033 | 0,86936025 | SAR1B_HUMAN |
| Q13435 | -0,080490976 | 0,47619548 | 0,7853205 | SF3B2_HUMAN |
| P15311 | -0,080472111 | 0,44091273 | 0,76593339 | EZRI_HUMAN |
| P60953 | -0,080267795 | 0,68232353 | 0,89272795 | CDC42_HUMAN |
| P09661 | -0,0801783 | 0,70328612 | 0,8952766 | RU2A_HUMAN |
| Q9BTV4 | -0,079872475 | 0,6098286 | 0,85905375 | TMM43_HUMAN |
| P30566 | -0,07973627 | 0,57135457 | 0,83926118 | PUR8_HUMAN |
| P08107 | -0,079279498 | 0,52056913 | 0,81492478 | HSP71_HUMAN |
| P60891 | -0,078702311 | 0,5421376 | 0,81949948 | PRPS1_HUMAN |
| P22102 | -0,078452623 | 0,57612467 | 0,83942825 | PUR2_HUMAN |
| O76021 | -0,07809996 | 0,5925166 | 0,85101648 | RL1D1_HUMAN |
| Q9UQE7 | -0,077525024 | 0,51502499 | 0,81057649 | SMC3_HUMAN |
| P63279 | -0,077395835 | 0,52912069 | 0,81780144 | UBC9_HUMAN |
| P42566 | -0,077390075 | 0,75460307 | 0,91407243 | EPS15_HUMAN |
| O14980 | -0,077220879 | 0,41502883 | 0,75543026 | XPO1_HUMAN |
| Q96AY3 | -0,07572059 | 0,47591074 | 0,7853205 | FKB10_HUMAN |
| Q86TB9 | -0,075595209 | 0,83134263 | 0,94487473 | PATL1_HUMAN |
| P52701 | -0,075579372 | 0,52264953 | 0,81600166 | MSH6_HUMAN |
| P55884 | -0,075383839 | 0,47740818 | 0,78584605 | EIF3B_HUMAN |
| P68104 | -0,075301265 | 0,48710896 | 0,79007031 | EF1A1_HUMAN |
| Q06203 | -0,074177246 | 0,70823183 | 0,89868026 | PUR1_HUMAN |
| Q12996 | -0,073569959 | 0,72431697 | 0,90444117 | CSTF3_HUMAN |
| P29218 | -0,073246976 | 0,58944804 | 0,84799481 | IMPA1_HUMAN |
| P09936 | -0,073243914 | 0,57299493 | 0,83942825 | UCHL1_HUMAN |
| Q9H0H5 | -0,072742541 | 0,7281077 | 0,90588346 | RGAP1_HUMAN |
| P45974 | -0,071906797 | 0,60910101 | 0,85905375 | UBP5_HUMAN |
| Q09028 | -0,071489113 | 0,61155678 | 0,85926222 | RBBP4_HUMAN |
| P08238 | -0,071291809 | 0,44346484 | 0,76624848 | HS90B_HUMAN |
| O15031 | -0,07125272 | 0,84398794 | 0,94867699 | PLXB2_HUMAN |
| Q9BXP5 | -0,071234342 | 0,74333429 | 0,91241584 | SRRT_HUMAN |
| Q96HE7 | -0,071220407 | 0,70155644 | 0,8952766 | ERO1A_HUMAN |
| Q71RC2 | -0,070676101 | 0,73617612 | 0,90884664 | LARP4_HUMAN |
| Q05397 | -0,069858128 | 0,48141185 | 0,7894795 | FAK1_HUMAN |
| P09104 | -0,069312598 | 0,59788896 | 0,85465044 | ENOG_HUMAN |
| Q7Z434 | -0,068946212 | 0,61257607 | 0,85938749 | MAVS_HUMAN |
| O15143 | -0,067785002 | 0,64170406 | 0,87450833 | ARC1B_HUMAN |
| P15531 | -0,067244782 | 0,57988608 | 0,84042847 | NDKA_HUMAN |
| O14618 | -0,066605851 | 0,62310769 | 0,86070535 | CCS_HUMAN |
| Q93052 | -0,066269281 | 0,65272354 | 0,8803959 | LPP_HUMAN |
| P12081 | -0,066136865 | 0,61159311 | 0,85926222 | SYHC_HUMAN |
| P45880 | -0,066098103 | 0,57846066 | 0,83942825 | VDAC2_HUMAN |
| P34897 | -0,065600622 | 0,6549323 | 0,8803959 | GLYM_HUMAN |
| P17655 | -0,064080046 | 0,62491476 | 0,86070535 | CAN2_HUMAN |
| O43809 | -0,063752296 | 0,68966145 | 0,89272795 | CPSF5_HUMAN |
| Q8NBS9 | -0,063705337 | 0,52828472 | 0,81780144 | TXND5_HUMAN |
| Q15050 | -0,062786569 | 0,72761756 | 0,90588346 | RRS1_HUMAN |
| P35237 | -0,062600443 | 0,50401879 | 0,80186881 | SPB6_HUMAN |
| O75874 | -0,062237413 | 0,64660982 | 0,87757839 | IDHC_HUMAN |
| Q9H307 | -0,062155718 | 0,62412567 | 0,86070535 | PININ_HUMAN |
| P62316 | -0,062056061 | 0,62399439 | 0,86070535 | SMD2_HUMAN |
| Q05519 | -0,061681678 | 0,89045198 | 0,96235081 | SRS11_HUMAN |
| P26639 | -0,06161179 | 0,5258904 | 0,81780144 | SYTC_HUMAN |
| P31948 | -0,061424213 | 0,53336224 | 0,81846545 | STIP1_HUMAN |
| Q63HN8 | -0,061097988 | 0,85373103 | 0,95100032 | RN213_HUMAN |
| Q04637 | -0,060963818 | 0,5266195 | 0,81780144 | IF4G1_HUMAN |
| P61247 | -0,060932511 | 0,53633231 | 0,81846545 | RS3A_HUMAN |
| P62899 | -0,06061072 | 0,68446268 | 0,89272795 | RL31_HUMAN |
| P28482 | -0,060435319 | 0,7061047 | 0,89756476 | MK01_HUMAN |
| Q15149 | -0,060206995 | 0,5837457 | 0,84446979 | PLEC_HUMAN |
| P46776 | -0,060170111 | 0,54564322 | 0,82030199 | RL27A_HUMAN |
| P46778 | -0,059272013 | 0,79059657 | 0,92925413 | RL21_HUMAN |
| O15400 | -0,058934127 | 0,69050006 | 0,89272795 | STX7_HUMAN |
| Q92890 | -0,058803282 | 0,56070541 | 0,83535602 | UFD1_HUMAN |
| Q99873 | -0,058760776 | 0,56396567 | 0,83860775 | ANM1_HUMAN |
| P22234 | -0,058737465 | 0,60155058 | 0,85629629 | PUR6_HUMAN |
| P48723 | -0,058716642 | 0,80307027 | 0,93249507 | HSP13_HUMAN |
| Q9BVG4 | -0,058453448 | 0,66217107 | 0,8803959 | PBDC1_HUMAN |
| O43854 | -0,057942254 | 0,74385205 | 0,91241584 | EDIL3_HUMAN |
| P40763 | -0,057607373 | 0,64042833 | 0,87364784 | STAT3_HUMAN |
| P26641 | -0,056764551 | 0,64414361 | 0,87579618 | EF1G_HUMAN |
| Q9Y4Y9 | -0,056579753 | 0,82426406 | 0,94278218 | LSM5_HUMAN |
| O96005 | -0,056194079 | 0,76847218 | 0,92038128 | CLPT1_HUMAN |
| P55265 | -0,055508553 | 0,71415445 | 0,90238683 | DSRAD_HUMAN |
| Q14258 | -0,055431743 | 0,83385294 | 0,945092 | TRI25_HUMAN |
| Q6P2Q9 | -0,054708706 | 0,61596721 | 0,86022297 | PRP8_HUMAN |
| Q96B97 | -0,054217856 | 0,66583617 | 0,8803959 | SH3K1_HUMAN |
| P05387 | -0,053702867 | 0,75119488 | 0,91269533 | RLA2_HUMAN |
| P16152 | -0,053492755 | 0,73367844 | 0,90736714 | CBR1_HUMAN |
| Q8TF05 | -0,053347023 | 0,76208765 | 0,91826599 | PP4R1_HUMAN |
| P53618 | -0,052483748 | 0,69315023 | 0,89337104 | COPB_HUMAN |
| Q13630 | -0,052354811 | 0,79845852 | 0,93220096 | FCL_HUMAN |
| Q92917 | -0,052288895 | 0,68675913 | 0,89272795 | GPKOW_HUMAN |
| Q99426 | -0,051838673 | 0,60480632 | 0,85852252 | TBCB_HUMAN |
| Q9BWD1 | -0,05160228 | 0,79818554 | 0,93220096 | THIC_HUMAN |
| O43390 | -0,051570617 | 0,61306036 | 0,85938749 | HNRPR_HUMAN |
| P06756 | -0,051332029 | 0,80721985 | 0,93299967 | ITAV_HUMAN |
| Q9Y3A3 | -0,049627396 | 0,8133393 | 0,93760688 | PHOCN_HUMAN |
| Q8TCS8 | -0,048970201 | 0,7502246 | 0,91269533 | PNPT1_HUMAN |
| Q8IY81 | -0,048371882 | 0,69062003 | 0,89272795 | SPB1_HUMAN |
| Q16740 | -0,048123721 | 0,88174866 | 0,95893944 | CLPP_HUMAN |
| Q9UNM6 | -0,047924694 | 0,67244567 | 0,88551272 | PSD13_HUMAN |
| P40429 | -0,047791574 | 0,91866985 | 0,96826459 | RL13A_HUMAN |
| Q13442 | -0,046743965 | 0,6847099 | 0,89272795 | HAP28_HUMAN |
| P55010 | -0,046508299 | 0,81719301 | 0,9389708 | IF5_HUMAN |
| Q9UBE0 | -0,046076029 | 0,6817156 | 0,89272795 | SAE1_HUMAN |
| Q9UJ70 | -0,045966388 | 0,74026649 | 0,91126805 | NAGK_HUMAN |
| P40123 | -0,045679931 | 0,70786403 | 0,89868026 | CAP2_HUMAN |
| Q06210 | -0,045494883 | 0,81987985 | 0,94082818 | GFPT1_HUMAN |
| Q9UKF6 | -0,045429918 | 0,9025495 | 0,96279248 | CPSF3_HUMAN |
| O94979 | -0,045355208 | 0,72886124 | 0,90617967 | SC31A_HUMAN |
| Q9BRJ6 | -0,045262299 | 0,87277198 | 0,95575575 | CG050_HUMAN |
| Q9Y5X1 | -0,045261557 | 0,70283889 | 0,8952766 | SNX9_HUMAN |
| P63173 | -0,045015189 | 0,81622244 | 0,93846897 | RL38_HUMAN |
| Q9UMS0 | -0,044994341 | 0,70082447 | 0,8952766 | NFU1_HUMAN |
| Q9P2R7 | -0,044728702 | 0,74781267 | 0,91241584 | SUCB1_HUMAN |
| Q15075 | -0,044684686 | 0,68457487 | 0,89272795 | EEA1_HUMAN |
| Q92616 | -0,044445342 | 0,79712224 | 0,93220096 | GCN1L_HUMAN |
| Q7L014 | -0,044030624 | 0,70055204 | 0,8952766 | DDX46_HUMAN |
| P26373 | -0,043941362 | 0,69941958 | 0,8952766 | RL13_HUMAN |
| Q02818 | -0,043811911 | 0,77004177 | 0,92038128 | NUCB1_HUMAN |
| P48059 | -0,043506276 | 0,79030628 | 0,92925413 | LIMS1_HUMAN |
| O95816 | -0,042932221 | 0,78282808 | 0,92425236 | BAG2_HUMAN |
| Q7Z2X7 | -0,042535262 | 0,68801869 | 0,89272795 | PAGE2_HUMAN |
| P49736 | -0,042388726 | 0,64695199 | 0,87757839 | MCM2_HUMAN |
| Q13085 | -0,042326213 | 0,72083181 | 0,90383607 | ACACA_HUMAN |
| P63104 | -0,042296486 | 0,66669528 | 0,8803959 | 1433Z_HUMAN |
| Q8TCT9 | -0,042130598 | 0,8027898 | 0,93249507 | HM13_HUMAN |
| O95861 | -0,040415858 | 0,83804032 | 0,94627891 | BPNT1_HUMAN |
| O15355 | -0,039988056 | 0,77215817 | 0,92038128 | PPM1G_HUMAN |
| Q9Y266 | -0,039633315 | 0,77221979 | 0,92038128 | NUDC_HUMAN |
| Q9Y3F4 | -0,039432303 | 0,65919932 | 0,8803959 | STRAP_HUMAN |
| O75367 | -0,038627634 | 0,75231138 | 0,91274218 | H2AY_HUMAN |
| Q9Y2J8 | -0,037824479 | 0,85416923 | 0,95100032 | PADI2_HUMAN |
| Q16555 | -0,037796513 | 0,78171198 | 0,92417597 | DPYL2_HUMAN |
| Q9BWM7 | -0,0377563 | 0,79973966 | 0,93220096 | SFXN3_HUMAN |
| Q15582 | -0,037303442 | 0,72576855 | 0,90489441 | BGH3_HUMAN |
| Q96S52 | -0,036943711 | 0,92632126 | 0,96875239 | PIGS_HUMAN |
| P08559 | -0,036379315 | 0,84227932 | 0,94796866 | ODPA_HUMAN |
| P61421 | -0,03598994 | 0,80683045 | 0,93299967 | VA0D1_HUMAN |
| P05556 | -0,035912111 | 0,7243867 | 0,90444117 | ITB1_HUMAN |
| O75531 | -0,035698819 | 0,83970142 | 0,94627891 | BAF_HUMAN |
| O95456 | -0,035576988 | 0,87262869 | 0,95575575 | PSMG1_HUMAN |
| P49756 | -0,035367092 | 0,9022811 | 0,96279248 | RBM25_HUMAN |
| P40939 | -0,035317497 | 0,7546636 | 0,91407243 | ECHA_HUMAN |
| Q96FZ7 | -0,034678885 | 0,73068876 | 0,90652847 | CHMP6_HUMAN |
| P46777 | -0,034576233 | 0,76623046 | 0,92038128 | RL5_HUMAN |
| P62333 | -0,034146114 | 0,85104476 | 0,95038042 | PRS10_HUMAN |
| Q9H3K6 | -0,033795075 | 0,80263102 | 0,93249507 | BOLA2_HUMAN |
| Q14696 | -0,033723765 | 0,73446157 | 0,90736714 | MESD_HUMAN |
| Q9Y371 | -0,033310585 | 0,87198881 | 0,95575575 | SHLB1_HUMAN |
| O94776 | -0,033160274 | 0,77956861 | 0,92406404 | MTA2_HUMAN |
| O94874 | -0,032540213 | 0,81593876 | 0,93846897 | UFL1_HUMAN |
| P62826 | -0,032535947 | 0,72489056 | 0,90444117 | RAN_HUMAN |
| Q15642 | -0,03195741 | 0,88567183 | 0,96079992 | CIP4_HUMAN |
| P49591 | -0,030980589 | 0,87338894 | 0,95575575 | SYSC_HUMAN |
| O75157 | -0,030557565 | 0,88134048 | 0,95893944 | T22D2_HUMAN |
| P62081 | -0,029817222 | 0,82818392 | 0,94406079 | RS7_HUMAN |
| P62829 | -0,02963309 | 0,83921429 | 0,94627891 | RL23_HUMAN |
| Q86W42 | -0,029349846 | 0,8239477 | 0,94278218 | THOC6_HUMAN |
| P09960 | -0,02934146 | 0,79155221 | 0,92925413 | LKHA4_HUMAN |
| O95782 | -0,029299367 | 0,87025074 | 0,95575575 | AP2A1_HUMAN |
| P61158 | -0,029047529 | 0,74789034 | 0,91241584 | ARP3_HUMAN |
| Q8NHH9 | -0,028972186 | 0,83802766 | 0,94627891 | ATLA2_HUMAN |
| P51970 | -0,02855623 | 0,90566523 | 0,96390938 | NDUA8_HUMAN |
| Q9NZZ3 | -0,027852316 | 0,87017675 | 0,95575575 | CHMP5_HUMAN |
| Q01780 | -0,02774135 | 0,83239619 | 0,94488249 | EXOSX_HUMAN |
| Q9BPX3 | -0,027327137 | 0,80603828 | 0,93286063 | CND3_HUMAN |
| P17987 | -0,027173753 | 0,77337796 | 0,92113717 | TCPA_HUMAN |
| Q9NY61 | -0,02716965 | 0,83542262 | 0,945092 | AATF_HUMAN |
| P30048 | -0,0268753 | 0,93411534 | 0,97126851 | PRDX3_HUMAN |
| P33993 | -0,026750024 | 0,85260767 | 0,95046562 | MCM7_HUMAN |
| Q14980 | -0,026562024 | 0,81298662 | 0,93760688 | NUMA1_HUMAN |
| P49411 | -0,025710538 | 0,80593009 | 0,93286063 | EFTU_HUMAN |
| Q14651 | -0,025055949 | 0,88647183 | 0,96079992 | PLSI_HUMAN |
| Q9UHD8 | -0,024923113 | 0,82807851 | 0,94406079 | SEPT9_HUMAN |
| P40121 | -0,024842195 | 0,86159722 | 0,95324529 | CAPG_HUMAN |
| P30050 | -0,024757418 | 0,82275138 | 0,94278218 | RL12_HUMAN |
| Q99714 | -0,024748017 | 0,86100584 | 0,95324529 | HCD2_HUMAN |
| Q8TAT6 | -0,024518342 | 0,79182178 | 0,92925413 | NPL4_HUMAN |
| P20340 | -0,02447509 | 0,85145004 | 0,95038042 | RAB6A_HUMAN |
| Q99471 | -0,024417252 | 0,82294157 | 0,94278218 | PFD5_HUMAN |
| P11142 | -0,024362845 | 0,79624269 | 0,93220096 | HSP7C_HUMAN |
| Q15366 | -0,02332144 | 0,89281318 | 0,96279248 | PCBP2_HUMAN |
| Q08257 | -0,023227941 | 0,83351455 | 0,945092 | QOR_HUMAN |
| P46779 | -0,022987692 | 0,8758376 | 0,95575575 | RL28_HUMAN |
| O15372 | -0,022610001 | 0,86374789 | 0,95441156 | EIF3H_HUMAN |
| P22314 | -0,022049266 | 0,80838033 | 0,93372708 | UBA1_HUMAN |
| Q8TC07 | -0,021758104 | 0,84976485 | 0,95036262 | TBC15_HUMAN |
| Q7L5N1 | -0,021610933 | 0,91619313 | 0,96826459 | CSN6_HUMAN |
| Q9UGV2 | -0,021481598 | 0,88613493 | 0,96079992 | NDRG3_HUMAN |
| Q96C19 | -0,021293614 | 0,86763829 | 0,95575575 | EFHD2_HUMAN |
| O95573 | -0,021099862 | 0,88173865 | 0,95893944 | ACSL3_HUMAN |
| P33176 | -0,020925967 | 0,85225056 | 0,95046562 | KINH_HUMAN |
| Q86U42 | -0,020754045 | 0,9675368 | 0,98595293 | PABP2_HUMAN |
| Q15287 | -0,020571324 | 0,87535627 | 0,95575575 | RNPS1_HUMAN |
| O00541 | -0,020417961 | 0,94440262 | 0,97604927 | PESC_HUMAN |
| Q9H2G2 | -0,02018612 | 0,88202792 | 0,95893944 | SLK_HUMAN |
| Q9HB71 | -0,019888766 | 0,83537661 | 0,945092 | CYBP_HUMAN |
| P05026 | -0,019745459 | 0,92568981 | 0,96866826 | AT1B1_HUMAN |
| Q08211 | -0,019200145 | 0,87457788 | 0,95575575 | DHX9_HUMAN |
| O15145 | -0,018518266 | 0,95157608 | 0,98026996 | ARPC3_HUMAN |
| Q9Y265 | -0,018285387 | 0,86168702 | 0,95324529 | RUVB1_HUMAN |
| P07195 | -0,0180791 | 0,87129949 | 0,95575575 | LDHB_HUMAN |
| O60664 | -0,017766815 | 0,89119154 | 0,96235548 | PLIN3_HUMAN |
| Q9Y3B7 | -0,017586652 | 0,91829756 | 0,96826459 | RM11_HUMAN |
| P80303 | -0,01743434 | 0,89837825 | 0,96279248 | NUCB2_HUMAN |
| P62879 | -0,017041503 | 0,9561591 | 0,98275391 | GBB2_HUMAN |
| O00217 | -0,016243518 | 0,93572888 | 0,97129664 | NDUS8_HUMAN |
| P07942 | -0,01619175 | 0,87232169 | 0,95575575 | LAMB1_HUMAN |
| P06576 | -0,016027532 | 0,86026679 | 0,95324529 | ATPB_HUMAN |
| O60701 | -0,015956691 | 0,87542083 | 0,95575575 | UGDH_HUMAN |
| Q6P2E9 | -0,015570642 | 0,91900675 | 0,96826459 | EDC4_HUMAN |
| P55735 | -0,015560686 | 0,95434069 | 0,9817033 | SEC13_HUMAN |
| Q92734 | -0,015509389 | 0,91233656 | 0,96719857 | TFG_HUMAN |
| O75131 | -0,015288542 | 0,96671906 | 0,98578429 | CPNE3_HUMAN |
| P21333 | -0,014716664 | 0,89942215 | 0,96279248 | FLNA_HUMAN |
| P56192 | -0,014662997 | 0,90137254 | 0,96279248 | SYMC_HUMAN |
| Q8N766 | -0,014558285 | 0,8898506 | 0,96235081 | EMC1_HUMAN |
| P61313 | -0,014519359 | 0,93969976 | 0,97426899 | RL15_HUMAN |
| O14828 | -0,013881035 | 0,94196997 | 0,97468111 | SCAM3_HUMAN |
| P55209 | -0,012968697 | 0,91304261 | 0,96719857 | NP1L1_HUMAN |
| Q9Y2R0 | -0,01263931 | 0,92971189 | 0,96941489 | COA3_HUMAN |
| Q01082 | -0,012570793 | 0,89905843 | 0,96279248 | SPTB2_HUMAN |
| Q13045 | -0,011984004 | 0,9126877 | 0,96719857 | FLII_HUMAN |
| Q9Y305 | -0,011934538 | 0,92112812 | 0,96826459 | ACOT9_HUMAN |
| Q14764 | -0,011797685 | 0,92121551 | 0,96826459 | MVP_HUMAN |
| Q99614 | -0,011558071 | 0,95916692 | 0,98275391 | TTC1_HUMAN |
| Q7L576 | -0,011541069 | 0,9199169 | 0,96826459 | CYFP1_HUMAN |
| Q6YN16 | -0,011484011 | 0,93425202 | 0,97126851 | HSDL2_HUMAN |
| Q92598 | -0,01109086 | 0,92393751 | 0,96828504 | HS105_HUMAN |
| O60610 | -0,011013062 | 0,92789421 | 0,96924422 | DIAP1_HUMAN |
| P07954 | -0,01085161 | 0,96882269 | 0,98595293 | FUMH_HUMAN |
| O94826 | -0,01079462 | 0,96127625 | 0,9836249 | TOM70_HUMAN |
| Q96EY1 | -0,010602476 | 0,96392174 | 0,98407341 | DNJA3_HUMAN |
| O95793 | -0,010216574 | 0,92523687 | 0,96866826 | STAU1_HUMAN |
| Q12931 | -0,009817611 | 0,94138116 | 0,97464551 | TRAP1_HUMAN |
| Q15003 | -0,009743322 | 0,96448804 | 0,98408007 | CND2_HUMAN |
| P38117 | -0,009496345 | 0,93290324 | 0,97126851 | ETFB_HUMAN |
| Q07955 | -0,008345163 | 0,9404633 | 0,97426899 | SRSF1_HUMAN |
| Q9UBS4 | -0,008288429 | 0,96909502 | 0,98595293 | DJB11_HUMAN |
| Q9UPN3 | -0,007775293 | 0,94345406 | 0,97564249 | MACF1_HUMAN |
| P63220 | -0,007322118 | 0,96180387 | 0,9836249 | RS21_HUMAN |
| P61978 | -0,006681683 | 0,95792158 | 0,98275391 | HNRPK_HUMAN |
| P23458 | -0,006459234 | 0,96925404 | 0,98595293 | JAK1_HUMAN |
| P60842 | -0,006190126 | 0,95983417 | 0,98275391 | IF4A1_HUMAN |
| P52732 | -0,005705341 | 0,97138204 | 0,98595293 | KIF11_HUMAN |
| Q9UHD9 | -0,005517498 | 0,97588864 | 0,98906689 | UBQL2_HUMAN |
| P09543 | -0,005323976 | 0,98348148 | 0,99193747 | CN37_HUMAN |
| P52209 | -0,005207236 | 0,95964873 | 0,98275391 | 6PGD_HUMAN |
| O43615 | -0,005086351 | 0,98423259 | 0,99193747 | TIM44_HUMAN |
| P53396 | -0,004183848 | 0,96307256 | 0,98407341 | ACLY_HUMAN |
| O00567 | -0,00404139 | 0,9825342 | 0,99193747 | NOP56_HUMAN |
| Q96N66 | -0,003431952 | 0,98597576 | 0,99193747 | MBOA7_HUMAN |
| Q13287 | -0,003313411 | 0,97975222 | 0,99045681 | NMI_HUMAN |
| O75489 | -0,003260391 | 0,9923155 | 0,99571384 | NDUS3_HUMAN |
| P02794 | -0,002719278 | 0,98742354 | 0,99193747 | FRIH_HUMAN |
| P43686 | -0,002704675 | 0,98517057 | 0,99193747 | PRS6B_HUMAN |
| Q6PK04 | -0,002409167 | 0,98499451 | 0,99193747 | CC137_HUMAN |
| Q9Y2W1 | -0,000727966 | 0,99699527 | 0,99869954 | TR150_HUMAN |
| Q06323 | -0,000131001 | 0,99887048 | 0,99943899 | PSME1_HUMAN |
| P61163 | -5,68E-05 | 0,99982943 | 0,99982943 | ACTZ_HUMAN |
| P50991 | 0,000232498 | 0,99802248 | 0,99915918 | TCPD_HUMAN |
| Q96R06 | 0,000777673 | 0,99584534 | 0,99868574 | SPAG5_HUMAN |
| P06493 | 0,000792308 | 0,99661039 | 0,99869954 | CDK1_HUMAN |
| Q14152 | 0,001662601 | 0,98704358 | 0,99193747 | EIF3A_HUMAN |
| P60660 | 0,002272991 | 0,9863769 | 0,99193747 | MYL6_HUMAN |
| P36871 | 0,002415525 | 0,98986085 | 0,99381803 | PGM1_HUMAN |
| Q99733 | 0,002989386 | 0,97871543 | 0,98997797 | NP1L4_HUMAN |
| P46926 | 0,003069301 | 0,98573034 | 0,99193747 | GNPI1_HUMAN |
| P04406 | 0,003127251 | 0,97163587 | 0,98595293 | G3P_HUMAN |
| P35579 | 0,003503159 | 0,97042541 | 0,98595293 | MYH9_HUMAN |
| P49915 | 0,003583607 | 0,9785474 | 0,98997797 | GUAA_HUMAN |
| P35251 | 0,003889482 | 0,97612688 | 0,98906689 | RFC1_HUMAN |
| Q13867 | 0,003955753 | 0,98557409 | 0,99193747 | BLMH_HUMAN |
| Q13895 | 0,003976731 | 0,97193198 | 0,98595293 | BYST_HUMAN |
| P49368 | 0,004218634 | 0,97049931 | 0,98595293 | TCPG_HUMAN |
| P55060 | 0,005236485 | 0,95879837 | 0,98275391 | XPO2_HUMAN |
| P00352 | 0,005433013 | 0,95085199 | 0,98026996 | AL1A1_HUMAN |
| P48643 | 0,005985152 | 0,95918979 | 0,98275391 | TCPE_HUMAN |
| O75533 | 0,006437684 | 0,9518321 | 0,98026996 | SF3B1_HUMAN |
| Q00610 | 0,006915284 | 0,94027238 | 0,97426899 | CLH1_HUMAN |
| P50402 | 0,007101833 | 0,97693971 | 0,98932028 | EMD_HUMAN |
| Q96AG4 | 0,007201067 | 0,94855596 | 0,97861583 | LRC59_HUMAN |
| P06733 | 0,007407652 | 0,93321361 | 0,97126851 | ENOA_HUMAN |
| P11766 | 0,009250468 | 0,92422201 | 0,96828504 | ADHX_HUMAN |
| Q15691 | 0,009770608 | 0,935836 | 0,97129664 | MARE1_HUMAN |
| P54920 | 0,00977683 | 0,92785055 | 0,96924422 | SNAA_HUMAN |
| O75886 | 0,010173368 | 0,92943699 | 0,96941489 | STAM2_HUMAN |
| P08670 | 0,010368826 | 0,90715874 | 0,96478226 | VIME_HUMAN |
| Q00839 | 0,010580132 | 0,91328192 | 0,96719857 | HNRPU_HUMAN |
| O75643 | 0,010724654 | 0,94625367 | 0,97681383 | U520_HUMAN |
| P11586 | 0,010838162 | 0,92144862 | 0,96826459 | C1TC_HUMAN |
| Q9C0B1 | 0,011024897 | 0,91974164 | 0,96826459 | FTO_HUMAN |
| Q7Z4H8 | 0,011266419 | 0,92869436 | 0,96941489 | KDEL2_HUMAN |
| Q9NP79 | 0,011327123 | 0,91399763 | 0,96737377 | VTA1_HUMAN |
| P49419 | 0,0116736 | 0,92351364 | 0,96828504 | AL7A1_HUMAN |
| Q9Y262 | 0,012278015 | 0,8958687 | 0,96279248 | EIF3L_HUMAN |
| Q99460 | 0,012883249 | 0,89546928 | 0,96279248 | PSMD1_HUMAN |
| P62805 | 0,013099573 | 0,89046332 | 0,96235081 | H4_HUMAN |
| Q92979 | 0,013792932 | 0,90116165 | 0,96279248 | NEP1_HUMAN |
| P61160 | 0,013827661 | 0,9528786 | 0,98077317 | ARP2_HUMAN |
| O14579 | 0,013972577 | 0,96356427 | 0,98407341 | COPE_HUMAN |
| O00139 | 0,014229216 | 0,94594097 | 0,97681383 | KIF2A_HUMAN |
| P61604 | 0,014338229 | 0,87229208 | 0,95575575 | CH10_HUMAN |
| Q9UBB4 | 0,014433455 | 0,8989178 | 0,96279248 | ATX10_HUMAN |
| P23528 | 0,014438247 | 0,91202335 | 0,96719857 | COF1_HUMAN |
| Q16881 | 0,015324597 | 0,89385045 | 0,96279248 | TRXR1_HUMAN |
| P00558 | 0,015552411 | 0,89803586 | 0,96279248 | PGK1_HUMAN |
| P43307 | 0,015626561 | 0,9057897 | 0,96390938 | SSRA_HUMAN |
| Q9Y6I3 | 0,015896637 | 0,93593658 | 0,97129664 | EPN1_HUMAN |
| O00151 | 0,0162401 | 0,88640759 | 0,96079992 | PDLI1_HUMAN |
| Q15369 | 0,0165922 | 0,92310744 | 0,96828504 | ELOC_HUMAN |
| O00154 | 0,017194356 | 0,89889569 | 0,96279248 | BACH_HUMAN |
| P83731 | 0,018393219 | 0,87755871 | 0,95703983 | RL24_HUMAN |
| Q9NYU2 | 0,018397931 | 0,93216143 | 0,97126851 | UGGG1_HUMAN |
| P51149 | 0,018883093 | 0,89366503 | 0,96279248 | RAB7A_HUMAN |
| P41091 | 0,019055633 | 0,86215018 | 0,95324529 | IF2G_HUMAN |
| P56537 | 0,019869235 | 0,90400314 | 0,96375836 | IF6_HUMAN |
| P06748 | 0,019949356 | 0,84981231 | 0,95036262 | NPM_HUMAN |
| Q99829 | 0,020115107 | 0,92237422 | 0,96828504 | CPNE1_HUMAN |
| P08865 | 0,020137305 | 0,84907055 | 0,95036262 | RSSA_HUMAN |
| P60983 | 0,020759269 | 0,95783828 | 0,98275391 | GMFB_HUMAN |
| Q02878 | 0,020989305 | 0,86198666 | 0,95324529 | RL6_HUMAN |
| Q13740 | 0,021219375 | 0,90560303 | 0,96390938 | CD166_HUMAN |
| P39060 | 0,021670662 | 0,88708918 | 0,96087664 | COIA1_HUMAN |
| P42765 | 0,021987362 | 0,84339205 | 0,9486137 | THIM_HUMAN |
| P40227 | 0,022343134 | 0,88361902 | 0,96007555 | TCPZ_HUMAN |
| Q86VP6 | 0,022418661 | 0,82543564 | 0,9430535 | CAND1_HUMAN |
| O95817 | 0,023424253 | 0,83071819 | 0,94487473 | BAG3_HUMAN |
| O60749 | 0,023718917 | 0,85788323 | 0,95324529 | SNX2_HUMAN |
| O60841 | 0,024087436 | 0,84636895 | 0,95013832 | IF2P_HUMAN |
| P09211 | 0,024207176 | 0,81817667 | 0,93948699 | GSTP1_HUMAN |
| Q9Y490 | 0,024387358 | 0,80568511 | 0,93286063 | TLN1_HUMAN |
| P10155 | 0,024914847 | 0,89773342 | 0,96279248 | RO60_HUMAN |
| O00505 | 0,025623973 | 0,86798502 | 0,95575575 | IMA4_HUMAN |
| Q969T9 | 0,025644637 | 0,85114133 | 0,95038042 | WBP2_HUMAN |
| Q9UBC2 | 0,025726575 | 0,82557414 | 0,9430535 | EP15R_HUMAN |
| O95757 | 0,025839315 | 0,91498762 | 0,96783889 | HS74L_HUMAN |
| Q08752 | 0,025928954 | 0,8415443 | 0,94774817 | PPID_HUMAN |
| Q15424 | 0,026099852 | 0,89775002 | 0,96279248 | SAFB1_HUMAN |
| Q15021 | 0,026241155 | 0,83813849 | 0,94627891 | CND1_HUMAN |
| P55039 | 0,026241367 | 0,8481356 | 0,95036262 | DRG2_HUMAN |
| Q13263 | 0,026316325 | 0,80399467 | 0,93286063 | TIF1B_HUMAN |
| Q06830 | 0,026450297 | 0,78031496 | 0,92406404 | PRDX1_HUMAN |
| Q86X76 | 0,026513602 | 0,91294321 | 0,96719857 | NIT1_HUMAN |
| P22059 | 0,027685901 | 0,90214681 | 0,96279248 | OSBP1_HUMAN |
| O95336 | 0,027876216 | 0,84576638 | 0,95006855 | 6PGL_HUMAN |
| P13798 | 0,02788487 | 0,86575224 | 0,95542526 | ACPH_HUMAN |
| Q16181 | 0,027924977 | 0,83255004 | 0,94488249 | SEPT7_HUMAN |
| Q99832 | 0,028197087 | 0,77856902 | 0,92406404 | TCPH_HUMAN |
| P42704 | 0,028411607 | 0,78270105 | 0,92425236 | LPPRC_HUMAN |
| O60832 | 0,028508731 | 0,8906398 | 0,96235081 | DKC1_HUMAN |
| P31939 | 0,028735166 | 0,79960255 | 0,93220096 | PUR9_HUMAN |
| P20810 | 0,029329308 | 0,77823361 | 0,92406404 | ICAL_HUMAN |
| Q09161 | 0,029469015 | 0,83927778 | 0,94627891 | NCBP1_HUMAN |
| Q9Y314 | 0,029982235 | 0,84861422 | 0,95036262 | NOSIP_HUMAN |
| Q96KP4 | 0,030102675 | 0,75719951 | 0,91407243 | CNDP2_HUMAN |
| P05141 | 0,03043077 | 0,89991483 | 0,96279248 | ADT2_HUMAN |
| P27797 | 0,030645519 | 0,82914099 | 0,94406079 | CALR_HUMAN |
| P50990 | 0,03074976 | 0,75546526 | 0,91407243 | TCPQ_HUMAN |
| P31040 | 0,03113924 | 0,88057215 | 0,95893944 | SDHA_HUMAN |
| P62258 | 0,031454823 | 0,75688826 | 0,91407243 | 1433E_HUMAN |
| P30041 | 0,031546038 | 0,7842396 | 0,92522577 | PRDX6_HUMAN |
| Q15398 | 0,032078016 | 0,86467866 | 0,95483988 | DLGP5_HUMAN |
| P09874 | 0,032205163 | 0,85471745 | 0,95100841 | PARP1_HUMAN |
| P50579 | 0,032224816 | 0,79950433 | 0,93220096 | MAP2_HUMAN |
| P07437 | 0,032500141 | 0,73412223 | 0,90736714 | TBB5_HUMAN |
| P06744 | 0,033177761 | 0,76676892 | 0,92038128 | G6PI_HUMAN |
| P14314 | 0,03330463 | 0,79390661 | 0,93045855 | GLU2B_HUMAN |
| Q15233 | 0,033474055 | 0,78693768 | 0,92723622 | NONO_HUMAN |
| Q9BQE5 | 0,033551174 | 0,80069593 | 0,93220096 | APOL2_HUMAN |
| P27708 | 0,034055213 | 0,76837466 | 0,92038128 | PYR1_HUMAN |
| Q14676 | 0,03444825 | 0,81207479 | 0,93737852 | MDC1_HUMAN |
| P35659 | 0,034459005 | 0,79271603 | 0,92968297 | DEK_HUMAN |
| O43583 | 0,034823676 | 0,91072664 | 0,96719857 | DENR_HUMAN |
| Q99623 | 0,035202616 | 0,76809542 | 0,92038128 | PHB2_HUMAN |
| Q96P70 | 0,035401894 | 0,77179178 | 0,92038128 | IPO9_HUMAN |
| O43396 | 0,035505646 | 0,81418005 | 0,93796102 | TXNL1_HUMAN |
| P51659 | 0,035613165 | 0,71613702 | 0,90238683 | DHB4_HUMAN |
| Q9NR45 | 0,035846406 | 0,75756743 | 0,91407243 | SIAS_HUMAN |
| Q9Y2S0 | 0,035879258 | 0,81607369 | 0,93846897 | RPAC2_HUMAN |
| P14618 | 0,036084242 | 0,72132082 | 0,90383607 | KPYM_HUMAN |
| P39748 | 0,036199196 | 0,86893178 | 0,95575575 | FEN1_HUMAN |
| Q04446 | 0,037083214 | 0,78093741 | 0,92406404 | GLGB_HUMAN |
| O43491 | 0,037751248 | 0,71823024 | 0,90244115 | E41L2_HUMAN |
| Q9P0I2 | 0,03808584 | 0,87569998 | 0,95575575 | EMC3_HUMAN |
| Q96A49 | 0,038295315 | 0,87493477 | 0,95575575 | SYAP1_HUMAN |
| P47897 | 0,038402956 | 0,73030218 | 0,90652847 | SYQ_HUMAN |
| Q7KZF4 | 0,038659351 | 0,73790149 | 0,91033742 | SND1_HUMAN |
| Q14790 | 0,038871003 | 0,8920695 | 0,96271221 | CASP8_HUMAN |
| O95372 | 0,039185973 | 0,91935157 | 0,96826459 | LYPA2_HUMAN |
| Q14978 | 0,03925709 | 0,86150064 | 0,95324529 | NOLC1_HUMAN |
| O00487 | 0,039354056 | 0,82425834 | 0,94278218 | PSDE_HUMAN |
| P24821 | 0,039614409 | 0,7498586 | 0,91269533 | TENA_HUMAN |
| P43243 | 0,040023937 | 0,72471606 | 0,90444117 | MATR3_HUMAN |
| Q6P1J9 | 0,04035154 | 0,78109167 | 0,92406404 | CDC73_HUMAN |
| Q7Z5R6 | 0,040448776 | 0,6966371 | 0,8952766 | AB1IP_HUMAN |
| P62140 | 0,040595228 | 0,75645074 | 0,91407243 | PP1B_HUMAN |
| P52272 | 0,041426609 | 0,68861897 | 0,89272795 | HNRPM_HUMAN |
| P07814 | 0,041893623 | 0,66575539 | 0,8803959 | SYEP_HUMAN |
| Q8N183 | 0,042064638 | 0,72319568 | 0,90444117 | MIMIT_HUMAN |
| Q9NVN8 | 0,042119552 | 0,76417021 | 0,91951488 | GNL3L_HUMAN |
| Q9NZD2 | 0,042350563 | 0,7592669 | 0,91549466 | GLTP_HUMAN |
| Q8WWM7 | 0,042838943 | 0,77194372 | 0,92038128 | ATX2L_HUMAN |
| Q4G0F5 | 0,043049686 | 0,83146827 | 0,94487473 | VP26B_HUMAN |
| O75348 | 0,043242998 | 0,85687497 | 0,95280594 | VATG1_HUMAN |
| Q8TAQ2 | 0,0441462 | 0,70507619 | 0,89690589 | SMRC2_HUMAN |
| O75369 | 0,044161462 | 0,68488118 | 0,89272795 | FLNB_HUMAN |
| P24752 | 0,044390272 | 0,74124762 | 0,91126805 | THIL_HUMAN |
| P31150 | 0,045070203 | 0,66277419 | 0,8803959 | GDIA_HUMAN |
| P62979 | 0,045173768 | 0,71633512 | 0,90238683 | RS27A_HUMAN |
| Q9Y512 | 0,045350354 | 0,74714425 | 0,91241584 | SAM50_HUMAN |
| O60264 | 0,045864495 | 0,61751081 | 0,86022297 | SMCA5_HUMAN |
| P39019 | 0,045983466 | 0,78470514 | 0,92522577 | RS19_HUMAN |
| Q13601 | 0,045999657 | 0,76887545 | 0,92038128 | KRR1_HUMAN |
| Q13148 | 0,046313107 | 0,73161179 | 0,90703351 | TADBP_HUMAN |
| Q9Y6H1 | 0,046325286 | 0,79104997 | 0,92925413 | CHCH2_HUMAN |
| P12955 | 0,046390085 | 0,77546425 | 0,92237222 | PEPD_HUMAN |
| Q9Y696 | 0,046756319 | 0,78010124 | 0,92406404 | CLIC4_HUMAN |
| P10599 | 0,046786504 | 0,6282741 | 0,8642456 | THIO_HUMAN |
| Q53HL2 | 0,048449576 | 0,80040171 | 0,93220096 | BOREA_HUMAN |
| P48556 | 0,048638422 | 0,63799695 | 0,87351919 | PSMD8_HUMAN |
| P78527 | 0,048663932 | 0,62038194 | 0,86070535 | PRKDC_HUMAN |
| P10606 | 0,048850039 | 0,82770493 | 0,94406079 | COX5B_HUMAN |
| O60256 | 0,048922534 | 0,75110765 | 0,91269533 | KPRB_HUMAN |
| O00571 | 0,049244834 | 0,70106484 | 0,8952766 | DDX3X_HUMAN |
| Q07065 | 0,04927137 | 0,7464721 | 0,91241584 | CKAP4_HUMAN |
| Q13200 | 0,049657911 | 0,6204895 | 0,86070535 | PSMD2_HUMAN |
| Q9H910 | 0,049919577 | 0,61194329 | 0,85926222 | HN1L_HUMAN |
| Q96I99 | 0,04996031 | 0,74700587 | 0,91241584 | SUCB2_HUMAN |
| P52888 | 0,050134985 | 0,61926748 | 0,86061046 | THOP1_HUMAN |
| Q9UMX5 | 0,050434534 | 0,7169721 | 0,90238683 | NENF_HUMAN |
| P13010 | 0,050460126 | 0,62273857 | 0,86070535 | XRCC5_HUMAN |
| Q13155 | 0,05046283 | 0,74068239 | 0,91126805 | AIMP2_HUMAN |
| P47756 | 0,05075644 | 0,71743111 | 0,90238683 | CAPZB_HUMAN |
| Q9Y237 | 0,051437073 | 0,69827377 | 0,8952766 | PIN4_HUMAN |
| Q5JWF2 | 0,051645782 | 0,7018542 | 0,8952766 | GNAS1_HUMAN |
| P09012 | 0,052158287 | 0,8351687 | 0,945092 | SNRPA_HUMAN |
| P27635 | 0,052294537 | 0,79725023 | 0,93220096 | RL10_HUMAN |
| P78371 | 0,052349091 | 0,57816692 | 0,83942825 | TCPB_HUMAN |
| Q9BY43 | 0,05241792 | 0,84907785 | 0,95036262 | CHM4A_HUMAN |
| P30044 | 0,0527848 | 0,68281279 | 0,89272795 | PRDX5_HUMAN |
| O75347 | 0,054188126 | 0,64270236 | 0,87519035 | TBCA_HUMAN |
| Q9H4A4 | 0,054243579 | 0,65502722 | 0,8803959 | AMPB_HUMAN |
| P33991 | 0,054244104 | 0,70039702 | 0,8952766 | MCM4_HUMAN |
| Q9BZE1 | 0,054668458 | 0,74087022 | 0,91126805 | RM37_HUMAN |
| Q8IX12 | 0,054721887 | 0,73406496 | 0,90736714 | CCAR1_HUMAN |
| Q14315 | 0,055441799 | 0,60721614 | 0,85905375 | FLNC_HUMAN |
| O43707 | 0,055903835 | 0,54693655 | 0,82040482 | ACTN4_HUMAN |
| Q9UHX1 | 0,056023239 | 0,5737376 | 0,83942825 | PUF60_HUMAN |
| P11047 | 0,056062362 | 0,66451923 | 0,8803959 | LAMC1_HUMAN |
| P13995 | 0,056121556 | 0,80596432 | 0,93286063 | MTDC_HUMAN |
| P26358 | 0,05641482 | 0,61423465 | 0,85938749 | DNMT1_HUMAN |
| P05783 | 0,056730413 | 0,56583331 | 0,83860775 | K1C18_HUMAN |
| Q07020 | 0,056961515 | 0,75069031 | 0,91269533 | RL18_HUMAN |
| P35270 | 0,05713781 | 0,70274209 | 0,8952766 | SPRE_HUMAN |
| O43143 | 0,057688723 | 0,61032886 | 0,85905375 | DHX15_HUMAN |
| Q13177 | 0,059131018 | 0,70304792 | 0,8952766 | PAK2_HUMAN |
| Q9Y4L1 | 0,059215947 | 0,54593477 | 0,82030199 | HYOU1_HUMAN |
| P07686 | 0,059448126 | 0,68590935 | 0,89272795 | HEXB_HUMAN |
| Q12907 | 0,059693014 | 0,76518043 | 0,92010068 | LMAN2_HUMAN |
| Q13561 | 0,05975961 | 0,63993103 | 0,87364784 | DCTN2_HUMAN |
| Q12906 | 0,059971055 | 0,61372124 | 0,85938749 | ILF3_HUMAN |
| O43399 | 0,060183924 | 0,59812579 | 0,85465044 | TPD54_HUMAN |
| Q15645 | 0,06028273 | 0,66675855 | 0,8803959 | PCH2_HUMAN |
| P46782 | 0,060295165 | 0,55146489 | 0,82438373 | RS5_HUMAN |
| O75475 | 0,060698428 | 0,77068403 | 0,92038128 | PSIP1_HUMAN |
| P15880 | 0,060810103 | 0,61011612 | 0,85905375 | RS2_HUMAN |
| Q9UBQ5 | 0,061143297 | 0,69008341 | 0,89272795 | EIF3K_HUMAN |
| Q9P0L0 | 0,061983893 | 0,59973112 | 0,85509109 | VAPA_HUMAN |
| P07741 | 0,062040983 | 0,71434788 | 0,90238683 | APT_HUMAN |
| P55145 | 0,062076198 | 0,60945957 | 0,85905375 | MANF_HUMAN |
| Q07866 | 0,062423286 | 0,62895181 | 0,86450139 | KLC1_HUMAN |
| Q15102 | 0,062526683 | 0,71604593 | 0,90238683 | PA1B3_HUMAN |
| Q9Y4W2 | 0,062906059 | 0,74425313 | 0,91241584 | LAS1L_HUMAN |
| P05023 | 0,063253229 | 0,65715871 | 0,8803959 | AT1A1_HUMAN |
| P62195 | 0,063970464 | 0,53551528 | 0,81846545 | PRS8_HUMAN |
| Q9ULC4 | 0,064031911 | 0,66993774 | 0,8835338 | MCTS1_HUMAN |
| P27695 | 0,064203264 | 0,51668778 | 0,81246612 | APEX1_HUMAN |
| Q96EA4 | 0,064218137 | 0,59779281 | 0,85465044 | SPDLY_HUMAN |
| P37802 | 0,064386407 | 0,53598032 | 0,81846545 | TAGL2_HUMAN |
| O43660 | 0,064417116 | 0,91670854 | 0,96826459 | PLRG1_HUMAN |
| Q96GQ7 | 0,064532971 | 0,54142576 | 0,81949948 | DDX27_HUMAN |
| Q14683 | 0,064645836 | 0,69587581 | 0,8952766 | SMC1A_HUMAN |
| Q9Y285 | 0,065064497 | 0,48782617 | 0,79007031 | SYFA_HUMAN |
| P35998 | 0,065739007 | 0,60065922 | 0,85572034 | PRS7_HUMAN |
| Q9Y263 | 0,06582684 | 0,73007315 | 0,90652847 | PLAP_HUMAN |
| O43747 | 0,066978176 | 0,60226178 | 0,85661505 | AP1G1_HUMAN |
| P68036 | 0,067427192 | 0,68558227 | 0,89272795 | UB2L3_HUMAN |
| Q15147 | 0,067460623 | 0,53380267 | 0,81846545 | PLCB4_HUMAN |
| P14625 | 0,06750306 | 0,5777279 | 0,83942825 | ENPL_HUMAN |
| Q8IY67 | 0,068418072 | 0,74605919 | 0,91241584 | RAVR1_HUMAN |
| O14737 | 0,069153677 | 0,75138089 | 0,91269533 | PDCD5_HUMAN |
| Q93050 | 0,06933576 | 0,69766689 | 0,8952766 | VPP1_HUMAN |
| Q8IYB3 | 0,069388948 | 0,4975637 | 0,79809944 | SRRM1_HUMAN |
| Q9BYD2 | 0,069848336 | 0,74759254 | 0,91241584 | RM09_HUMAN |
| O75312 | 0,070524126 | 0,52933194 | 0,81780144 | ZPR1_HUMAN |
| Q99575 | 0,070684554 | 0,80196831 | 0,93249507 | POP1_HUMAN |
| P46940 | 0,070699575 | 0,5011092 | 0,79989688 | IQGA1_HUMAN |
| Q5BJH7 | 0,070832597 | 0,7155863 | 0,90238683 | YIF1B_HUMAN |
| P22626 | 0,070862775 | 0,62499522 | 0,86070535 | ROA2_HUMAN |
| Q9P2J5 | 0,070963652 | 0,43924642 | 0,76379689 | SYLC_HUMAN |
| Q9H9B4 | 0,071027799 | 0,51047934 | 0,8088765 | SFXN1_HUMAN |
| P25685 | 0,071631088 | 0,67436776 | 0,88671542 | DNJB1_HUMAN |
| P11021 | 0,071667238 | 0,4388577 | 0,76379689 | GRP78_HUMAN |
| Q8IZL8 | 0,071794601 | 0,65638849 | 0,8803959 | PELP1_HUMAN |
| Q13443 | 0,071950443 | 0,65080853 | 0,87874148 | ADAM9_HUMAN |
| P49321 | 0,072309506 | 0,5361358 | 0,81846545 | NASP_HUMAN |
| P49790 | 0,072970518 | 0,66254118 | 0,8803959 | NU153_HUMAN |
| Q12792 | 0,073595559 | 0,51849713 | 0,81403613 | TWF1_HUMAN |
| P82979 | 0,073763213 | 0,5189411 | 0,81403613 | SARNP_HUMAN |
| Q96CW1 | 0,073869206 | 0,58846898 | 0,84727967 | AP2M1_HUMAN |
| Q9P289 | 0,07429062 | 0,50086044 | 0,79989688 | STK26_HUMAN |
| P06737 | 0,074560405 | 0,53198643 | 0,81846545 | PYGL_HUMAN |
| Q2M2I8 | 0,07490398 | 0,64002359 | 0,87364784 | AAK1_HUMAN |
| P13667 | 0,075454845 | 0,44745884 | 0,76624848 | PDIA4_HUMAN |
| Q9H444 | 0,075804932 | 0,50464095 | 0,80210652 | CHM4B_HUMAN |
| Q09666 | 0,076290896 | 0,4828446 | 0,78982533 | AHNK_HUMAN |
| P30520 | 0,076354331 | 0,44413527 | 0,76624848 | PURA2_HUMAN |
| P04843 | 0,076356806 | 0,54489389 | 0,82030199 | RPN1_HUMAN |
| P15586 | 0,076955123 | 0,60662909 | 0,85905375 | GNS_HUMAN |
| P12956 | 0,077018617 | 0,59555637 | 0,85465044 | XRCC6_HUMAN |
| P31689 | 0,077158477 | 0,53878652 | 0,81947087 | DNJA1_HUMAN |
| P49959 | 0,077545824 | 0,53581735 | 0,81846545 | MRE11_HUMAN |
| O60869 | 0,07756489 | 0,66682552 | 0,8803959 | EDF1_HUMAN |
| Q96DV4 | 0,077609608 | 0,60904252 | 0,85905375 | RM38_HUMAN |
| Q9ULT8 | 0,077713765 | 0,71026813 | 0,90025333 | HECD1_HUMAN |
| Q86V48 | 0,078319749 | 0,66676981 | 0,8803959 | LUZP1_HUMAN |
| Q13813 | 0,078387664 | 0,41468476 | 0,75543026 | SPTN1_HUMAN |
| Q5VYK3 | 0,078708048 | 0,71866758 | 0,90244115 | ECM29_HUMAN |
| P84095 | 0,078883126 | 0,60341075 | 0,85755546 | RHOG_HUMAN |
| P30876 | 0,079673049 | 0,52669785 | 0,81780144 | RPB2_HUMAN |
| P11177 | 0,080156063 | 0,46901661 | 0,78076071 | ODPB_HUMAN |
| Q99613 | 0,080199317 | 0,42038407 | 0,75742583 | EIF3C_HUMAN |
| Q8IZP0 | 0,080311859 | 0,67495592 | 0,88682549 | ABI1_HUMAN |
| P27348 | 0,080363464 | 0,52125109 | 0,81506274 | 1433T_HUMAN |
| Q9BY32 | 0,080377517 | 0,54687369 | 0,82040482 | ITPA_HUMAN |
| O00625 | 0,080614087 | 0,54903608 | 0,82215113 | PIR_HUMAN |
| Q14195 | 0,080628075 | 0,54213745 | 0,81949948 | DPYL3_HUMAN |
| P29401 | 0,080738266 | 0,46943349 | 0,78076071 | TKT_HUMAN |
| Q9UIA9 | 0,081043251 | 0,48872965 | 0,79007031 | XPO7_HUMAN |
| Q9BWF3 | 0,081159771 | 0,5133343 | 0,80901647 | RBM4_HUMAN |
| P52815 | 0,081314008 | 0,69168032 | 0,89337104 | RM12_HUMAN |
| P04062 | 0,081922089 | 0,63263427 | 0,86888363 | GLCM_HUMAN |
| Q14192 | 0,082276003 | 0,55948192 | 0,83455149 | FHL2_HUMAN |
| Q13126 | 0,082502052 | 0,6175207 | 0,86022297 | MTAP_HUMAN |
| Q9BQ61 | 0,083007459 | 0,58526588 | 0,84468225 | CS043_HUMAN |
| Q9Y5K5 | 0,083055277 | 0,45517272 | 0,76969834 | UCHL5_HUMAN |
| Q9Y5A9 | 0,083196289 | 0,6355775 | 0,87088484 | YTHD2_HUMAN |
| O75400 | 0,083809141 | 0,42675796 | 0,76056043 | PR40A_HUMAN |
| Q01844 | 0,084052057 | 0,45755344 | 0,76974061 | EWS_HUMAN |
| P78318 | 0,084407796 | 0,54457841 | 0,82030199 | IGBP1_HUMAN |
| Q15434 | 0,084589517 | 0,48702795 | 0,79007031 | RBMS2_HUMAN |
| P61923 | 0,084819286 | 0,41765677 | 0,75694908 | COPZ1_HUMAN |
| P16435 | 0,085087301 | 0,7636472 | 0,91951488 | NCPR_HUMAN |
| P49023 | 0,085438757 | 0,65963335 | 0,8803959 | PAXI_HUMAN |
| O75844 | 0,085494404 | 0,66705765 | 0,8803959 | FACE1_HUMAN |
| Q15738 | 0,085575097 | 0,82900558 | 0,94406079 | NSDHL_HUMAN |
| Q10567 | 0,085877435 | 0,50332458 | 0,80148969 | AP1B1_HUMAN |
| P63244 | 0,085943419 | 0,5840567 | 0,84446979 | GBLP_HUMAN |
| O60220 | 0,086091762 | 0,48049398 | 0,78888724 | TIM8A_HUMAN |
| P49753 | 0,086775571 | 0,52550697 | 0,81780144 | ACOT2_HUMAN |
| Q15008 | 0,087358907 | 0,42220717 | 0,7581616 | PSMD6_HUMAN |
| Q14677 | 0,087444422 | 0,53697954 | 0,81874244 | EPN4_HUMAN |
| Q9NR50 | 0,087451258 | 0,53925058 | 0,81947087 | EI2BG_HUMAN |
| Q16891 | 0,087599952 | 0,5024376 | 0,80080263 | MIC60_HUMAN |
| Q9UG63 | 0,087610953 | 0,49409118 | 0,79616158 | ABCF2_HUMAN |
| Q96FV9 | 0,088274109 | 0,5703434 | 0,83904912 | THOC1_HUMAN |
| P00387 | 0,088348705 | 0,44439051 | 0,76624848 | NB5R3_HUMAN |
| P25398 | 0,088468128 | 0,48695484 | 0,79007031 | RS12_HUMAN |
| Q9BYT8 | 0,088518144 | 0,42592714 | 0,76056043 | NEUL_HUMAN |
| Q9Y2B0 | 0,088546686 | 0,5103203 | 0,8088765 | CNPY2_HUMAN |
| P37837 | 0,088997891 | 0,40924582 | 0,75335513 | TALDO_HUMAN |
| Q86UP2 | 0,089077074 | 0,53354143 | 0,81846545 | KTN1_HUMAN |
| P62241 | 0,08917371 | 0,38663614 | 0,73612226 | RS8_HUMAN |
| P48634 | 0,089404215 | 0,5677578 | 0,83860775 | PRC2A_HUMAN |
| O60828 | 0,09015595 | 0,51221211 | 0,80901647 | PQBP1_HUMAN |
| Q96CS3 | 0,09042263 | 0,45337713 | 0,76934073 | FAF2_HUMAN |
| Q7Z2W4 | 0,090589838 | 0,45163495 | 0,76712487 | ZCCHV_HUMAN |
| O15144 | 0,09176094 | 0,49495852 | 0,79682882 | ARPC2_HUMAN |
| Q92499 | 0,092051648 | 0,35609547 | 0,71280778 | DDX1_HUMAN |
| O76003 | 0,092270994 | 0,33488044 | 0,69903473 | GLRX3_HUMAN |
| Q6UXN9 | 0,092452648 | 0,53932184 | 0,81947087 | WDR82_HUMAN |
| P54578 | 0,092606439 | 0,40336391 | 0,74722994 | UBP14_HUMAN |
| P31930 | 0,092609834 | 0,52799938 | 0,81780144 | QCR1_HUMAN |
| P23381 | 0,092851461 | 0,64942121 | 0,87873132 | SYWC_HUMAN |
| Q9Y5X3 | 0,09287416 | 0,59076605 | 0,84919601 | SNX5_HUMAN |
| Q9NP72 | 0,092952425 | 0,4465047 | 0,76624848 | RAB18_HUMAN |
| P55769 | 0,093034948 | 0,47208073 | 0,78275407 | NH2L1_HUMAN |
| O95433 | 0,093156643 | 0,62136075 | 0,86070535 | AHSA1_HUMAN |
| P61081 | 0,093435342 | 0,54112993 | 0,81949948 | UBC12_HUMAN |
| O00186 | 0,093621234 | 0,53887773 | 0,81947087 | STXB3_HUMAN |
| P26038 | 0,09368991 | 0,39416965 | 0,73875293 | MOES_HUMAN |
| P04844 | 0,093925716 | 0,35872741 | 0,71403323 | RPN2_HUMAN |
| P17844 | 0,094189564 | 0,44142739 | 0,76607044 | DDX5_HUMAN |
| Q14204 | 0,094529871 | 0,33881001 | 0,70156419 | DYHC1_HUMAN |
| P46063 | 0,095078609 | 0,35338724 | 0,71280778 | RECQ1_HUMAN |
| P52434 | 0,095131646 | 0,73265802 | 0,90736714 | RPAB3_HUMAN |
| Q9NTZ6 | 0,095148556 | 0,4608357 | 0,7730431 | RBM12_HUMAN |
| P62753 | 0,095368998 | 0,3545159 | 0,71280778 | RS6_HUMAN |
| Q9UJA5 | 0,095502873 | 0,58416224 | 0,84446979 | TRM6_HUMAN |
| O75663 | 0,095528862 | 0,67213727 | 0,88551272 | TIPRL_HUMAN |
| P21796 | 0,095536232 | 0,32362361 | 0,69442115 | VDAC1_HUMAN |
| P35268 | 0,095555303 | 0,43182247 | 0,76142818 | RL22_HUMAN |
| P34932 | 0,095955704 | 0,32700805 | 0,69560058 | HSP74_HUMAN |
| O15126 | 0,096204759 | 0,42513483 | 0,76056043 | SCAM1_HUMAN |
| Q01130 | 0,096370789 | 0,39971056 | 0,74674937 | SRSF2_HUMAN |
| Q14203 | 0,096991128 | 0,40336815 | 0,74722994 | DCTN1_HUMAN |
| Q14694 | 0,097759174 | 0,53544517 | 0,81846545 | UBP10_HUMAN |
| P20042 | 0,098376334 | 0,31537117 | 0,68278635 | IF2B_HUMAN |
| P53350 | 0,098580663 | 0,57157652 | 0,83926118 | PLK1_HUMAN |
| P99999 | 0,098860476 | 0,72378198 | 0,90444117 | CYC_HUMAN |
| Q9NTJ3 | 0,099482189 | 0,64493969 | 0,8762009 | SMC4_HUMAN |
| P52758 | 0,101101401 | 0,64804938 | 0,87838922 | UK114_HUMAN |
| P51610 | 0,101300082 | 0,34809237 | 0,709092 | HCFC1_HUMAN |
| Q13418 | 0,101520227 | 0,71759771 | 0,90238683 | ILK_HUMAN |
| P60468 | 0,102071242 | 0,66359466 | 0,8803959 | SC61B_HUMAN |
| O75822 | 0,102466135 | 0,38632006 | 0,73612226 | EIF3J_HUMAN |
| O75608 | 0,102682485 | 0,43540932 | 0,76356053 | LYPA1_HUMAN |
| Q96KG9 | 0,102983207 | 0,62521088 | 0,86070535 | NTKL_HUMAN |
| P47755 | 0,103097339 | 0,43595211 | 0,76356053 | CAZA2_HUMAN |
| P62280 | 0,10321148 | 0,5259345 | 0,81780144 | RS11_HUMAN |
| Q8WUM4 | 0,104143092 | 0,3841378 | 0,73612226 | PDC6I_HUMAN |
| Q12904 | 0,104620525 | 0,68047194 | 0,89272795 | AIMP1_HUMAN |
| Q9NP97 | 0,104746945 | 0,4856944 | 0,79007031 | DLRB1_HUMAN |
| O60716 | 0,104771969 | 0,31216712 | 0,67919528 | CTND1_HUMAN |
| P50395 | 0,104868797 | 0,28461166 | 0,65783506 | GDIB_HUMAN |
| Q93062 | 0,105042444 | 0,47241301 | 0,78275407 | RBPMS_HUMAN |
| O43765 | 0,105492804 | 0,41906257 | 0,75742583 | SGTA_HUMAN |
| Q14498 | 0,105580455 | 0,57866592 | 0,83942825 | RBM39_HUMAN |
| P18669 | 0,105818646 | 0,44867634 | 0,76624848 | PGAM1_HUMAN |
| P62136 | 0,10616552 | 0,67827444 | 0,89052013 | PP1A_HUMAN |
| Q9Y520 | 0,106510244 | 0,30116718 | 0,66849988 | PRC2C_HUMAN |
| P28066 | 0,106839391 | 0,65911311 | 0,8803959 | PSA5_HUMAN |
| P26640 | 0,106901385 | 0,27863611 | 0,65090148 | SYVC_HUMAN |
| P42126 | 0,107387118 | 0,75637702 | 0,91407243 | ECI1_HUMAN |
| Q02790 | 0,107559632 | 0,43730971 | 0,76356053 | FKBP4_HUMAN |
| Q9UNZ2 | 0,107688805 | 0,42586539 | 0,76056043 | NSF1C_HUMAN |
| O00410 | 0,108314073 | 0,48896274 | 0,79007031 | IPO5_HUMAN |
| P21399 | 0,11022529 | 0,61900431 | 0,86061046 | ACOC_HUMAN |
| O95831 | 0,110732444 | 0,25081026 | 0,62782391 | AIFM1_HUMAN |
| Q6FI81 | 0,111299642 | 0,37224497 | 0,72792731 | CPIN1_HUMAN |
| P55072 | 0,111939291 | 0,25829104 | 0,63432137 | TERA_HUMAN |
| Q15046 | 0,112904355 | 0,24385605 | 0,62544131 | SYK_HUMAN |
| P10809 | 0,113055966 | 0,25105814 | 0,62782391 | CH60_HUMAN |
| P09651 | 0,113718887 | 0,65042456 | 0,87874148 | ROA1_HUMAN |
| O00231 | 0,114062834 | 0,28128946 | 0,65405547 | PSD11_HUMAN |
| P33992 | 0,114870055 | 0,23284778 | 0,61187801 | MCM5_HUMAN |
| Q9BQE3 | 0,114888605 | 0,57654255 | 0,83942825 | TBA1C_HUMAN |
| Q14344 | 0,114964637 | 0,43041011 | 0,76056043 | GNA13_HUMAN |
| P41252 | 0,114988595 | 0,32123255 | 0,69206718 | SYIC_HUMAN |
| O00233 | 0,115333477 | 0,35745475 | 0,71403323 | PSMD9_HUMAN |
| Q9Y6A5 | 0,115910685 | 0,26763146 | 0,64236665 | TACC3_HUMAN |
| Q9Y2A7 | 0,116604306 | 0,45386798 | 0,76943097 | NCKP1_HUMAN |
| P78344 | 0,117222124 | 0,25356228 | 0,63049856 | IF4G2_HUMAN |
| Q9Y5B9 | 0,117295982 | 0,39038614 | 0,73612226 | SP16H_HUMAN |
| P41250 | 0,117943631 | 0,42118505 | 0,75742583 | SYG_HUMAN |
| Q9P0K7 | 0,118097045 | 0,43780945 | 0,76356053 | RAI14_HUMAN |
| Q12888 | 0,118493848 | 0,41560662 | 0,75543026 | TP53B_HUMAN |
| P30038 | 0,118663209 | 0,56908933 | 0,83860775 | AL4A1_HUMAN |
| O60925 | 0,118965415 | 0,34972431 | 0,71057355 | PFD1_HUMAN |
| P09417 | 0,119132174 | 0,41047509 | 0,75403888 | DHPR_HUMAN |
| Q5JPE7 | 0,119603539 | 0,61901195 | 0,86061046 | NOMO2_HUMAN |
| P23246 | 0,119778708 | 0,24986674 | 0,62782391 | SFPQ_HUMAN |
| Q86V81 | 0,119928421 | 0,3283946 | 0,69664092 | THOC4_HUMAN |
| P83881 | 0,119976656 | 0,74751953 | 0,91241584 | RL36A_HUMAN |
| Q13011 | 0,120028048 | 0,30953192 | 0,67635742 | ECH1_HUMAN |
| Q8IXU6 | 0,120625048 | 0,62499553 | 0,86070535 | S35F2_HUMAN |
| Q01518 | 0,12077872 | 0,29991081 | 0,66787317 | CAP1_HUMAN |
| Q96N67 | 0,121713994 | 0,31653745 | 0,68446844 | DOCK7_HUMAN |
| Q9UDY8 | 0,122079019 | 0,42697529 | 0,76056043 | MALT1_HUMAN |
| Q15084 | 0,122964567 | 0,455777 | 0,76969834 | PDIA6_HUMAN |
| Q9Y295 | 0,123232733 | 0,25583489 | 0,63346159 | DRG1_HUMAN |
| Q15126 | 0,123683554 | 0,37734257 | 0,73138726 | PMVK_HUMAN |
| Q9GZS3 | 0,123945707 | 0,52784094 | 0,81780144 | WDR61_HUMAN |
| Q9Y678 | 0,124143612 | 0,39541669 | 0,74030089 | COPG1_HUMAN |
| Q9UJZ1 | 0,124210945 | 0,40509166 | 0,74884452 | STML2_HUMAN |
| P84157 | 0,12427608 | 0,45558153 | 0,76969834 | MXRA7_HUMAN |
| P36542 | 0,124339462 | 0,44561378 | 0,76624848 | ATPG_HUMAN |
| P30084 | 0,124775098 | 0,35003225 | 0,71057355 | ECHM_HUMAN |
| Q9H3U1 | 0,124788413 | 0,65945731 | 0,8803959 | UN45A_HUMAN |
| Q32P28 | 0,124814756 | 0,46734912 | 0,78006157 | P3H1_HUMAN |
| P25205 | 0,125196498 | 0,2199624 | 0,60302172 | MCM3_HUMAN |
| O43684 | 0,125254025 | 0,23825582 | 0,62144471 | BUB3_HUMAN |
| Q9UBX3 | 0,125634938 | 0,44947897 | 0,76642485 | DIC_HUMAN |
| O43818 | 0,125679814 | 0,30158126 | 0,66857484 | U3IP2_HUMAN |
| Q56VL3 | 0,125825156 | 0,66099392 | 0,8803959 | OCAD2_HUMAN |
| Q9H6T3 | 0,126236534 | 0,2521826 | 0,6291425 | RPAP3_HUMAN |
| P54687 | 0,126521019 | 0,75175361 | 0,91269533 | BCAT1_HUMAN |
| O94906 | 0,127496949 | 0,41255811 | 0,75543026 | PRP6_HUMAN |
| Q9Y3D8 | 0,127914116 | 0,53339821 | 0,81846545 | KAD6_HUMAN |
| Q99497 | 0,127961262 | 0,2765062 | 0,65036811 | PARK7_HUMAN |
| O75150 | 0,128399313 | 0,59913172 | 0,85492984 | BRE1B_HUMAN |
| Q9BUJ2 | 0,128438333 | 0,34102602 | 0,70520711 | HNRL1_HUMAN |
| P00390 | 0,129621728 | 0,21598786 | 0,59897319 | GSHR_HUMAN |
| P42224 | 0,129783069 | 0,1935051 | 0,57952634 | STAT1_HUMAN |
| Q9NQ55 | 0,129863075 | 0,39665389 | 0,74182717 | SSF1_HUMAN |
| P13861 | 0,129949594 | 0,35563572 | 0,71280778 | KAP2_HUMAN |
| P62841 | 0,130424155 | 0,79169108 | 0,92925413 | RS15_HUMAN |
| O75506 | 0,130435043 | 0,46456502 | 0,77707451 | HSBP1_HUMAN |
| Q7Z417 | 0,130937882 | 0,18432841 | 0,56902349 | NUFP2_HUMAN |
| O15160 | 0,131224121 | 0,43275808 | 0,76229764 | RPAC1_HUMAN |
| P13797 | 0,131231294 | 0,20109398 | 0,5839878 | PLST_HUMAN |
| Q9H8Y8 | 0,131408482 | 0,33520266 | 0,69903473 | GORS2_HUMAN |
| Q02952 | 0,131811711 | 0,22654491 | 0,60775412 | AKA12_HUMAN |
| Q15185 | 0,132159365 | 0,3152371 | 0,68278635 | TEBP_HUMAN |
| P37198 | 0,132352083 | 0,34457653 | 0,70652307 | NUP62_HUMAN |
| Q14974 | 0,132543381 | 0,26913504 | 0,64270609 | IMB1_HUMAN |
| Q8NE71 | 0,132579351 | 0,32994387 | 0,69717091 | ABCF1_HUMAN |
| P0CB43 | 0,132794328 | 0,41447815 | 0,75543026 | NA |
| Q7Z3T8 | 0,132825975 | 0,46914051 | 0,78076071 | ZFY16_HUMAN |
| P52594 | 0,133430627 | 0,71421361 | 0,90238683 | AGFG1_HUMAN |
| P33240 | 0,133819876 | 0,51357359 | 0,80901647 | CSTF2_HUMAN |
| P54727 | 0,133934366 | 0,33710804 | 0,70033965 | RD23B_HUMAN |
| P30040 | 0,134326329 | 0,2508364 | 0,62782391 | ERP29_HUMAN |
| P82650 | 0,134803997 | 0,3267287 | 0,69560058 | RT22_HUMAN |
| P07237 | 0,134984176 | 0,19421244 | 0,5798252 | PDIA1_HUMAN |
| P63000 | 0,13600714 | 0,3553017 | 0,71280778 | RAC1_HUMAN |
| P62917 | 0,136699634 | 0,19124785 | 0,57868111 | RL8_HUMAN |
| Q9NQW7 | 0,136783577 | 0,34944309 | 0,71057355 | XPP1_HUMAN |
| O75534 | 0,136837756 | 0,30004908 | 0,66787317 | CSDE1_HUMAN |
| P49257 | 0,137483295 | 0,56716456 | 0,83860775 | LMAN1_HUMAN |
| P25705 | 0,137610509 | 0,19830841 | 0,58298692 | ATPA_HUMAN |
| Q9P258 | 0,137700763 | 0,54523121 | 0,82030199 | RCC2_HUMAN |
| O15027 | 0,139168443 | 0,42091836 | 0,75742583 | SC16A_HUMAN |
| Q2NL82 | 0,1395535 | 0,64057569 | 0,87364784 | TSR1_HUMAN |
| Q12905 | 0,14030491 | 0,45941839 | 0,77213914 | ILF2_HUMAN |
| P30101 | 0,140570484 | 0,18380111 | 0,56902105 | PDIA3_HUMAN |
| P23193 | 0,141170687 | 0,20844699 | 0,59200291 | TCEA1_HUMAN |
| O14929 | 0,141440911 | 0,24516771 | 0,62737239 | HAT1_HUMAN |
| Q9UNX3 | 0,142179047 | 0,54749142 | 0,82053701 | RL26L_HUMAN |
| P11172 | 0,142301605 | 0,56594975 | 0,83860775 | UMPS_HUMAN |
| P49748 | 0,142707899 | 0,30354955 | 0,67162411 | ACADV_HUMAN |
| P55036 | 0,14287591 | 0,4335678 | 0,76229764 | PSMD4_HUMAN |
| Q9HC38 | 0,143131902 | 0,26248924 | 0,63737029 | GLOD4_HUMAN |
| P54886 | 0,143136955 | 0,19195493 | 0,57882807 | P5CS_HUMAN |
| P04818 | 0,143770681 | 0,56881121 | 0,83860775 | TYSY_HUMAN |
| Q9BQ52 | 0,143843963 | 0,42864562 | 0,76056043 | RNZ2_HUMAN |
| Q9Y2W2 | 0,144088065 | 0,44569927 | 0,76624848 | WBP11_HUMAN |
| P18583 | 0,144127323 | 0,29844181 | 0,66665908 | SON_HUMAN |
| P38919 | 0,144205159 | 0,21019744 | 0,59314142 | IF4A3_HUMAN |
| P54136 | 0,144316443 | 0,18179073 | 0,56564266 | SYRC_HUMAN |
| Q9Y3E5 | 0,144419528 | 0,51907537 | 0,81403613 | PTH2_HUMAN |
| Q13283 | 0,145582939 | 0,30887701 | 0,67635742 | G3BP1_HUMAN |
| P43487 | 0,14586074 | 0,20233436 | 0,5839878 | RANG_HUMAN |
| O15212 | 0,146484675 | 0,15357289 | 0,5201414 | PFD6_HUMAN |
| P36957 | 0,146662805 | 0,32100708 | 0,69206718 | ODO2_HUMAN |
| Q13242 | 0,147275535 | 0,33196849 | 0,6974601 | SRSF9_HUMAN |
| Q8N1F7 | 0,14745856 | 0,15429745 | 0,52064283 | NUP93_HUMAN |
| P54819 | 0,14887594 | 0,28068099 | 0,65355918 | KAD2_HUMAN |
| O76094 | 0,149111677 | 0,27885176 | 0,65090148 | SRP72_HUMAN |
| Q9Y2R9 | 0,149334033 | 0,56194973 | 0,83650095 | RT07_HUMAN |
| Q8NFJ5 | 0,149980094 | 0,68799089 | 0,89272795 | RAI3_HUMAN |
| Q15417 | 0,15033655 | 0,19824327 | 0,58298692 | CNN3_HUMAN |
| Q14008 | 0,152194413 | 0,19829707 | 0,58298692 | CKAP5_HUMAN |
| P62937 | 0,152249993 | 0,24321812 | 0,62544131 | PPIA_HUMAN |
| P04183 | 0,152495407 | 0,47412517 | 0,78485128 | KITH_HUMAN |
| O75153 | 0,152869207 | 0,13276031 | 0,50303967 | CLU_HUMAN |
| Q9BT78 | 0,154246026 | 0,29208107 | 0,66255294 | CSN4_HUMAN |
| Q9NZM1 | 0,154330045 | 0,12076241 | 0,48191514 | MYOF_HUMAN |
| P62877 | 0,155736429 | 0,36426386 | 0,71933616 | RBX1_HUMAN |
| P00813 | 0,155836112 | 0,29673108 | 0,66452252 | ADA_HUMAN |
| Q9BZZ5 | 0,155956268 | 0,37435617 | 0,72842986 | API5_HUMAN |
| O14744 | 0,156197533 | 0,3097086 | 0,67635742 | ANM5_HUMAN |
| P53597 | 0,156748273 | 0,37015672 | 0,72464979 | SUCA_HUMAN |
| Q9BWU0 | 0,15706313 | 0,33793388 | 0,70057518 | NADAP_HUMAN |
| Q9BXV9 | 0,158178808 | 0,6663201 | 0,8803959 | CN142_HUMAN |
| P63010 | 0,158247104 | 0,29163101 | 0,66255294 | AP2B1_HUMAN |
| P48444 | 0,158269046 | 0,14659819 | 0,51236505 | COPD_HUMAN |
| Q9UQ35 | 0,15826944 | 0,1993584 | 0,5839878 | SRRM2_HUMAN |
| P49327 | 0,158583442 | 0,14589044 | 0,51236505 | FAS_HUMAN |
| P61970 | 0,158984692 | 0,35627086 | 0,71280778 | NTF2_HUMAN |
| P13804 | 0,159008619 | 0,28163822 | 0,65405547 | ETFA_HUMAN |
| Q9UBI6 | 0,159149811 | 0,33742189 | 0,70033965 | GBG12_HUMAN |
| Q969X5 | 0,159193287 | 0,7006673 | 0,8952766 | ERGI1_HUMAN |
| P00441 | 0,159578145 | 0,19317366 | 0,57952098 | SODC_HUMAN |
| P52597 | 0,159832284 | 0,10498761 | 0,45524659 | HNRPF_HUMAN |
| Q9Y3I0 | 0,15984456 | 0,29667916 | 0,66452252 | RTCB_HUMAN |
| Q16543 | 0,161168219 | 0,18861246 | 0,57466327 | CDC37_HUMAN |
| P07919 | 0,161343628 | 0,20263513 | 0,5839878 | QCR6_HUMAN |
| Q9H4M9 | 0,161698922 | 0,19160609 | 0,57876892 | EHD1_HUMAN |
| Q9P0V9 | 0,161734695 | 0,38990161 | 0,73612226 | SEP10_HUMAN |
| Q9UBQ7 | 0,162137747 | 0,23255457 | 0,61187801 | GRHPR_HUMAN |
| P26599 | 0,163234902 | 0,28894456 | 0,66141216 | PTBP1_HUMAN |
| Q9Y6Y8 | 0,163245478 | 0,17020321 | 0,543044 | S23IP_HUMAN |
| P16615 | 0,163267928 | 0,10723808 | 0,46094021 | AT2A2_HUMAN |
| Q9UNQ2 | 0,164320528 | 0,13855096 | 0,50533731 | DIM1_HUMAN |
| O75694 | 0,16473229 | 0,23793744 | 0,62144471 | NU155_HUMAN |
| Q8TEQ6 | 0,16517118 | 0,1178235 | 0,47622986 | GEMI5_HUMAN |
| Q9H6Z4 | 0,165172092 | 0,3590815 | 0,71403323 | RANB3_HUMAN |
| P23588 | 0,165174526 | 0,27916935 | 0,65090148 | IF4B_HUMAN |
| P29317 | 0,165268896 | 0,22912274 | 0,60845586 | EPHA2_HUMAN |
| Q9BZE4 | 0,165598797 | 0,29334653 | 0,66337984 | NOG1_HUMAN |
| O96008 | 0,165599697 | 0,27657082 | 0,65036811 | TOM40_HUMAN |
| O15371 | 0,165750474 | 0,09809396 | 0,44565474 | EIF3D_HUMAN |
| Q9P2X0 | 0,16633701 | 0,2440573 | 0,62544131 | DPM3_HUMAN |
| Q92990 | 0,167106286 | 0,2465905 | 0,62782391 | GLMN_HUMAN |
| Q9BUF5 | 0,167347672 | 0,15956361 | 0,52279992 | TBB6_HUMAN |
| O60502 | 0,167690658 | 0,59613779 | 0,85465044 | OGA_HUMAN |
| O60763 | 0,16821139 | 0,20540616 | 0,58716102 | USO1_HUMAN |
| Q6PIU2 | 0,168820826 | 0,24093749 | 0,62544131 | NCEH1_HUMAN |
| Q08945 | 0,170035644 | 0,12943855 | 0,50198119 | SSRP1_HUMAN |
| P35232 | 0,17035907 | 0,10264968 | 0,45002029 | PHB_HUMAN |
| Q9UKK9 | 0,171422608 | 0,26820087 | 0,64236665 | NUDT5_HUMAN |
| Q9UI26 | 0,17243418 | 0,69486004 | 0,89491865 | IPO11_HUMAN |
| P27105 | 0,172590341 | 0,26739107 | 0,64236665 | STOM_HUMAN |
| Q9H074 | 0,173635847 | 0,47505167 | 0,7853205 | PAIP1_HUMAN |
| Q8NC51 | 0,173805407 | 0,20464385 | 0,58689051 | PAIRB_HUMAN |
| Q99961 | 0,174208645 | 0,15150566 | 0,51818474 | SH3G1_HUMAN |
| Q5SW79 | 0,17559107 | 0,65908057 | 0,8803959 | CE170_HUMAN |
| Q96GG9 | 0,177027179 | 0,48675439 | 0,79007031 | DCNL1_HUMAN |
| Q9Y676 | 0,177181119 | 0,11013534 | 0,46569592 | RT18B_HUMAN |
| Q6UN15 | 0,177713173 | 0,09821349 | 0,44565474 | FIP1_HUMAN |
| P24534 | 0,177978222 | 0,41427517 | 0,75543026 | EF1B_HUMAN |
| Q9UGP8 | 0,178978552 | 0,27763376 | 0,65036811 | SEC63_HUMAN |
| Q15036 | 0,179232422 | 0,11977572 | 0,47991871 | SNX17_HUMAN |
| P62857 | 0,179539179 | 0,14754238 | 0,51293778 | RS28_HUMAN |
| Q05682 | 0,179704196 | 0,14080968 | 0,50830271 | CALD1_HUMAN |
| O00161 | 0,180041449 | 0,40300827 | 0,74722994 | SNP23_HUMAN |
| P61009 | 0,180470908 | 0,24209049 | 0,62544131 | SPCS3_HUMAN |
| O60232 | 0,180515976 | 0,42599655 | 0,76056043 | SSA27_HUMAN |
| Q9Y6E2 | 0,180654277 | 0,43080407 | 0,76056043 | BZW2_HUMAN |
| Q9NZB2 | 0,180665397 | 0,45640733 | 0,76974061 | F120A_HUMAN |
| Q9BX40 | 0,181164939 | 0,25781613 | 0,63432137 | LS14B_HUMAN |
| O75821 | 0,181416432 | 0,2475851 | 0,62782391 | EIF3G_HUMAN |
| P31943 | 0,182428121 | 0,1533723 | 0,5201414 | HNRH1_HUMAN |
| P61086 | 0,182482388 | 0,38811277 | 0,73612226 | UBE2K_HUMAN |
| P49189 | 0,183183241 | 0,19882907 | 0,58354176 | AL9A1_HUMAN |
| P38646 | 0,183529388 | 0,07352069 | 0,41426079 | GRP75_HUMAN |
| Q96G03 | 0,1836034 | 0,39042639 | 0,73612226 | PGM2_HUMAN |
| P61019 | 0,183610022 | 0,10057078 | 0,44988149 | RAB2A_HUMAN |
| Q9NWY4 | 0,185005212 | 0,4377449 | 0,76356053 | CD027_HUMAN |
| Q9H0A0 | 0,185067017 | 0,09802548 | 0,44565474 | NAT10_HUMAN |
| Q6L8Q7 | 0,185682742 | 0,1969469 | 0,58298692 | PDE12_HUMAN |
| P62191 | 0,186056656 | 0,10513658 | 0,45524659 | PRS4_HUMAN |
| Q14137 | 0,186232261 | 0,09770704 | 0,44565474 | BOP1_HUMAN |
| Q13573 | 0,187416679 | 0,26379578 | 0,63824731 | SNW1_HUMAN |
| P10515 | 0,18747885 | 0,11255616 | 0,4677866 | ODP2_HUMAN |
| Q9BY44 | 0,188109807 | 0,08044352 | 0,42518835 | EIF2A_HUMAN |
| O15067 | 0,188192386 | 0,2467339 | 0,62782391 | PUR4_HUMAN |
| P78406 | 0,188488609 | 0,26126578 | 0,63615683 | RAE1L_HUMAN |
| P17096 | 0,188564972 | 0,36876261 | 0,72397536 | HMGA1_HUMAN |
| Q92878 | 0,188613349 | 0,25656969 | 0,63432137 | RAD50_HUMAN |
| Q92552 | 0,188667749 | 0,48202525 | 0,78974874 | RT27_HUMAN |
| Q8WVJ2 | 0,189086031 | 0,25931682 | 0,63442907 | NUDC2_HUMAN |
| O95425 | 0,19008659 | 0,18855796 | 0,57466327 | SVIL_HUMAN |
| Q14019 | 0,192114639 | 0,10172796 | 0,45002029 | COTL1_HUMAN |
| P40926 | 0,192306034 | 0,09786124 | 0,44565474 | MDHM_HUMAN |
| Q96P16 | 0,193021685 | 0,2899032 | 0,66255294 | RPR1A_HUMAN |
| Q14181 | 0,193299626 | 0,29678018 | 0,66452252 | DPOA2_HUMAN |
| P04075 | 0,193815295 | 0,15700153 | 0,52149753 | ALDOA_HUMAN |
| O75390 | 0,195647363 | 0,09733595 | 0,44565474 | CISY_HUMAN |
| P98175 | 0,19588483 | 0,21492186 | 0,59878389 | RBM10_HUMAN |
| Q9H773 | 0,196484923 | 0,56634167 | 0,83860775 | DCTP1_HUMAN |
| P50552 | 0,196605513 | 0,15810081 | 0,52279992 | VASP_HUMAN |
| P11387 | 0,197116623 | 0,07952262 | 0,42518835 | TOP1_HUMAN |
| Q14247 | 0,197212082 | 0,12481543 | 0,48978914 | SRC8_HUMAN |
| P46937 | 0,198600014 | 0,5404001 | 0,81949948 | YAP1_HUMAN |
| Q99459 | 0,198693965 | 0,12315536 | 0,48653286 | CDC5L_HUMAN |
| O14745 | 0,19899225 | 0,09655653 | 0,44565474 | NHRF1_HUMAN |
| P14868 | 0,199030972 | 0,06674313 | 0,40114205 | SYDC_HUMAN |
| Q96PK6 | 0,199137216 | 0,12789284 | 0,49963471 | RBM14_HUMAN |
| Q16658 | 0,199173214 | 0,38157253 | 0,73553125 | FSCN1_HUMAN |
| Q9UBP6 | 0,199236368 | 0,22794835 | 0,60775412 | TRMB_HUMAN |
| Q6IAA8 | 0,199273954 | 0,13711308 | 0,50533731 | LTOR1_HUMAN |
| P11310 | 0,199290388 | 0,08221061 | 0,42518835 | ACADM_HUMAN |
| P23526 | 0,19967063 | 0,0715154 | 0,40869439 | SAHH_HUMAN |
| Q14739 | 0,200565798 | 0,17772872 | 0,5590205 | LBR_HUMAN |
| Q99567 | 0,201685478 | 0,32066526 | 0,69206718 | NUP88_HUMAN |
| P00533 | 0,202418081 | 0,35528992 | 0,71280778 | EGFR_HUMAN |
| O00193 | 0,202419826 | 0,26398255 | 0,63824731 | SMAP_HUMAN |
| Q0ZGT2 | 0,202468866 | 0,26980495 | 0,64270609 | NEXN_HUMAN |
| Q9P287 | 0,203006416 | 0,41583788 | 0,75543026 | BCCIP_HUMAN |
| Q96FX7 | 0,203007528 | 0,38528938 | 0,73612226 | TRM61_HUMAN |
| O43504 | 0,203721115 | 0,1464548 | 0,51236505 | LTOR5_HUMAN |
| Q92841 | 0,204093807 | 0,09074658 | 0,44314581 | DDX17_HUMAN |
| Q9UI30 | 0,204233842 | 0,06283969 | 0,39738194 | TR112_HUMAN |
| Q14966 | 0,204779835 | 0,20525077 | 0,58716102 | ZN638_HUMAN |
| Q9NQT5 | 0,20502766 | 0,19267441 | 0,57952098 | EXOS3_HUMAN |
| O00264 | 0,205072056 | 0,15229216 | 0,51885583 | PGRC1_HUMAN |
| P22695 | 0,205301265 | 0,15560075 | 0,52149753 | QCR2_HUMAN |
| Q9H0U4 | 0,205302841 | 0,27773817 | 0,65036811 | RAB1B_HUMAN |
| Q7Z478 | 0,205752425 | 0,12574495 | 0,49233769 | DHX29_HUMAN |
| Q15843 | 0,205789049 | 0,19001461 | 0,57594082 | NEDD8_HUMAN |
| Q13671 | 0,205870909 | 0,18757434 | 0,57348817 | RIN1_HUMAN |
| O95747 | 0,206124844 | 0,07419641 | 0,41572965 | OXSR1_HUMAN |
| P49792 | 0,206389435 | 0,22692628 | 0,60775412 | RBP2_HUMAN |
| P05386 | 0,207246606 | 0,06672141 | 0,40114205 | RLA1_HUMAN |
| Q06124 | 0,207596156 | 0,13805663 | 0,50533731 | PTN11_HUMAN |
| O14974 | 0,208003135 | 0,06348859 | 0,39814517 | MYPT1_HUMAN |
| Q9NQR4 | 0,208791521 | 0,32722507 | 0,69560058 | NIT2_HUMAN |
| Q96HQ2 | 0,209088445 | 0,22178001 | 0,60546408 | C2AIL_HUMAN |
| Q15165 | 0,210068125 | 0,25462375 | 0,63135198 | PON2_HUMAN |
| Q9Y282 | 0,21029381 | 0,53462646 | 0,81846545 | ERGI3_HUMAN |
| P62873 | 0,210540478 | 0,28665161 | 0,65938922 | GBB1_HUMAN |
| Q96K17 | 0,211229363 | 0,1485127 | 0,51293778 | BT3L4_HUMAN |
| P07737 | 0,211288224 | 0,16901686 | 0,54122338 | PROF1_HUMAN |
| Q9UMS4 | 0,211875382 | 0,38577524 | 0,73612226 | PRP19_HUMAN |
| P80723 | 0,212157924 | 0,35580745 | 0,71280778 | BASP1_HUMAN |
| P31942 | 0,213154537 | 0,09056641 | 0,44314581 | HNRH3_HUMAN |
| P62263 | 0,215652044 | 0,2014441 | 0,5839878 | RS14_HUMAN |
| P84103 | 0,215956257 | 0,40397081 | 0,74755861 | SRSF3_HUMAN |
| P40925 | 0,216311446 | 0,11605002 | 0,47430215 | MDHC_HUMAN |
| Q13405 | 0,216420412 | 0,20333411 | 0,58504316 | RM49_HUMAN |
| P09382 | 0,216508648 | 0,1091948 | 0,46569592 | LEG1_HUMAN |
| Q00577 | 0,217415946 | 0,14833068 | 0,51293778 | PURA_HUMAN |
| P09486 | 0,217581566 | 0,45716742 | 0,76974061 | SPRC_HUMAN |
| O60271 | 0,217709867 | 0,07935805 | 0,42518835 | JIP4_HUMAN |
| Q96RT1 | 0,217862025 | 0,0981399 | 0,44565474 | LAP2_HUMAN |
| Q9BQG0 | 0,219405569 | 0,06259793 | 0,39728215 | MBB1A_HUMAN |
| Q9BTE6 | 0,219547356 | 0,32994664 | 0,69717091 | AASD1_HUMAN |
| Q13492 | 0,220143721 | 0,16577005 | 0,5357054 | PICAL_HUMAN |
| Q15717 | 0,220745776 | 0,13336533 | 0,50303967 | ELAV1_HUMAN |
| Q9NYL9 | 0,221134955 | 0,16842144 | 0,54030089 | TMOD3_HUMAN |
| Q5SSJ5 | 0,222488418 | 0,13745487 | 0,50533731 | HP1B3_HUMAN |
| P04181 | 0,223862539 | 0,10603786 | 0,45694389 | OAT_HUMAN |
| O43795 | 0,22387305 | 0,09016422 | 0,44314581 | MYO1B_HUMAN |
| O60506 | 0,22681688 | 0,11584688 | 0,47430215 | HNRPQ_HUMAN |
| P35606 | 0,227861479 | 0,30542314 | 0,67200736 | COPB2_HUMAN |
| P63151 | 0,228345931 | 0,10144258 | 0,45002029 | 2ABA_HUMAN |
| P13073 | 0,230128386 | 0,1729678 | 0,54887616 | COX41_HUMAN |
| P63165 | 0,23025898 | 0,30605158 | 0,67254835 | SUMO1_HUMAN |
| O00232 | 0,230331815 | 0,13382447 | 0,50303967 | PSD12_HUMAN |
| Q92769 | 0,230667618 | 0,42009317 | 0,75742583 | HDAC2_HUMAN |
| Q5TFE4 | 0,231509725 | 0,40554214 | 0,74888979 | NT5D1_HUMAN |
| O95394 | 0,231550607 | 0,06831858 | 0,4071324 | AGM1_HUMAN |
| P46087 | 0,231575203 | 0,06411649 | 0,39829252 | NOP2_HUMAN |
| O96019 | 0,231605939 | 0,21330704 | 0,59617453 | ACL6A_HUMAN |
| P14209 | 0,231768274 | 0,34617044 | 0,70756912 | CD99_HUMAN |
| P61221 | 0,232090753 | 0,03312485 | 0,32693324 | ABCE1_HUMAN |
| P22307 | 0,232322883 | 0,09780214 | 0,44565474 | NLTP_HUMAN |
| Q8NEJ9 | 0,23366247 | 0,32850701 | 0,69664092 | NGDN_HUMAN |
| P46459 | 0,23400702 | 0,20212065 | 0,5839878 | NSF_HUMAN |
| Q9H8H0 | 0,234036226 | 0,11074132 | 0,46569592 | NOL11_HUMAN |
| Q5T4S7 | 0,234958359 | 0,06409243 | 0,39829252 | UBR4_HUMAN |
| P35241 | 0,236334327 | 0,11153697 | 0,46625672 | RADI_HUMAN |
| Q1KMD3 | 0,236956683 | 0,05969419 | 0,38735155 | HNRL2_HUMAN |
| Q96A33 | 0,237121916 | 0,09748433 | 0,44565474 | CCD47_HUMAN |
| Q9Y2X3 | 0,237830089 | 0,06347427 | 0,39814517 | NOP58_HUMAN |
| Q969X6 | 0,23786935 | 0,1044898 | 0,4546858 | CIR1A_HUMAN |
| P35580 | 0,238883902 | 0,06363981 | 0,39814517 | MYH10_HUMAN |
| Q9HC07 | 0,240391814 | 0,3343669 | 0,69895007 | TM165_HUMAN |
| Q9NZT2 | 0,240417632 | 0,16758308 | 0,53904265 | OGFR_HUMAN |
| P00403 | 0,241243139 | 0,04908796 | 0,36117836 | COX2_HUMAN |
| Q9Y277 | 0,243190466 | 0,08528697 | 0,43333667 | VDAC3_HUMAN |
| Q9BSJ8 | 0,243508011 | 0,04262203 | 0,34514615 | ESYT1_HUMAN |
| P08648 | 0,247114035 | 0,1899025 | 0,57594082 | ITA5_HUMAN |
| Q9BXJ9 | 0,247131922 | 0,07541789 | 0,4203846 | NAA15_HUMAN |
| P62942 | 0,248786219 | 0,07425433 | 0,41572965 | FKB1A_HUMAN |
| Q9UMX0 | 0,24971337 | 0,28291686 | 0,65569977 | UBQL1_HUMAN |
| Q9NVX2 | 0,250427214 | 0,24816532 | 0,62782391 | NLE1_HUMAN |
| Q9BYG3 | 0,25139856 | 0,20441213 | 0,58689051 | MK67I_HUMAN |
| P12814 | 0,252107867 | 0,03895238 | 0,34239145 | ACTN1_HUMAN |
| Q13277 | 0,252832987 | 0,21932153 | 0,60302172 | STX3_HUMAN |
| Q14061 | 0,252866376 | 0,77914636 | 0,92406404 | COX17_HUMAN |
| Q9UBU9 | 0,253106463 | 0,10770732 | 0,46182795 | NXF1_HUMAN |
| P12270 | 0,253249134 | 0,05157201 | 0,3641108 | TPR_HUMAN |
| P67809 | 0,253781646 | 0,08185975 | 0,42518835 | YBOX1_HUMAN |
| Q9BQA1 | 0,254063989 | 0,05642056 | 0,37857763 | MEP50_HUMAN |
| O76071 | 0,2561565 | 0,48612352 | 0,79007031 | CIAO1_HUMAN |
| P46379 | 0,257248086 | 0,12907888 | 0,50198119 | BAG6_HUMAN |
| Q12874 | 0,257607933 | 0,08990801 | 0,44314581 | SF3A3_HUMAN |
| Q14118 | 0,258043153 | 0,24888192 | 0,62782391 | DAG1_HUMAN |
| Q92544 | 0,258229887 | 0,11074389 | 0,46569592 | TM9S4_HUMAN |
| P43897 | 0,258370576 | 0,08623624 | 0,43536006 | EFTS_HUMAN |
| P61289 | 0,258456212 | 0,02180687 | 0,28544594 | PSME3_HUMAN |
| Q9ULV4 | 0,2584812 | 0,02558827 | 0,29614469 | COR1C_HUMAN |
| Q9NUQ3 | 0,259533282 | 0,26113135 | 0,63615683 | TXLNG_HUMAN |
| Q9UHV9 | 0,259994112 | 0,22701186 | 0,60775412 | PFD2_HUMAN |
| P17812 | 0,260411277 | 0,03539593 | 0,33454862 | PYRG1_HUMAN |
| Q99798 | 0,260436656 | 0,09825213 | 0,44565474 | ACON_HUMAN |
| P32969 | 0,261374355 | 0,26675774 | 0,64236665 | RL9_HUMAN |
| Q8NFH5 | 0,261662165 | 0,13976863 | 0,50830271 | NUP53_HUMAN |
| Q9Y2K7 | 0,262382678 | 0,49862152 | 0,79836423 | KDM2A_HUMAN |
| O00622 | 0,263728238 | 0,05096689 | 0,36336059 | CYR61_HUMAN |
| P31946 | 0,264939119 | 0,06970031 | 0,40869439 | 1433B_HUMAN |
| Q15435 | 0,265417563 | 0,05542964 | 0,37531168 | PP1R7_HUMAN |
| P28331 | 0,266262315 | 0,40866191 | 0,75306881 | NDUS1_HUMAN |
| Q16186 | 0,266474852 | 0,03492049 | 0,33313144 | ADRM1_HUMAN |
| Q9P2E9 | 0,266483993 | 0,03119769 | 0,32121512 | RRBP1_HUMAN |
| Q9GZT3 | 0,267210868 | 0,34266391 | 0,70652307 | SLIRP_HUMAN |
| Q9BSV6 | 0,267567101 | 0,22351888 | 0,60546408 | SEN34_HUMAN |
| P07339 | 0,267668204 | 0,03461293 | 0,33251109 | CATD_HUMAN |
| Q5T9A4 | 0,267698613 | 0,03197534 | 0,32121512 | ATD3B_HUMAN |
| P51858 | 0,269269799 | 0,05572034 | 0,37531168 | HDGF_HUMAN |
| Q9NS69 | 0,269356526 | 0,11984318 | 0,47991871 | TOM22_HUMAN |
| Q14839 | 0,269757505 | 0,27582166 | 0,65036811 | CHD4_HUMAN |
| O14776 | 0,270087216 | 0,2314537 | 0,61003838 | TCRG1_HUMAN |
| Q92900 | 0,270149047 | 0,04963553 | 0,36167674 | RENT1_HUMAN |
| O00566 | 0,271523057 | 0,13281637 | 0,50303967 | MPP10_HUMAN |
| Q9C002 | 0,271595833 | 0,25075714 | 0,62782391 | NMES1_HUMAN |
| Q14690 | 0,272691815 | 0,0296164 | 0,31747341 | RRP5_HUMAN |
| P52565 | 0,273521606 | 0,16177731 | 0,52667503 | GDIR1_HUMAN |
| O95202 | 0,273650257 | 0,04999286 | 0,36167674 | LETM1_HUMAN |
| Q9UKV3 | 0,274569973 | 0,10330855 | 0,45149125 | ACINU_HUMAN |
| Q14157 | 0,27500649 | 0,0437519 | 0,34758102 | UBP2L_HUMAN |
| P51665 | 0,275108931 | 0,21794445 | 0,60171177 | PSMD7_HUMAN |
| Q9UHY1 | 0,275597251 | 0,10005428 | 0,44872276 | NRBP_HUMAN |
| O15347 | 0,277430331 | 0,06970376 | 0,40869439 | HMGB3_HUMAN |
| P46060 | 0,277722677 | 0,03572896 | 0,33477272 | RAGP1_HUMAN |
| Q13066 | 0,278083614 | 0,01535891 | 0,25000899 | GAG2B_HUMAN |
| Q9Y3Y2 | 0,27820897 | 0,43924838 | 0,76379689 | CHTOP_HUMAN |
| P62158 | 0,278661782 | 0,11594818 | 0,47430215 | CALM_HUMAN |
| P37108 | 0,279418544 | 0,23054284 | 0,60917264 | SRP14_HUMAN |
| Q9HAV4 | 0,279466772 | 0,01822979 | 0,26931065 | XPO5_HUMAN |
| P48147 | 0,280266754 | 0,20179448 | 0,5839878 | PPCE_HUMAN |
| Q15181 | 0,280858118 | 0,10205593 | 0,45002029 | IPYR_HUMAN |
| O95239 | 0,281008701 | 0,26110737 | 0,63615683 | KIF4A_HUMAN |
| Q13065 | 0,281351877 | 0,14057766 | 0,50830271 | GAGE1_HUMAN |
| P28799 | 0,282553005 | 0,38480359 | 0,73612226 | GRN_HUMAN |
| P67870 | 0,28313257 | 0,09881917 | 0,44565474 | CSK2B_HUMAN |
| P48507 | 0,283509894 | 0,13798291 | 0,50533731 | GSH0_HUMAN |
| Q9UHB9 | 0,283769456 | 0,01755066 | 0,2659057 | SRP68_HUMAN |
| Q13347 | 0,285161137 | 0,15665372 | 0,52149753 | EIF3I_HUMAN |
| Q07021 | 0,285174555 | 0,07044817 | 0,40869439 | C1QBP_HUMAN |
| Q9NVD7 | 0,285406518 | 0,04562084 | 0,35511786 | PARVA_HUMAN |
| P62306 | 0,286915805 | 0,02817919 | 0,31108198 | RUXF_HUMAN |
| Q96TA1 | 0,287488354 | 0,05036629 | 0,36288503 | NIBL1_HUMAN |
| P42285 | 0,287521675 | 0,09512054 | 0,44565474 | SK2L2_HUMAN |
| O75116 | 0,287838029 | 0,13491888 | 0,50376636 | ROCK2_HUMAN |
| Q9NR30 | 0,289322391 | 0,01870385 | 0,27174688 | DDX21_HUMAN |
| Q02241 | 0,289340503 | 0,12187791 | 0,48365997 | KIF23_HUMAN |
| Q7KZ85 | 0,290131277 | 0,04605624 | 0,35511786 | SPT6H_HUMAN |
| O60884 | 0,290730065 | 0,02839204 | 0,31108198 | DNJA2_HUMAN |
| P26368 | 0,290936391 | 0,01763685 | 0,2659057 | U2AF2_HUMAN |
| P62834 | 0,291049047 | 0,07609731 | 0,42042351 | RAP1A_HUMAN |
| O00267 | 0,291137774 | 0,11712415 | 0,47552945 | SPT5H_HUMAN |
| Q9H3N1 | 0,291437831 | 0,04555534 | 0,35511786 | TMX1_HUMAN |
| O75947 | 0,291481183 | 0,04037048 | 0,34498692 | ATP5H_HUMAN |
| Q9Y5M8 | 0,292652996 | 0,15112903 | 0,51790418 | SRPRB_HUMAN |
| Q96KR1 | 0,298176943 | 0,03949149 | 0,34283422 | ZFR_HUMAN |
| P19338 | 0,299735453 | 0,02848931 | 0,31108198 | NUCL_HUMAN |
| Q99700 | 0,299859706 | 0,08734663 | 0,43536006 | ATX2_HUMAN |
| Q8WXF1 | 0,300783677 | 0,03588301 | 0,33477272 | PSPC1_HUMAN |
| Q04721 | 0,302368401 | 0,26808752 | 0,64236665 | NOTC2_HUMAN |
| Q9BTE3 | 0,302529326 | 0,19671575 | 0,58298692 | MCMBP_HUMAN |
| Q9C0C9 | 0,302663759 | 0,07093213 | 0,40869439 | UBE2O_HUMAN |
| O75940 | 0,302960629 | 0,03692513 | 0,33986585 | SPF30_HUMAN |
| P29692 | 0,303680158 | 0,05180772 | 0,36431189 | EF1D_HUMAN |
| Q7L2E3 | 0,304076843 | 0,13397415 | 0,50303967 | DHX30_HUMAN |
| Q86SX6 | 0,304275628 | 0,15503637 | 0,52113564 | GLRX5_HUMAN |
| Q15061 | 0,305502168 | 0,26430264 | 0,63824731 | WDR43_HUMAN |
| Q13067 | 0,305627451 | 0,39002319 | 0,73612226 | GAGE3_HUMAN |
| Q12974 | 0,306376306 | 0,22784221 | 0,60775412 | TP4A2_HUMAN |
| P36776 | 0,307615463 | 0,05690286 | 0,37924458 | LONM_HUMAN |
| P29966 | 0,307925937 | 0,14124324 | 0,50882299 | MARCS_HUMAN |
| Q9UKG1 | 0,308214209 | 0,07160289 | 0,40869439 | DP13A_HUMAN |
| Q13501 | 0,308373526 | 0,06607141 | 0,40114205 | SQSTM_HUMAN |
| O15397 | 0,308855553 | 0,4510751 | 0,76712487 | IPO8_HUMAN |
| P52907 | 0,309993107 | 0,05825681 | 0,38072669 | CAZA1_HUMAN |
| P61026 | 0,310213945 | 0,24729704 | 0,62782391 | RAB10_HUMAN |
| Q8NCA5 | 0,310251077 | 0,0548801 | 0,37531168 | FA98A_HUMAN |
| P51991 | 0,312005022 | 0,03309207 | 0,32693324 | ROA3_HUMAN |
| P27694 | 0,312094452 | 0,11016394 | 0,46569592 | RFA1_HUMAN |
| Q9BR76 | 0,315010952 | 0,01784805 | 0,2659057 | COR1B_HUMAN |
| Q9UQ80 | 0,316004179 | 0,01002376 | 0,20305363 | PA2G4_HUMAN |
| Q14126 | 0,316323401 | 0,03941638 | 0,34283422 | DSG2_HUMAN |
| P61981 | 0,320381032 | 0,2113623 | 0,59329651 | 1433G_HUMAN |
| O75396 | 0,320683473 | 0,06123084 | 0,39286064 | SC22B_HUMAN |
| Q7Z3U7 | 0,321023378 | 0,05541539 | 0,37531168 | MON2_HUMAN |
| Q5VTR2 | 0,323051769 | 0,21215191 | 0,59389022 | BRE1A_HUMAN |
| P53041 | 0,324496298 | 0,07910209 | 0,42518835 | PPP5_HUMAN |
| O76024 | 0,324643676 | 0,09619729 | 0,44565474 | WFS1_HUMAN |
| O60216 | 0,325355474 | 0,10349885 | 0,45149125 | RAD21_HUMAN |
| P05455 | 0,326919124 | 0,03165067 | 0,32121512 | LA_HUMAN |
| P19387 | 0,327217836 | 0,04602887 | 0,35511786 | RPB3_HUMAN |
| Q7Z4S6 | 0,327641478 | 0,01076883 | 0,20890123 | KI21A_HUMAN |
| O95232 | 0,328728942 | 0,32678711 | 0,69560058 | LC7L3_HUMAN |
| Q93009 | 0,329226632 | 0,07811388 | 0,42518835 | UBP7_HUMAN |
| P20073 | 0,332692419 | 0,42810428 | 0,76056043 | ANXA7_HUMAN |
| P18754 | 0,33295828 | 0,06015186 | 0,38735155 | RCC1_HUMAN |
| O15511 | 0,333831109 | 0,07038442 | 0,40869439 | ARPC5_HUMAN |
| P46013 | 0,334471074 | 0,01845052 | 0,27030014 | KI67_HUMAN |
| Q6Y7W6 | 0,33580216 | 0,03734686 | 0,33999874 | PERQ2_HUMAN |
| Q9NVP1 | 0,336628382 | 0,09145851 | 0,44383025 | DDX18_HUMAN |
| Q15836 | 0,337409043 | 0,18239533 | 0,5665212 | VAMP3_HUMAN |
| Q9H0S4 | 0,339222045 | 0,05552781 | 0,37531168 | DDX47_HUMAN |
| Q8WZA0 | 0,339299567 | 0,13248843 | 0,50303967 | LZIC_HUMAN |
| P30085 | 0,340091141 | 0,03870736 | 0,34224384 | KCY_HUMAN |
| Q13310 | 0,340326295 | 0,00850654 | 0,19754389 | PABP4_HUMAN |
| P34949 | 0,340327711 | 0,36498457 | 0,71933616 | MPI_HUMAN |
| P62495 | 0,341496989 | 0,02426133 | 0,2941477 | ERF1_HUMAN |
| P61106 | 0,341518036 | 0,02095427 | 0,28120308 | RAB14_HUMAN |
| Q6GMV3 | 0,344494451 | 0,1000565 | 0,44872276 | PTRD1_HUMAN |
| Q9NXH9 | 0,345060693 | 0,05132104 | 0,36379992 | TRM1_HUMAN |
| P15927 | 0,346421434 | 0,19684987 | 0,58298692 | RFA2_HUMAN |
| Q5SY16 | 0,347477653 | 0,02767143 | 0,30973139 | NOL9_HUMAN |
| P00491 | 0,347505179 | 0,18384753 | 0,56902105 | PNPH_HUMAN |
| P78324 | 0,347939844 | 0,12296244 | 0,48653286 | SHPS1_HUMAN |
| O60488 | 0,348282497 | 0,11711753 | 0,47552945 | ACSL4_HUMAN |
| Q13428 | 0,350761524 | 0,10111565 | 0,45002029 | TCOF_HUMAN |
| Q9NQS7 | 0,351614036 | 0,14814928 | 0,51293778 | INCE_HUMAN |
| P24539 | 0,353229071 | 0,48385007 | 0,79007031 | AT5F1_HUMAN |
| Q13409 | 0,353889031 | 0,04907537 | 0,36117836 | DC1I2_HUMAN |
| Q86Y56 | 0,355855115 | 0,02518663 | 0,29518733 | DAAF5_HUMAN |
| P19623 | 0,358380598 | 0,03126145 | 0,32121512 | SPEE_HUMAN |
| P61586 | 0,3586543 | 0,14621065 | 0,51236505 | RHOA_HUMAN |
| Q9BQ67 | 0,359037984 | 0,12453644 | 0,48978761 | GRWD1_HUMAN |
| P61006 | 0,359454248 | 0,08631648 | 0,43536006 | RAB8A_HUMAN |
| Q9H0D6 | 0,360868888 | 0,0058184 | 0,18051136 | XRN2_HUMAN |
| Q9BTT0 | 0,361483543 | 0,09333341 | 0,44565474 | AN32E_HUMAN |
| Q7Z4Q2 | 0,362000149 | 0,04187808 | 0,34514615 | HEAT3_HUMAN |
| O00469 | 0,363088811 | 0,00411294 | 0,16290903 | PLOD2_HUMAN |
| Q9NUQ6 | 0,364511813 | 0,02876005 | 0,31209983 | SPS2L_HUMAN |
| Q9Y2T2 | 0,364530032 | 0,34482184 | 0,70652307 | AP3M1_HUMAN |
| O75330 | 0,367580815 | 0,04253228 | 0,34514615 | HMMR_HUMAN |
| Q9Y570 | 0,377929525 | 0,04153895 | 0,34514615 | PPME1_HUMAN |
| P26196 | 0,378208145 | 0,02037776 | 0,28120308 | DDX6_HUMAN |
| Q03252 | 0,378253942 | 0,00710146 | 0,1863338 | LMNB2_HUMAN |
| P61224 | 0,379703518 | 0,20729753 | 0,59064677 | RAP1B_HUMAN |
| Q9HAV7 | 0,380103073 | 0,03874091 | 0,34224384 | GRPE1_HUMAN |
| Q92973 | 0,38232124 | 0,03832624 | 0,34224384 | TNPO1_HUMAN |
| Q53GS9 | 0,390682851 | 0,08350482 | 0,42551153 | SNUT2_HUMAN |
| P25325 | 0,39394814 | 0,0574208 | 0,37924458 | THTM_HUMAN |
| P42166 | 0,396723737 | 0,00348914 | 0,15727965 | LAP2A_HUMAN |
| Q86XP3 | 0,3969299 | 0,19456104 | 0,5798252 | DDX42_HUMAN |
| Q9H583 | 0,398705858 | 0,00496232 | 0,17632654 | HEAT1_HUMAN |
| Q08J23 | 0,398873427 | 0,02093216 | 0,28120308 | NSUN2_HUMAN |
| P54709 | 0,399393255 | 0,15931915 | 0,52279992 | AT1B3_HUMAN |
| P08621 | 0,401120829 | 0,06631808 | 0,40114205 | RU17_HUMAN |
| Q8WVY7 | 0,401664902 | 0,1304848 | 0,50303967 | UBCP1_HUMAN |
| P62070 | 0,403920903 | 0,02751844 | 0,30973139 | RRAS2_HUMAN |
| Q9H814 | 0,40463843 | 0,23044732 | 0,60917264 | PHAX_HUMAN |
| Q00534 | 0,408721998 | 0,09331731 | 0,44565474 | CDK6_HUMAN |
| P13987 | 0,408780302 | 0,07131335 | 0,40869439 | CD59_HUMAN |
| P61960 | 0,409414764 | 0,01448103 | 0,24716165 | UFM1_HUMAN |
| Q01081 | 0,413703001 | 0,29692899 | 0,66452252 | U2AF1_HUMAN |
| Q14103 | 0,414647763 | 0,17673793 | 0,55781918 | HNRPD_HUMAN |
| Q9H061 | 0,416187326 | 0,07580314 | 0,4203846 | T126A_HUMAN |
| O75937 | 0,416705562 | 0,03751977 | 0,33999874 | DNJC8_HUMAN |
| P09497 | 0,417180984 | 0,00257044 | 0,15727965 | CLCB_HUMAN |
| P21127 | 0,420148151 | 0,03328842 | 0,32693324 | CD11B_HUMAN |
| O75521 | 0,42204396 | 0,15828631 | 0,52279992 | ECI2_HUMAN |
| Q16222 | 0,42392451 | 0,01935519 | 0,2766376 | UAP1_HUMAN |
| P45973 | 0,424952196 | 0,21160234 | 0,59329651 | CBX5_HUMAN |
| Q9NW13 | 0,425313942 | 0,0226209 | 0,28731937 | RBM28_HUMAN |
| Q9GZL7 | 0,425432914 | 0,00933302 | 0,20009089 | WDR12_HUMAN |
| P82673 | 0,427625785 | 0,01248297 | 0,22186579 | RT35_HUMAN |
| P50502 | 0,429611567 | 0,00273919 | 0,15727965 | F10A1_HUMAN |
| Q13151 | 0,431203081 | 0,14344754 | 0,51231408 | ROA0_HUMAN |
| Q9GZR7 | 0,431469646 | 0,03599092 | 0,33477272 | DDX24_HUMAN |
| Q8NF91 | 0,432131868 | 0,22795507 | 0,60775412 | SYNE1_HUMAN |
| Q02218 | 0,435295405 | 0,00343656 | 0,15727965 | ODO1_HUMAN |
| Q16630 | 0,437964022 | 0,01249415 | 0,22186579 | CPSF6_HUMAN |
| Q9NRN7 | 0,441694423 | 0,04720556 | 0,35925268 | ADPPT_HUMAN |
| Q9Y3L5 | 0,442375902 | 0,02560523 | 0,29614469 | RAP2C_HUMAN |
| P09601 | 0,444970166 | 0,00750678 | 0,19125963 | HMOX1_HUMAN |
| P02786 | 0,445415769 | 0,00274646 | 0,15727965 | TFR1_HUMAN |
| Q96C90 | 0,447276219 | 0,08291551 | 0,42518835 | PP14B_HUMAN |
| Q15785 | 0,450526271 | 0,00417003 | 0,16290903 | TOM34_HUMAN |
| Q96DI7 | 0,45332489 | 0,08726072 | 0,43536006 | SNR40_HUMAN |
| Q16576 | 0,457113235 | 0,12963564 | 0,50198119 | RBBP7_HUMAN |
| P16989 | 0,458246734 | 0,03189578 | 0,32121512 | YBOX3_HUMAN |
| P08195 | 0,458431414 | 0,05799567 | 0,3804343 | 4F2_HUMAN |
| Q16629 | 0,459121355 | 0,04356589 | 0,34758102 | SRSF7_HUMAN |
| Q6DKJ4 | 0,459250167 | 0,0664071 | 0,40114205 | NXN_HUMAN |
| Q9Y5S9 | 0,459707437 | 0,00501498 | 0,17632654 | RBM8A_HUMAN |
| Q9Y3Z3 | 0,461159027 | 0,04822977 | 0,36117836 | SAMH1_HUMAN |
| Q96PU5 | 0,462033309 | 0,04529285 | 0,35511786 | NED4L_HUMAN |
| P18031 | 0,465564095 | 0,00900328 | 0,20006487 | PTN1_HUMAN |
| Q9Y2R5 | 0,466611743 | 0,10148658 | 0,45002029 | RT17_HUMAN |
| O00629 | 0,479639548 | 0,04700367 | 0,35925268 | IMA3_HUMAN |
| Q96IZ0 | 0,480596971 | 0,00146157 | 0,12492383 | PAWR_HUMAN |
| Q9H488 | 0,480769058 | 0,02360544 | 0,29019833 | OFUT1_HUMAN |
| Q92692 | 0,493150561 | 0,08309334 | 0,42518835 | PVRL2_HUMAN |
| O15269 | 0,49392555 | 0,02199733 | 0,28544594 | SPTC1_HUMAN |
| Q9Y5L4 | 0,497405523 | 0,14639145 | 0,51236505 | TIM13_HUMAN |
| Q9NX58 | 0,502149245 | 0,08975943 | 0,44314581 | LYAR_HUMAN |
| Q96D46 | 0,503304976 | 0,14454368 | 0,51231408 | NMD3_HUMAN |
| P35244 | 0,503335673 | 0,0345243 | 0,33251109 | RFA3_HUMAN |
| A6NDG6 | 0,503751296 | 0,22021612 | 0,60302172 | PGP_HUMAN |
| Q16763 | 0,506938815 | 0,00094785 | 0,10414467 | UBE2S_HUMAN |
| Q05048 | 0,508692122 | 0,02477498 | 0,29518733 | CSTF1_HUMAN |
| P10644 | 0,509339486 | 0,00919624 | 0,20006487 | KAP0_HUMAN |
| Q9H1E3 | 0,510371487 | 0,07859388 | 0,42518835 | NUCKS_HUMAN |
| P09493 | 0,512956291 | 0,19720063 | 0,58298692 | TPM1_HUMAN |
| Q92945 | 0,514558152 | 0,00220869 | 0,15531532 | FUBP2_HUMAN |
| P08240 | 0,517679746 | 0,00158161 | 0,12638512 | SRPR_HUMAN |
| Q8IUD2 | 0,519937381 | 0,11503072 | 0,47430215 | RB6I2_HUMAN |
| P15374 | 0,521977349 | 0,02271752 | 0,28731937 | UCHL3_HUMAN |
| P19404 | 0,526130337 | 0,02601457 | 0,29891248 | NDUV2_HUMAN |
| P21283 | 0,527968504 | 0,11967661 | 0,47991871 | VATC1_HUMAN |
| Q9UKY7 | 0,532952544 | 0,00584058 | 0,18051136 | CDV3_HUMAN |
| Q96AC1 | 0,541197819 | 0,00615822 | 0,18349405 | FERM2_HUMAN |
| Q69YN2 | 0,541444428 | 0,22731166 | 0,60775412 | C19L1_HUMAN |
| Q5BKZ1 | 0,561942347 | 0,00300944 | 0,15727965 | ZN326_HUMAN |
| Q6NUK1 | 0,565094609 | 0,05099251 | 0,36336059 | SCMC1_HUMAN |
| Q16204 | 0,566114985 | 0,08301873 | 0,42518835 | CCDC6_HUMAN |
| Q15637 | 0,567421884 | 0,06950998 | 0,40869439 | SF01_HUMAN |
| Q92530 | 0,568943822 | 0,048607 | 0,36117836 | PSMF1_HUMAN |
| Q9BVJ6 | 0,577035958 | 0,0667534 | 0,40114205 | UT14A_HUMAN |
| O94973 | 0,578610725 | 0,08951013 | 0,44314581 | AP2A2_HUMAN |
| P00367 | 0,588374932 | 0,05252546 | 0,36497926 | DHE3_HUMAN |
| P61204 | 0,606797204 | 0,01086535 | 0,20890123 | ARF3_HUMAN |
| Q92879 | 0,642268976 | 0,13078413 | 0,50303967 | CELF1_HUMAN |
| Q6RFH5 | 0,656458338 | 0,15869672 | 0,52279992 | WDR74_HUMAN |
| Q92804 | 0,65794589 | 0,16252235 | 0,52812254 | RBP56_HUMAN |
| P03956 | 0,65947673 | 0,0032367 | 0,15727965 | MMP1_HUMAN |
| Q9Y312 | 0,663846976 | 0,17775453 | 0,5590205 | AAR2_HUMAN |
| O14617 | 0,668025863 | 0,10235619 | 0,45002029 | AP3D1_HUMAN |
| P14635 | 0,673542773 | 0,0150071 | 0,25000899 | CCNB1_HUMAN |
| P11388 | 0,682751058 | 0,00054828 | 0,08032323 | TOP2A_HUMAN |
| Q9H501 | 0,698070824 | 0,00037662 | 0,08032323 | ESF1_HUMAN |
| Q96I24 | 0,702284924 | 0,0014667 | 0,12492383 | FUBP3_HUMAN |
| Q96QD8 | 0,721004462 | 0,00872268 | 0,19914888 | S38A2_HUMAN |
| Q9BRP1 | 0,741163495 | 0,00284629 | 0,15727965 | PDD2L_HUMAN |
| Q9UI42 | 0,741567824 | 0,00854001 | 0,19754389 | CBPA4_HUMAN |
| Q9Y5J7 | 0,752409982 | 0,00232429 | 0,15715779 | TIM9_HUMAN |
| Q9NUQ9 | 0,755571782 | 0,00692104 | 0,1863338 | FA49B_HUMAN |
| Q9P035 | 0,759124722 | 0,00143272 | 0,12492383 | HACD3_HUMAN |
| Q969G3 | 0,766420203 | 0,05385049 | 0,37125163 | SMCE1_HUMAN |
| Q15437 | 0,767441695 | 0,00101332 | 0,10478948 | SC23B_HUMAN |
| O94760 | 0,782676485 | 0,00690763 | 0,1863338 | DDAH1_HUMAN |
| O75607 | 0,783219728 | 0,00769629 | 0,19328676 | NPM3_HUMAN |
| Q9ULW0 | 0,803453446 | 7,14E-05 | 0,04182953 | TPX2_HUMAN |
| P21980 | 0,806458348 | 0,00051554 | 0,08032323 | TGM2_HUMAN |
| P24928 | 0,808662706 | 0,01004873 | 0,20305363 | RPB1_HUMAN |
| P53814 | 0,840950498 | 0,00527634 | 0,18036487 | SMTN_HUMAN |
| O14907 | 0,84958316 | 0,00370485 | 0,16085146 | TX1B3_HUMAN |
| P29992 | 0,91177934 | 0,07054658 | 0,40869439 | GNA11_HUMAN |
| Q9BS26 | 0,971714852 | 0,00435839 | 0,16302233 | ERP44_HUMAN |
| P61513 | 0,983972061 | 0,0616739 | 0,39426441 | RL37A_HUMAN |
| Q9UKA9 | 0,999421233 | 0,0045918 | 0,16817467 | PTBP2_HUMAN |
| Q99661 | 1,028898034 | 0,01092308 | 0,20890123 | KIF2C_HUMAN |
| Q9NQW6 | 1,079326388 | 0,0395878 | 0,34283422 | ANLN_HUMAN |
| P04908 | 1,167055939 | 0,0250581 | 0,29518733 | H2A1B_HUMAN |
